# Reporting of financial conflicts of interest in clinical practice guidelines: a case study analysis of guidelines from the Canadian Medical Association Infobase

**DOI:** 10.1186/s12913-016-1646-5

**Published:** 2016-08-15

**Authors:** Adrienne Shnier, Joel Lexchin, Mirna Romero, Kevin Brown

**Affiliations:** 1 School of Health Policy and Management, Faculty of Health, York University, Toronto, ON Canada; 2 University Health Network, Toronto, ON Canada; 3 Epidemiology Division, University of Utah, Salt Lake City, USA

**Keywords:** Financial conflicts of interest, Disclosure, Clinical practice guidelines, Medicine and the pharmaceutical industry, Treatment recommendations

## Abstract

**Background:**

Clinical practice guidelines are widely distributed by medical associations and relied upon by physicians for the best available clinical evidence. International findings report that financial conflicts of interest (FCOI) with drug companies may influence drug recommendations and are common among guideline authors. There is no comparable study on exclusively Canadian guidelines; therefore, we provide a case study of authors’ FCOI declarations in guidelines from the Canadian Medical Association (CMA) Infobase. We also assess the financial relationships between guideline-affiliated organizations and drug companies.

**Methods:**

Using a population approach, we extracted first-line drug recommendations and authors’ FCOI disclosures in guidelines from the CMA Infobase. We contacted the corresponding authors on guidelines when FCOI disclosures were missing for some or all authors. We also extracted guideline-affiliated organizations and searched each of their websites to determine if they had financial relationships with drug companies.

**Results:**

We analyzed 350 authors from 28 guidelines. Authors were named on one, two, or three guidelines, yielding 400 FCOI statements. In 75.0 % of guidelines at least one author, and in 21.4 % of guidelines all authors, disclosed FCOI with drug companies. In 54.0 % of guidelines at least one author, and in 28.6 % of guidelines over half of the authors, disclosed FCOI with manufacturers of drugs that they recommended. Twenty of 48 authors on multiple guidelines reported different FCOI in their disclosures. Eight guidelines identified affiliated organizations with financial relationships with manufacturers of drugs recommended in those guidelines.

**Conclusions:**

This is the first study to systematically describe FCOI disclosures by authors of Canadian guidelines and financial relationships between guideline-affiliated organizations and pharmaceutical companies. These financial relationships are common. Because authoritative value is assigned to guidelines distributed by medical associations, we encourage them to develop formal policies to limit the potential influence of FCOI on guideline recommendations.

**Electronic supplementary material:**

The online version of this article (doi:10.1186/s12913-016-1646-5) contains supplementary material, which is available to authorized users.

## Background

Clinicians rely on clinical practice guidelines (CPGs) for guidance when making treatment decisions for patients. Although CPGs should be based on critical analysis of the best available scientific evidence, authors’ recommendations in some guidelines have been based on lower levels of evidence or expert opinion [1]. Therefore, recommendations may be vulnerable to biases [2], which are of particular concern since financial ties are common among guideline authors, committee members, and drug companies that manufacture medications recommended in guidelines [3]. A common finding in the literature analyzing guideline recommendations is that the presence of financial conflict of interest (FCOI) relationships with pharmaceutical companies may have the potential to influence drug recommendations [4–13]. Furthermore, international literature has demonstrated concern over underreporting and inconsistencies in FCOI disclosures in guidelines [2, 3, 14–17].

CPGs are widely distributed by professional medical associations, such as the Canadian Medical Association (CMA). The CMA Infobase (https://www.cma.ca/En/Pages/clinical-practice-guidelines.aspx) lists guidelines that meet the following criteria: include information to help patients and physicians make decisions about appropriate health care for specific clinical circumstances; be produced by an authoritative Canadian organization or if produced outside of Canada be officially endorsed by such an organization; have been developed or reviewed in the last 5 years; and have evidence that a literature search was performed during guideline development [18].

We present a case study of authors’ FCOI disclosure statements in guidelines from the CMA Infobase. We determine the prevalence of not only authors’ disclosed FCOI with drug companies in general, but also their FCOI disclosures with the manufacturers of the on-patent drugs that they recommend as first-line treatments in their respective guidelines. Our focus on on-patent drugs rests on the assumption that recommending an on-patent drug is directly beneficial to a single manufacturer, as compared to recommending an off-patent drug, produced by multiple manufacturers. Finally, we determine the frequency with which the guideline-affiliated organizations have financial relationships with pharmaceutical companies that are also manufacturers of the drugs recommended as first-line treatments in those guidelines.

## Methods

Using a population approach, we analyzed 1,150 guidelines listed in the CMA Infobase. We did not limit our case study of guidelines by medical specialty or disease category; however, we limited the eligible guidelines to the 353 listed on the CMA Infobase [19] that were published or most recently reviewed between 01 January 2012 and 06 November 2013, inclusive (see Additional file 1). We imposed this date restriction because the requirement for FCOI disclosure is a relatively recent phenomenon in guideline production [20]. French-language guidelines and those that could not be accessed on the web were excluded.

**Fig. 1:**
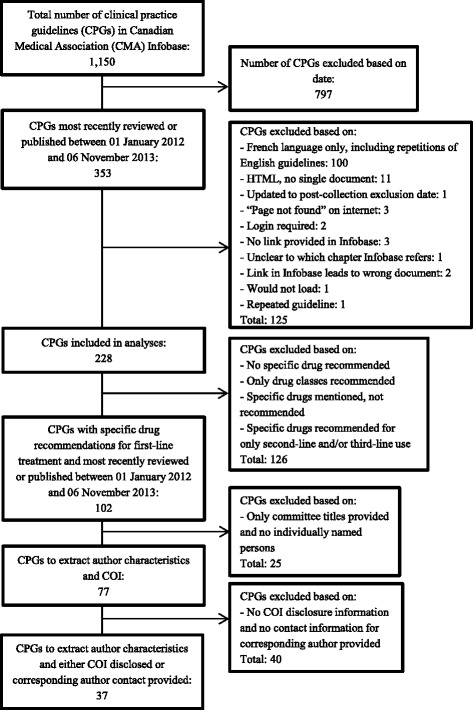
Guideline exclusion criteria and process of guideline exclusion

Two pairs of study researchers (AS and MR, JL and SA) assessed and documented whether guidelines recommended specific drugs based on recommendation tables or, in their absence, within the text. We considered a “recommendation” to have been made when authors stated that one or more specific medications were appropriate first-line treatments for a particular patient population. We excluded guidelines that either recommended only drug classes as opposed to specific medications, or mentioned or acknowledged specific drugs without making clear first-line recommendations (Fig. 1).

Specific drugs for first-line treatment were recommended in 102 guidelines. Guidelines that provided only titles of organizations, committees, or associations in lieu of individually named authors or committee members were excluded, leaving 77 guidelines. Forty additional guidelines that provided neither disclosures, nor corresponding authors’ contact information were excluded (Fig. 1). Any disagreements or uncertainties were resolved through discussion.

**Fig. 2:**
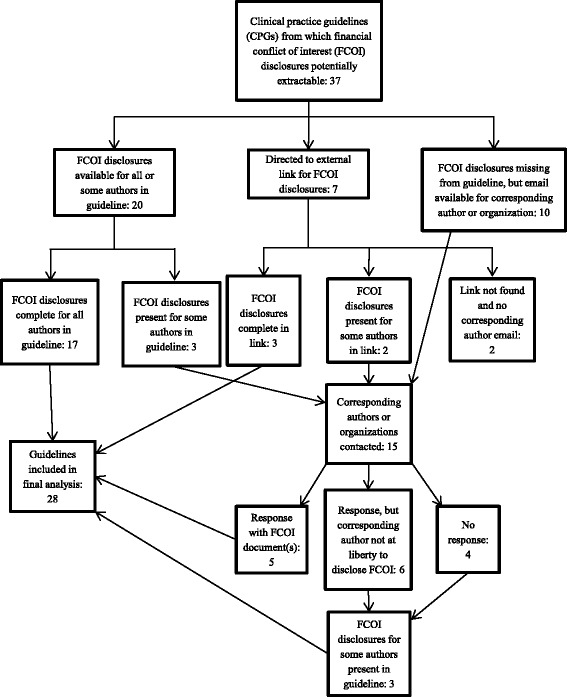
Summary and results of locating disclosure statements

From the remaining 37 guidelines, we attempted to locate disclosure statements for the authors. Twenty guidelines provided FCOI disclosure statements for all or some of the authors named on the guideline. Disclosure statements were absent in 10 guidelines and seven guidelines provided links to FCOI disclosure statements on external websites. We successfully accessed five of these external webpages (Fig. 2). We contacted the corresponding authors on 15 guidelines for one of two reasons: (1) the guideline had no FCOI disclosure section and there was no indication that all authors were either free of FCOI or had any conflicts to report (10 guidelines), or (2) disclosures were either vague, or missing for some authors and the guideline did not state that these authors were free of FCOI (5 guidelines). We received responses from 11 of the 15 corresponding authors whom we contacted, but only five provided us with additional FCOI disclosure statements.

Ultimately, we located FCOI disclosures for all of the authors on 22 guidelines and some authors on 6 guidelines yielding 350 unique authors, of whom 48 were named on two or three guidelines, resulting in a total of 400 disclosure statements (see Additional file 2). We divided FCOI disclosures with pharmaceutical companies into two groups – relevant and non-relevant. We considered FCOI to be “relevant” when they existed between an author and the manufacturer of a patented drug recommended for first-line treatment in that guideline. “Non-relevant” FCOI were those with a drug company other than the manufacturer of one of the recommended drugs [16]. These companies may have produced a drug that could also be used to treat the condition being discussed in the guideline but they may also have produced a drug that was not useful for the condition. We did not attempt to distinguish between the two situations as that would have involved analyzing every drug made by the company and then using expert opinion to decide if one (or more) of these drugs could have been recommended.

We considered FCOI to include not only financial compensation, but also activities that are generally associated with gifting, payment, or reimbursement, even if a monetary value was not disclosed. We defined “vague” FCOI disclosures as situations when financial ties were present, but the declaration prevented a clear determination of the number of pharmaceutical companies with which authors held FCOI and whether those FCOI could be classified as relevant or non-relevant. Conflicts with “non-commercial” organizations were defined as ties that authors disclosed with not-for-profit organizations such as the Canadian Agency for Drugs and Technologies in Health (CADTH).

Because of resource limitations, we decided *a priori* to extract FCOI disclosure information for a maximum of 25 authors per guideline, including chairs, co-chairs, principle authors, co-authors, and committee members. We assumed that all committee members who were named within the guideline had voted on its recommendations, even if they were not explicitly listed as authors. We also assumed that anyone who was not identified as an author or named committee member (i.e., reviewers, consultants, and liaisons) did not vote on the final recommendations in the guidelines and we excluded them. When more than 25 authors and/or committee members, which will hereafter be referred to collectively as authors, were named on a guideline, we assigned each a random value using Microsoft Excel [21]. Organized in ascending numerical order, the top 25, automatically including explicitly identified chair(s), co-chair(s), and principal author(s), were included in our analysis. We included these groups because we considered that they had the most influence in the final recommendations and, therefore, the presence or absence of their FCOI was particularly important. However, due to their small numbers we did not analyze chairs, co-chairs and principal authors separately. We also extracted authors’ demographic information from the guidelines: names, academic and medical degrees, and hospital and academic affiliations.

We recorded whether the medications recommended in the guidelines were on-patent or if there were off-patent versions available in Canada by consulting the Compendium of Pharmaceuticals and Specialties (CPS) and Health Canada’s Drug Products Database [22–24] for the years that the guidelines were either published or reviewed to determine whether authors’ FCOI declarations were relevant or non-relevant.

Finally, we identified the guideline-affiliated organizations. We visited each of the organizations’ websites to identify the pharmaceutical companies with which they disclosed having financial relationships. We did not examine whether conferences held by these organizations had pharmaceutical company sponsors.

This study has received ethics approval from the Ethics Review Board at York University and conforms to the standards of the Tri-Council Research Ethics guidelines (Certificate #: 2014-186). Written informed consent for participation in this study was obtained from participants.

## Results

We obtained FCOI disclosures for authors on 28 guidelines. Twelve were most recently reviewed or published in 2013 and 16 in 2012.

Out of 400 FCOI disclosure statements for 350 unique authors, 188 (47.0 %) declared FCOI with pharmaceutical companies. Individual authors declared FCOI with up to 19 drug companies (median: 3, interquartile range [IQR]: 0, 8). Out of these 188 FCOI declarations, 97 were relevant, 65 were non-relevant, and 26 were vague. Two-hundred and twelve (53.0 %) of the 400 declarations stated that the authors were either free of FCOI with drug companies or had conflicts with only non-commercial organizations (Table 1).

**Table 1:** Summary of financial conflict of interest disclosures by guideline

Clinical practice guideline ID#	Year	On-patent drugs recommended (N)	Off-patent drugs recommended (N)	Disclosure statements assessed (N)	Assessed statements disclosing drug company FCOIs^a^, N (%)	Assessed statements disclosing relevant FCOIs, N (%)	Assessed statements disclosing non-relevant FCOIs, N (%)	Assessed statements disclosing vague FCOIs, N (%)	Assessed statements disclosing no FCOI or non-commercial conflicts, N (%)
5	2013	1	1	2	0 (0)	0 (0)	0 (0)	0 (0)	2 (100)
7	2013	2	8	19	18 (95)	15 (79)	3 (16)	0 (0)	1 (5)
18	2013	0	4	22	0 (0)	0 (0)	0 (0)	0 (0)	22 (100)
27	2013	4	9	21	19 (90)	18 (86)	1 (5)	0 (0)	2 (10)
29	2013	5	7	25	18 (72)	6 (24)	12 (48)	0 (0)	7 (28)
35	2013	3	2	5	2 (40)	2 (40)	0 (0)	0 (0)	3 (60)
40	2013	3	3	13	0 (0)	0 (0)	0 (0)	0 (0)	13 (100)
44	2013	6	3	13	0 (0)	0 (0)	0 (0)	0 (0)	13 (100)
46	2013	7	0	9	9 (100)	4 (44)	0 (0)	5 (56)	0 (0)
93	2013	1	0	19	19 (100)	0 (0)	0 (0)	19 (100)	0 (0)
94	2013	3	15	22	15 (68)	10 (45)	5 (23)	0 (0)	7 (32)
103	2013	0	1	17	12 (71)	0 (0)	12 (35)	0 (0)	5 (29)
112	2012	2	0	4	4 (100)	3 (75)	1 (25)	0 (0)	0 (0)
242	2012	8	6	9	8 (89)	7 (78)	1 (11)	0 (0)	1 (11)
244	2012	0	1	19	8 (42)	0 (0)	8 (42)	0 (0)	11 (58)
258	2012	1	0	9	8 (89)	6 (67)	2 (22)	0 (0)	1 (11)
260	2012	1	2	3	3 (100)	3 (100)	0 (0)	0 (0)	0 (0)
267	2012	1	0	2	2 (100)	0 (0)	0 (0)	2 (100)	0 (0)
269	2012	4	1	24	6 (25)	6 (25)	0 (0)	0 (0)	18 (75)
273	2012	1	1	23	2 (9)	2 (9)	0 (0)	0 (0)	21 (91)
274	2012	0	1	24	18 (75)	0 (0)	18 (75)	0 (0)	6 (25)
283	2012	2	0	13	0 (0)	0 (0)	0 (0)	0 (0)	13 (100)
289	2012	2	0	23	1 (4)	1 (4)	0 (0)	0 (0)	22 (96)
295	2012	3	1	25	1 (4)	0 (0)	1 (4)	0 (0)	24 (96)
299	2012	8	1	16	12 (75)	12 (75)	0 (0)	0 (0)	4 (25)
345	2012	2	1	8	0 (0)	0 (0)	0 (0)	0 (0)	8 (100)
349	2012	7	0	3	3 (100)	2 (67)	1 (33)	0 (0)	0 (0)
352	2012	1	2	8	0 (0)	0 (0)	0 (0)	0 (0)	8 (100)
Totals				400	188	97	65	26	212

^a^Financial conflicts of interest

### Author-level analysis

Three-hundred and two unique authors (86.3 %) were each on one guideline, while 46 (13.1 %) were each on two guidelines and two (0.6 %) were each on three guidelines.

Of the authors on one guideline, 119 (34.0 %) disclosed FCOI with drug companies, while 162 (46.3 %) disclosed that they had either conflicts with non-commercial organizations or were free of FCOI with drug companies. Twenty-one (6.0 %) disclosed vague FCOI with drug companies (Table 2).

**Table 2:** Unique authors’ declarations in one, two, and three guidelines

Type of declaration	Number of unique authors making declarations in:		
	One guideline	Two guidelines	Three guidelines
FCOI^a^ with drug companies	119	7	0
Non-commercial conflicts or no FCOI	162	21	0
Vague FCOI	21	0	0
FCOI with different drug companies	0	12	0
FCOI with drug companies in one or guideline, then vague FCOI in another guideline	0	1	0
FCOI with drug companies in one guideline, then non-commercial conflicts/no FCOI in another guideline	0	3	0
Non-commercial conflicts/no FCOI in one or two guidelines and vague FCOI in one or two guidelines	0	2	2
Total number of unique authors: 350			

^a^Financial conflicts of interest

Twenty-eight of the 48 authors’ declarations on two or three guidelines were consistent in their disclosure statements, but 20 disclosed different FCOI in their disclosure statements in two or three guidelines. Authors whose disclosures differed in their multiple statements declared a combination of the following disclosure types: FCOI with different drug companies, vague FCOI with drug companies, conflicts with only non-commercial organizations, and no FCOI (Table 2).

### Guideline-level analysis

In 21 guidelines (75.0 %) at least one author disclosed FCOI with drug companies, while in six guidelines (21.4 %) all authors disclosed FCOI with drug companies (median: 69.4 %, IQR: 3.0 %, 93.1 %) (Table 1).

In 15 guidelines (54.0 %) at least one author disclosed relevant FCOI (median: 6.5 %, IQR: 0 %, 66.7 %), while in one guideline (3.6 %) all authors disclosed relevant FCOI. In eight guidelines (28.6 %), over half of the authors declared relevant FCOI (Table 1).

The majority of guidelines identified affiliations with organizations (26/28, 93.0 %). In total, 39 organizations were found. Nineteen of the 39 organizations (49.0 %) identified financial relationships with pharmaceutical companies on their respective websites. In eight guidelines (26.0 %), at least one drug recommended for first-line treatment was manufactured by a pharmaceutical company listed on the affiliated organizations’ website (see Additional file 3).

## Discussion

In this study of 28 Canadian guidelines produced or revised since the start of 2012, we found that FCOI relationships between guideline authors and drug companies are common. Authors disclosed FCOI with drug companies in 21 guidelines (75.0 %). Relevant financial ties are also common amongst guideline authors, as authors in 15 guidelines (54.0 %) reported FCOI with manufacturers of drugs that they recommend as first-line treatments. Twenty authors on two or three guidelines disclosed different FCOI in their statements. Eight guidelines identified affiliated organizations that had financial relationships with drug companies that manufactured drugs recommended for first-line treatment.

To our knowledge, our study is the first to systematically describe FCOI disclosures by authors on Canadian guidelines, as well as the financial relationships between the guideline-affiliated organizations and pharmaceutical companies. We used a population approach to guideline inclusion and did not exclude guidelines based on medical specialty or disease category.

This study contributes to existing international studies on FCOI disclosures across medical specialties, which have produced results similar to our findings. Cosgrove and colleagues found that in three psychiatry guidelines, 18 of 20 (90 %) authors held FCOI with pharmaceutical companies and none of these ties were disclosed in the guideline. On two of the three guidelines assessed, 100 % of the working group members possessed FCOI [6]. Neuman and colleagues found that in 14 guidelines on screening and/or treatment for hyperlipidaemia or diabetes published by national Canadian and American organizations between 2000 and 2010, 138 out of 288 (48.0 %) panel members reported FCOI [25].

In a study analyzing 17 cardiovascular guidelines, Mendelson and colleagues found that 277 out of 498 (56.0 %) authors reported FCOI [20]. A 2013 study by Norris and colleagues found that in 13 guidelines for glycemic control in type 2 diabetes mellitus from the National Guideline Clearinghouse (NGC), the percentage of authors who disclosed one or more FCOI ranged from 0 to 94 % [16]. A 2013 Danish study found that 135 out of 254 (53.1 %) authors on 45 guidelines held FCOI and although FCOI were common, disclosures were rare [2].

We believe that our results provide a conservative estimate of the prevalence of FCOI disclosed by guideline authors as we did not conduct external web or publication searches to determine the completeness of the FCOI disclosures in the guidelines. Our exclusion of 40 guidelines based on their lack of both FCOI disclosure sections and corresponding author contact information reflects findings that guidelines commonly contain no information about potential FCOI [26].

Finally, consistent with related research [2, 27], 20 authors on two or three guidelines that we assessed disclosed different FCOI in their disclosures. These inconsistencies may be due to five factors: (i) journals in which these guidelines were published may have had different FCOI disclosure policies and requirements, (ii) endorsing professional medical societies and associations, as well as the medical journals in which CPGs are published, may have had differing policies on FCOI disclosure and permitted relationships, (iii) authors may have engaged in new FCOI relationships in the time between publishing guidelines, (iv) FCOI declarations may have been incomplete or missing completely, and (v) reliance on voluntary reporting of FCOI by authors may have resulted in underreporting of these relationships because of the subjective decisions of individual authors [2, 28, 29].

### Limitations

We excluded guidelines if either authors or committee members were not explicitly named, limiting the scope of our analysis. Additionally, our analyses accounted for neither drugs that were recommended for second- or third-line treatment, nor the strength of evidence used to make first-line drug recommendations. We did not differentiate among the types of FCOI that the authors disclosed. Finally, we did not consider the funding source(s) of the guidelines. Our results are preliminary since our sample size of guidelines is limited.

## Conclusions

Our findings support the need for future research to measure not only the prevalence, but also underreporting of FCOI in guidelines. Our results also suggest a need for accurate and consistent disclosures. Future research is also necessary to determine whether guideline authors’ reported FCOI are associated with their drug treatment guideline recommendations.

After the Association of Scientific Medical Societies in Germany (Arbeitsgemeinschaft der Wissenschaftlichen Medizinischen Fachgesellschaften, AWMF) instituted new disclosure rules in 2010, the prevalence of guidelines with disclosures increased from 8 to 95 % in 2011. This reform requires guideline-creating groups to ensure that both their members’ declarations and the procedures used to declare, document, and the disclosures themselves are made public [26].

Physicians tend to have confidence in, and attribute value to, guidelines issued or distributed by official professional associations [30]. Therefore, we encourage professional associations including the CMA to consider developing a policy equivalent to that which was adopted by the AWMF on FCOI disclosures and we recommend that the CMA refuse to list any CPGs that do not conform to these standards.

## Additional files

Additional file 1: All clinical practice guidelines from Canadian Medical Association (CMA) Infobase as of 06 November 2013. This table lists all of the clinical practice guidelines, as well as their details, that were available through the CMA Infobase as of 06 November 2013. (PDF 1.01 mb) Additional file 2: Author-level raw data. This table provides all of the anonymized raw data for each of the authors analyzed in our study. (XLSX 43 kb) Additional file 3: Guideline-level data on recommended drugs and guideline-affiliated organizations. This table provides the recommended drugs in each guideline included in our study, the manufacturers of the brand name drugs recommended in these guidelines, the organizations that were indicated to be affiliated with each guideline, and the pharmaceutical companies with which these organizations identified relationships. (PDF 264 kb)

## Abbreviations

AWMF: Arbeitsgemeinschaft der Wissenschaftlichen Medizinischen Fachgesellschaften (Association of Scientific Medical Societies in Germany)
CADTH: Canadian Agency for Drugs and Technologies in Health
CMA: Canadian Medical Association
CPGs: Clinical Practice Guidelines
CPS: Compendium of Pharmaceuticals and Specialties
FCOI: Financial Conflicts of Interest
NGC: National Guideline Clearinghouse
