# Supplementary material for: Reporting of financial conflicts of interest in clinical practice guidelines: a case study analysis of guidelines from the Canadian Medical Association Infobase
Source: BMC Health Serv Res. 2016 Aug 15;16:383. doi: 10.1186/s12913-016-1646-5 (PMC4986411; doi:10.1186/s12913-016-1646-5)
Supplement: Additional file 1: — All clinical practice guidelines from Canadian Medical Association (CMA) Infobase as of 06 November 2013. This table lists all of the clinical practice guidelines, as well as their details, that were available through the CMA Infobase as of 06 November 2013. (PDF 1.01 mb) [file 12913_2016_1646_MOESM1_ESM.pdf]

Appendix 1: All clinical practice guidelines from Canadian Medical Association (CMA) Infobase as of 06 November 2013

Note: Highlighted guidelines were most recently reviewed or published between 01 January 2012 and 06 November 2013, inclusive.

Total	Category in CMA Infobase	Organization	Clinical Practice Guideline Title	Date most recently reviewed or published (reverse chronological order)
1	C	Canadian Network for Mood and Anxiety Treatments	Canadian Network for Mood and Anxiety Treatments (CANMAT) guidelines for the management of patients with bipolar disorder: update 2007	2006-Dec (Reviewed 2013)
2	C	Canadian Stroke Network	Canadian best practice recommendations for stroke care	2010 (Reviewed 2013)
3	L	Collège des médecins du Québec	Les niveaux de consommation d'alcool à faible risque : guide pour les médecins et les professionnels de la santé	2013
4	L	Collège des médecins du Québec	Low-risk drinking guidelines : a guide for physicians and health care professionals	2013
5	T	Canadian Hypertension Education Program	The 2012-2013 Canadian Hypertension Education Program (CHEP) guidelines for pharmacists: an update	2013
6	C	Canadian Network for Mood and Anxiety Treatments	Canadian Network for Mood and Anxiety Treatments (CANMAT) guidelines for the management of patients with bipolar disorder : consensus and controversies	2005-June (Reviewed 2013)
7	C	International Society for Bipolar Disorders; Canadian Network for Mood and Anxiety Treatments	Canadian Network for Mood and Anxiety Treatments (CANMAT) and International Society for Bipolar Disorders (ISBD) collaborative update of CANMAT guidelines for the management of patients with bipolar disorder: update 2009	2009-May (Reviewed 2013)
8	T	Canadian Paediatric Society	Trampoline use in homes and playgrounds	2007-Aug-1 (Reviewed 2013-Jan-30)
9	L	Société canadienne de pédiatrie	L'utilisation des trampolines à la maison et au terrain de jeux	2007-août-1 (Reviewed 2013-janv-30)
10	E	Société des obstétriciens et gynécologues du Canada	État actuel du dépistage prénatal non effractif du syndrome de Down, de la trisomie 18 et de la trisomie 13 au moyen d'ADN acellulaire se trouvant dans le plasma maternel	2013-janv
11	H	Société canadienne des anesthésiologistes	Guide d'exercice de l'anesthésie	2013-janv

12	L	Société des obstétriciens et gynécologues du Canada	Liste de contrôle de la sécurité chirurgicale en obstétrique-gynécologie	2013-janv
13	T	Société des obstétriciens et gynécologues du Canada	Toxoplasmose pendant la grossesse : Prévention, dépistage et traitement	2013-janv
14	R	Canadian Task Force on Preventive Health Care	Recommendations on screening for cervical cancer	2013-Jan-8
15	F	Guidelines and Protocols Advisory Committee (BC)	Follow-up of colorectal polyps or cancer	2013-Jan-16
16	C	Society of Obstetricians and Gynaecologists of Canada	Current status in non-invasive prenatal detection of down syndrome, trisomy 18, and trisomy 13 using cell-free DNA in maternal plasma	2013-Jan
17	H	Canadian Anesthesiologists' Society	Guidelines to the practice of anesthesia	2013-Jan
18	M	Canadian Association of Genitourinary Medical Oncologists	Management of small cell carcinoma of the bladder: Consensus guidelines from the Canadian Association of Genitourinary Medical Oncologists (CAGMO)	2013-Jan
19	S	Canadian Thoracic Society	Spirometry in primary care	2013-Jan
20	S	Society of Obstetricians and Gynaecologists of Canada	Surgical safety checklist in obstetrics and gynaecology	2013-Jan
21	T	Society of Obstetricians and Gynaecologists of Canada	Toxoplasmosis in pregnancy: Prevention, screening, and treatment	2013-Jan
22	R	Fondation des maladies du coeur du Canada; Réseau canadien contre les accidents cérébrovasculaires	Recommandations canadiennes pour les pratiques optimales de soins de l'AVC : Chaptire 2. prévention	2013-fév-21
23	C	Réseau canadien contre les accidents cérébrovasculaires	Canadian Best Practice Recommendations for Stroke Care	2013-fév
24	P	Programme éducatif canadien sur l'hypertension	PECH recommandations 2013 pour la prise en charge de l'hypertension artérielle	2013-fév
25	0-9	Canadian Cardiovascular Society	2012 update of the Canadian Cardiovascular Society guidelines for the diagnosis and treatment of dyslipidemia for the prevention of cardiovascular disease in the adult	2013-Feb

26	C	Canadian Cardiovascular Society	Canadian Cardiovascular Society guidelines on the use of cardiac resynchronization therapy: Evidence and patient selection	2013-Feb
27	C	Canadian Network for Mood and Anxiety Treatments	Canadian Network for Mood and Anxiety Treatments (CANMAT) and International Society for Bipolar Disorders (ISBD) collaborative update of CANMAT guidelines for the management of patients with bipolar disorder: update 2013	2013-Feb
28	C	Canadian Hypertension Education Program	CHEP 2013 recommendations for management of hypertension	2013-Feb
29	T	Canadian Cardiovascular Society	The 2012 Canadian Cardiovascular Society heart failure management guidelines update: Focus on acute and chronic heart failure	2013-Feb
30	C	Société des obstétriciens et gynécologues du Canada	Chimiothérapie anticancéreuse et grossesse	2013-mars
31	D	Comité consultatif de la médicine tropicale et de la médecine des voyages (CCMTMV)	Déclaration sur la fièvre jaune à l’intention des voyageurs	2013-mars
32	R	Fondation des maladies du coeur du Canada; Réseau canadien contre les accidents cérébrovasculaires	Recommandations canadiennes pour les pratiques optimales de soins de l'AVC : Chapitre 7. humeur et cognition post-AVC	2013-mars
33	C	Guidelines and Protocols Advisory Committee (BC)	Colorectal screening for cancer prevention in asymptomatic patients	2013-Mar-1
34	A	Registered Nurses' Association of Ontario	Assessment and management of foot ulcers for people with diabetes	2013-Mar
35	B	Cancer Care Ontario's Program in Evidence-based Care	Bortezomib in multiple myeloma and lymphoma	2013-Mar
36	C	Heart and Stroke Foundation of Canada; Canadian Stroke Network	Canadian best practice recommendations for stroke care : chapter 7. mood and cognition in patients following stroke	2013-Mar
37	C	Society of Obstetricians and Gynaecologists of Canada	Cancer chemotherapy and pregnancy	2013-Mar
38	H	Ontario Association of Medical Laboratories	Guidelines for ordering urine testing for drugs-of-abuse: Targeted and screening tests	2013-Mar

39	P	Alberta Health Services - Cancer Care	Proton beam radiation therapy	2013-Mar
40	R	Canadian Agency for Drugs and Technologies in Health	Recommendations for antithrombotic agents for the prevention of stroke and systemic embolism in patients with atrial fibrillation	2013-Mar
41	S	Committee to Advise on Tropical Medicine and Travel (CATMAT)	Statement for travellers and yellow fever	2013-Mar
42	P	Guidelines and Protocols Advisory Committee (BC)	Problem drinking	2011-Apr (Reviewed 2013-Apr)
43	L	Société canadienne de pédiatrie	Le sevrage de l’allaitement	2013-avril
44	C	Canadian Urological Association	CUA-CUOG guidelines for the management of castration-resistant prostate cancer (CRPC): 2013 update	2013-Aug
45	I	Society of Obstetricians and Gynaecologists of Canada	Intrauterine growth restriction: screening, diagnosis, and management	2013-Aug
46	M	Canadian Kidney Cancer Forum	Management of advanced kidney cancer: Canadian Kidney Cancer Forum 2013 consensus update	2013-Aug
47	C	Canadian Cardiovascular Society; Canadian Heart Rhythm Society	Canadian Cardiovascular Society/Canadian Heart Rhythm Society joint position statement on the use of remote monitoring for cardiovascular implantable electronic device follow-up	2013-Apr
48	C	Canadian Diabetes Association	Canadian Diabetes Association 2013 clinical practice guidelines for the prevention and management of diabetes in Canada	2013-Apr
49	C	Canadian Diabetes Association	Canadian Diabetes Association 2013 clinical practice guidelines for the prevention and management of diabetes in Canada : Definition, classification and diagnosis of diabetes, prediabetes and metabolic syndrome	2013-Apr
50	C	Canadian Diabetes Association	Canadian Diabetes Association 2013 clinical practice guidelines for the prevention and management of diabetes in Canada : Diabetes and pregnancy	2013-Apr

51	C	Canadian Diabetes Association	Canadian Diabetes Association 2013 clinical practice guidelines for the prevention and management of diabetes in Canada : Diabetes in the elderly	2013-Apr
52	C	Canadian Diabetes Association	Canadian Diabetes Association 2013 clinical practice guidelines for the prevention and management of diabetes in Canada : Hyperglycemic emergencies in adults	2013-Apr
53	C	Canadian Diabetes Association	Canadian Diabetes Association 2013 clinical practice guidelines for the prevention and management of diabetes in Canada : Hypoglycemia	2013-Apr
54	C	Canadian Diabetes Association	Canadian Diabetes Association 2013 clinical practice guidelines for the prevention and management of diabetes in Canada : Introduction	2013-Apr
55	C	Canadian Diabetes Association	Canadian Diabetes Association 2013 clinical practice guidelines for the prevention and management of diabetes in Canada : Methods	2013-Apr
56	C	Canadian Diabetes Association	Canadian Diabetes Association 2013 clinical practice guidelines for the prevention and management of diabetes in Canada : Monitoring glycemic control	2013-Apr
57	C	Canadian Diabetes Association	Canadian Diabetes Association 2013 clinical practice guidelines for the prevention and management of diabetes in Canada : Nutrition therapy	2013-Apr
58	C	Canadian Diabetes Association	Canadian Diabetes Association 2013 clinical practice guidelines for the prevention and management of diabetes in Canada : Organization of diabetes care	2013-Apr
59	C	Canadian Diabetes Association	Canadian Diabetes Association 2013 clinical practice guidelines for the prevention and management of diabetes in Canada : Pharmacologic management of type 2 diabetes	2013-Apr

60	C	Canadian Diabetes Association	Canadian Diabetes Association 2013 clinical practice guidelines for the prevention and management of diabetes in Canada : Pharmacotherapy in type 1 diabetes	2013-Apr
61	C	Canadian Diabetes Association	Canadian Diabetes Association 2013 clinical practice guidelines for the prevention and management of diabetes in Canada : Physical activity and diabetes	2013-Apr
62	C	Canadian Diabetes Association	Canadian Diabetes Association 2013 clinical practice guidelines for the prevention and management of diabetes in Canada : Reducing the risk of developing diabetes	2013-Apr
63	C	Canadian Diabetes Association	Canadian Diabetes Association 2013 clinical practice guidelines for the prevention and management of diabetes in Canada : Screening for type 1 and type 2 diabetes	2013-Apr
64	C	Canadian Diabetes Association	Canadian Diabetes Association 2013 clinical practice guidelines for the prevention and management of diabetes in Canada : Self-management education	2013-Apr
65	C	Canadian Diabetes Association	Canadian Diabetes Association 2013 clinical practice guidelines for the prevention and management of diabetes in Canada : Targets for glycemic control	2013-Apr
66	C	Canadian Diabetes Association	Canadian Diabetes Association 2013 clinical practice guidelines for the prevention and management of diabetes in Canada : Type 2 diabetes in aboriginal peoples	2013-Apr
67	H	Public Health Ontario	Guidelines for testing and treatment of gonorrhea in Ontario	2013-Apr
68	T	Society of Obstetricians and Gynaecologists of Canada	The role of adjuvant therapy in endometrial cancer	2013-Apr
69	W	Canadian Paediatric Society	Weaning from the breast	2013-Apr
70	S	College of Physicians and Surgeons of Ontario	Sleep medicine - 3rd Edition, September 2010	2010-Sept (Reviewed 2013-May)

71	L	Société canadienne de pédiatrie	La prise en charge du patient d'âge pédiatrique victime d'un traumatisme crânien aigu	2013-mai-3
72	R	Fondation des maladies du coeur du Canada; Réseau canadien contre les accidents cérébrovasculaires	Recommandations canadiennes pour les pratiques optimales de soins de l'AVC : chaptire 3. soins de l'AVC en phase hyperaiguë	2013-mai
73	R	Fondation des maladies du coeur du Canada	Recommandations canadiennes pour les pratiques optimales de soins de l'AVC: Chapitre 4. soins du patient avec AVC aigu hospitalisé	2013-mai
74	S	Société des obstétriciens et gynécologues du Canada	Saignements utérins anormaux chez les femmes préménopausées	2013-mai
75	M	Canadian Paediatric Society	Management of the paediatric patient with acute head trauma	2013-May-3
76	R	Canadian Task Force on Preventive Health Care	Recommendations on screening for depression in adults	2013-May-13
77	A	Society of Obstetricians and Gynaecologists of Canada	Abnormal uterine bleeding in pre-menopausal women	2013-May
78	A	Perinatal Services BC	Antidepressant use during pregnancy: considerations for the newborn exposed to SSRIs/SNRIs	2013-May
79	C	Heart and Stroke Foundation of Canada; Canadian Stroke Network	Canadian best practice recommendations for stroke care : chapter 3. hyperacute stroke care	2013-May
80	C	Heart and Stroke Foundation of Canada	Canadian best practice recommendations for stroke care : chapter 4. acute inpatient stroke care	2013-May
81	R	Cancer Care Ontario's Program in Evidence-based Care	Routine HPV testing in head and neck squamous cell carcinoma	2013-May
82	T	Canadian Hypertension Education Program	The 2013 Canadian Hypertension Education Program Recommendations for blood pressure measurement, diagnosis, assessment of risk, prevention, and treatment of hypertension	2013-May
83	A	Guidelines and Protocols Advisory Committee (BC)	Antinuclear antibody (ANA) testing for connective tissue disease	2013-June
84	L	Collège des médecins du Québec; Institut national de santé publique du Québec	Le dépistage du cancer de la prostate - mise à jour 2013	2013-juin

85	E	Cancer Care Ontario's Program in Evidence-based Care	Evidence-based guideline recommendations on low-dose rate brachytherapy in patients with low- or intermediate-risk prostate cancer	2013-June
86	B	British Columbia Centre for Excellence in HIV/AIDS	British Columbia guidelines for the care of HIV positive pregnant women and interventions to reduce perinatal transmission guidelines	2013-July
87	C	Heart and Stroke Foundation of Canada	Canadian best practice recommendations for stroke care : chapter 5. stroke rehabilitation	2013-July
88	C	Public Health Agency of Canada	Canadian guidelines on sexually transmitted infections : Gonococcal infections	2013-July
89	O	Canadian Agency for Drugs and Technologies in Health	Optimal use recommendations for second- and third-line therapy for patients with type 2 diabetes	2013-July
90	R	Fondation des maladies du coeur du Canada	Recommandations canadiennes pour les pratiques optimales de soins de l'AVC : Chapitre 5. réadaptation post-AVC	2013-juill
91	P	Canadian Kidney Cancer Forum	Prise en charge du cancer du rein de stade avancé : Mise à jour 2013 des lignes directrices consensuelles du Forum canadien sur le cancer du rein	2013-août
92	R	Société des obstétriciens et gynécologues du Canada	Retard de croissance intra-utérin : Dépistage, diagnostic et prise en charge	2013-août
93	C	Heart and Stroke Foundation of Canada; Canadian Stroke Network	Canadian best practice recommendations for stroke care : chapter 8. telestroke	2013-Sept
94	C	Canadian Headache Society	Canadian Headache Society guideline : acute drug therapy for migraine headache	2013-Sept
95	H	Ontario Neurotrauma Foundation	Guidelines for concussion/mild traumatic brain injury & persistent symptoms: second edition	2013-Sept
96	I	Society of Obstetricians and Gynaecologists of Canada	Induction of labour at term	2013-Sept
97	R	Groupe d'étude canadien sur les soins de santé préventifs	Recommandations pour le dépistage de l’hypertension chez les adultes canadiens	2013-sept
98	R	Canadian Task Force on Preventive Health Care	Recommendations on screening for high blood pressure in Canadian adults	2013-Sept

99	S	Cancer Care Ontario's Program in Evidence-based Care	Screening high-risk populations for lung cancer	2013-Sept
100	S	Société canadienne de pédiatrie	Soutenir la santé mentale des enfants et des adolescents de parents qui se séparent	2013-sept
101	S	Canadian Paediatric Society	Supporting the mental health of children and youth of separating parents	2013-Sept
102	C	Canadian Association of Gastroenterology	Canadian Association of Gastroenterology position statement: Hip fracture and proton pump inhibitor therapy – a 2013 update	2013-Oct
103	C	Canadian Cardiovascular Society	Canadian Cardiovascular Society guidelines on the use of cardiac resynchronization therapy: Implementation	2013-Nov
104	C	Canadian Cardiovascular Society	Canadian Cardiovascular Society position statement on radiation exposure from cardiac imaging and interventional procedures	2013-Nov
105	0-9	Canadian Pain Society	2012 Canadian Guidelines for the diagnosis and management of fibromyalgia syndrome	2012
106	A	Alberta Health and Wellness	Alberta treatment guidelines for sexually transmitted infections (STI) in adolescents and adults 2012	2012
107	C	Public Health Agency of Canada	Canadian immunization guide	2012
108	C	Canadian Action Network for the Advancement, Dissemination and Adoption of Practice-informed Tobacco Treatment (CAN-ADAPTT)	Canadian smoking cessation clinical practice guideline	2012
109	L	Collège des médecins du Québec; Agence de la santé et de services sociaux de Montréal	L'évaluation médicale périodique de l'adulte	2012
110	L	Réseau d’action canadien pour l’avancement, la dissémination et l’adoption de pratiques en matière de traitement du tabagisme fondées sur l’expérience clinique (CAN-ADAPTT)	Lignes directrices canadiennes de pratique clinique sur le renoncement au tabagisme	2012
111	R	Canadian Task Force on Preventive Health Care	Recommendations on screening for high blood pressure in Canadian adults	2012

112	T	Association of Medical Microbiology and Infectious Disease Canada	The use of antiviral drugs for influenza: Guidance for practitioners 2012/2013	2012
113	L	Société canadienne de pédiatrie	Les recommandations sur l'usage des sucettes	2003-oct (Reviewed 2012-janv)
114	O	Cancer Care Ontario's Program in Evidence-based Care	Optimal therapy for patients diagnosed with multiple myeloma and the role of high-dose chemotherapy and stem cell support	2003-Oct (Reviewed 2012-Jan)
115	R	Canadian Paediatric Society	Recommendations for the use of pacifiers	2003-Oct (Reviewed 2012-Jan)
116	L	Société canadienne de pédiatrie	La question des soins aux adolescents hospitalisés	2008-janv (Reviewed 2012-janv)
117	L	Société canadienne de pédiatrie	La réduction des méfaits : Une démarche pour réduire les comportements à risque des adolescents en matière de santé	2008-janv (Reviewed 2012-janv)
118	I	Canadian Paediatric Society	Issues of care for hospitalized youth	2008-Jan (Reviewed 2012-Jan)
119	L	Société canadienne de pédiatrie	La prise en charge de l'énurésie nocturne primaire	2010-fév (Reviewed 2012-janv)
120	M	Canadian Paediatric Society	Management of primary nocturnal enuresis	2010-Feb (Reviewed 2012-Jan)
121	L	Société canadienne de pédiatrie	Les répercussions de la fréquentation des services de garde sur la santé des enfants: Partie B : Les blessures et les infections	2009-janv (Reviewed 2012-janv)
122	L	Société canadienne de pédiatrie	Les blessures par piqûre d'aiguille dans un lieu public	2008 (Reviewed 2012-janv)
123	N	Canadian Paediatric Society	Needle stick injuries in the community	2008 (Reviewed 2012-Jan)
124	M	Alberta Health Services - Cancer Care	Magnetic resonance imaging for breast cancer screening, pre-operative assessment, and follow-up	2010-Oct (Reviewed 2012-Jan)
125	D	Société canadienne de pédiatrie	Des considérations particulières pour la supervision médicale des enfants et des adolescents en famille d'accueil	2008-fév (Reviewed 2012-janv)
126	S	Canadian Paediatric Society	Special considerations for the health supervision of children and youth in foster care	2008-Feb (Reviewed 2012-Jan)
127	L	Société canadienne de pédiatrie	Le dépistage du VIH pendant la grossesse	2008-mars (Reviewed 2012-janv)
128	T	Canadian Paediatric Society	Testing for HIV infection in pregnancy	2008-Mar (Reviewed 2012-Jan)
129	S	Manitoba Health and Healthy Living	Sexually transmitted infections treatment guidelines	2008-Apr (Reviewed 2012-Jan)

130	L	Société canadienne de pédiatrie	Le contrôle des infections au cabinet du pédiatre	2008-juin (Reviewed 2012-janv)
131	L	Société canadienne de pédiatrie	La transmission verticale du virus de l'hépatite C : les connaissances et les enjeux courants	2008-juill (Reviewed 2012-janv)
132	V	Canadian Paediatric Society	Vertical transmission of the hepatitis C virus : current knowledge and issues	2008-July (Reviewed 2012-Jan)
133	I	Canadian Paediatric Society	Infection control in paediatric office settings	2008-June (Reviewed 2012-Jan)
134	T	Alberta Health Services - Cancer Care	Testicular germ cell tumours	2005-Apr (Reviewed 2012-Jan)
135	L	Société canadienne de pédiatrie	L'homéopathie dans la population pédiatrique	2005-mars (Reviewed 2012-janv)
136	L	Société canadienne de pédiatrie	La mesure de la température en pédiatrie	2005-mars (Reviewed 2012-janv)
137	T	Canadian Paediatric Society	Temperature measurement in paediatrics	2005-Mar (Reviewed 2012-Jan)
138	A	Canadian Paediatric Society	Antimicrobial products in the home : the evolving problem of antibiotic resistance	2006-Mar (Reviewed 2012-Jan)
139	L	Société canadienne de pédiatrie	La grossesse à l'adolescence	2006-avril (Reviewed 2012-janv)
140	A	Canadian Paediatric Society	Adolescent pregnancy	2006-Apr (Reviewed 2012-Jan)
141	L	Société canadienne de pédiatrie	L'adoption interraciale	2006-sept (Reviewed 2012-janv)
142	T	Canadian Paediatric Society	Transracial adoption	2006-Sept (Reviewed 2012-Jan)
143	L	Société canadienne de pédiatrie	La paralysie périnatale du plexus brachial	2006-fév (Reviewed 2012-janv)
144	P	Canadian Paediatric Society	Perinatal brachial plexus palsy	2006-Feb (Reviewed 2012-Jan)
145	L	Société canadienne de pédiatrie	Les soins aux adolescents ayant une maladie chronique	2006-janv (Reviewed 2012-janv)
146	C	Canadian Paediatric Society	Care of adolescents with chronic conditions	2006-Jan (Reviewed 2012-Jan)
147	L	Société canadienne de pédiatrie	L’orientation sexuelle des adolescents	2008-sept (Reviewed 2012-janv)
148	U	Société canadienne de pédiatrie	Une morsure dans la salle de jeu : la prise en charge des morsures humaines en milieu de garde	2008-sept (Reviewed 2012-janv)
149	A	Canadian Paediatric Society	A bite in the playroom: managing human bites in child care settings	2008-Sept (Reviewed 2012-Jan)
150	A	Canadian Paediatric Society	Adolescent sexual orientation	2008-Sept (Reviewed 2012-Jan)
151	L	Société canadienne de pédiatrie	Les infestations par les poux de tête : Une mise à jour clinique	2008-oct (Reviewed 2012-janv)
152	M	Alberta Health Services - Cancer Care	Management of febrile neutropenia in adult cancer patients	2008-Nov (Reviewed 2012-Jan)

153	L	Société canadienne de pédiatrie	Les répercussions de la fréquentation des services de garde sur la santé des enfants: Partie A : Les tendances canadiennes des issues du comportement et du développement sur les enfants en service de garde	2008-déc (Reviewed 2012-janv)
154	L	Société canadienne de pédiatrie	Les voyages en avion et la santé des enfants	2007-janv (Reviewed 2012-janv)
155	A	Canadian Paediatric Society	Air travel and children's health issues	2007-Jan (Reviewed 2012-Jan)
156	L	Société canadienne de pédiatrie	La prévention du syndrome de rubéole congénitale	2007-fév (Reviewed 2012-janv)
157	P	Canadian Paediatric Society	Prevention of congenital rubella syndrome	2007-Feb (Reviewed 2012-Jan)
158	L	Société canadienne de pédiatrie	Les troubles envahissants du développement : aucune relation causale avec les vaccins	2007-juin (Reviewed 2012-janv)
159	A	Canadian Paediatric Society	Autistic spectrum disorder: no causal relationship with vaccines	2007-June (Reviewed 2012-Jan)
160	L	Société canadienne de pédiatrie	La transition des jeunes ayant des besoins particuliers vers les soins pour adultes	2007-nov (Reviewed 2012-janv)
161	L	Société canadienne de pédiatrie	Les jeunes et les armes à feu au Canada	2005-oct (Reviewed 2012-janv)
162	U	Canadian Paediatric Society	Use of anencephalic newborns as organ donors	2005-July (Reviewed 2012-Jan)
163	L	Société canadienne de pédiatrie	Le recours aux nouveau-nés anencéphales comme donneurs d'organes	2005-juill (Reviewed 2012-janv)
164	L	Société canadienne de pédiatrie	Les normes de santé pour les jeunes en établissement de détention	2005-mai (Reviewed 2012-janv)
165	Y	Canadian Paediatric Society	Youth and firearms in Canada	2005-Oct (Reviewed 2012-Jan)
166	T	Canadian Paediatric Society	Transition to adult care for youth with special health care needs	2007-Nov (Reviewed 2012-Jan)
167	L	Société canadienne de pédiatrie	Les politiques d'exclusion des garderies et des écoles en cas de varicelle : une démarche rationnelle	1999-mai (Reviewed 2012-janv)
168	C	Canadian Paediatric Society	Chiropractic care for children : controversies and issues	2002-Feb (Reviewed 2012-Jan)
169	D	Société canadienne de pédiatrie	Des directives pour les professionels de la santé qui soutiennent des familles après un décès périnatal	2001-sept (Reviewed 2012-janv)

170	H	Canadian Paediatric Society	Guidelines for health care professionals supporting families experiencing a perinatal loss	2001-Sept (Reviewed 2012-Jan)
171	S	Canadian Paediatric Society	School and daycare exclusion policies for chickenpox: a rational approach	1999-May (Reviewed 2012-Jan)
172	L	Société canadienne de pédiatrie	Les soins chiropratiques aux enfants : des controverses et des points litigieux	2002-fév (Reviewed 2012-janv)
173	L	Société canadienne de pédiatrie	L'apprentissage de la propreté : des conseils axés sur l'enfant	2000-sept (Reviewed 2012-janv)
174	T	Canadian Paediatric Society	Toilet learning : anticipatory guidance with a child-oriented approach	2000-Sept (Reviewed 2012-Jan)
175	L	Société canadienne de pédiatrie	Les régimes à l'adolescence	2004-sept (Reviewed 2012-janv)
176	D	Canadian Paediatric Society	Dieting in adolescence	2004-Sept (Reviewed 2012-Jan)
177	D	Société canadienne de pédiatrie	Des recommandations pour créer des environnements de sommeil sécuritaires pour les nourrissons et les enfants	2004-nov (Reviewed 2012-janv)
178	R	Canadian Paediatric Society	Recommendations for safe sleeping environments for infants and children	2004-Nov (Reviewed 2012-Jan)
179	D	Société canadienne de pédiatrie	Des lignes directrices pour le dépistage des nouveau-nés vulnérables à l'hypoglycémie	2004-déc (Reviewed 2012-janv)
180	S	Canadian Paediatric Society	Screening guidelines for newborns at risk for low blood glucose	2004-Dec (Reviewed 2012-Jan)
181	A	Canadian Paediatric Society	Assessment of babies for car seat safety before hospital discharge	2000-Jan (Reviewed 2012-Jan)
182	E	Société canadienne de pédiatrie	Évaluation de la sécurité des bébés dans leur siège d'auto avant leur congé de l'hôpital	2000-janv (Reviewed 2012-janv)
183	L	Société canadienne de pédiatrie	L'administration systématique de vitamine K aux nouveau-nés	1997-nov (Reviewed 2012-janv)
184	R	Canadian Paediatric Society	Routine administration of vitamin K to newborns	1997-Nov (Reviewed 2012-Jan)
185	L	Société canadienne de pédiatrie	La prévention des blessures en ski et en planche à neige	2012-janv
186	L	Société canadienne de pédiatrie	L’évaluation et la prise en charge des enfants et des adolescents victimes d’une commotion liée à un sport	2012-janv
187	R	Société des obstétriciens et gynécologues du Canada	Report de la grossesse	2012-janv

188	C	Canadian Association of Gastroenterology	Canadian Association of Gastroenterology consensus guidelines on safety and quality indicators in endoscopy	2012-Jan
189	C	Guidelines and Protocols Advisory Committee (BC)	Cobalamin (vitamin B12) deficiency - Investigation & management	2012-Jan
190	D	Society of Obstetricians and Gynaecologists of Canada	Delayed child-bearing	2012-Jan
191	E	Canadian Paediatric Society	Evaluation and management of children and adolescents with sports-related concussion	2012-Jan
192	F	Guidelines and Protocols Advisory Committee (BC)	Folate deficiency - Investigation & management	2012-Jan
193	M	Saskatchewan Cancer Agency	Myelodysplastic syndrome treatment guidelines	2012-Jan
194	S	Canadian Paediatric Society	Skiing and snowboarding injury prevention	2012-Jan
195	S	Alberta Health Services - Cancer Care	Superior sulcus (pancoast) tumours	2012-Jan
196	V	Guidelines and Protocols Advisory Committee (BC)	Viral hepatitis testing	2012-Jan
197	L	Société canadienne de pédiatrie	La prise en charge courante de l'infection au virus de l'herpès simplex chez les femmes enceintes et leur nouveau-né	2006-août (Reviewed 2012-fév)
198	P	Alberta Health Services - Cancer Care	Penile cancer	2011-Jan (Reviewed 2012-Feb)
199	P	Cancer Care Ontario's Program in Evidence-based Care	PET imaging in head and neck cancer	2009-Jan (Reviewed 2012-Feb)
200	H	Ontario Association of Medical Laboratories	Guidelines for the use of serum tests for iron deficiency	1995-Apr (Reviewed 2012-Feb)
201	I	Société canadienne de pédiatrie	Interdire aux enfants et aux adolescents de moins de 18 ans l'accès aux établissements commerciaux de bronzage	2012-fév
202	L	Société canadienne de pédiatrie	La prévention de l'étouffement et de la suffocation chez les enfants	2012-fév
203	L	Ordre des psychologues du Québec; Collège des médecins du Québec	Les troubles du spectre de l'autisme - l'évaluation clinique	2012-fév
204	R	Programme éducatif canadien sur l'hypertension	Recommandation 2012 du PECH - Prise en charge de I'hypertension artérielle	2012-fév
205	F	Cancer Care Ontario's Program in Evidence-based Care	Follow-up care, surveillance protocol, and secondary prevention measures for survivors of colorectal cancer: Guideline recommendations	2012-Feb-3

206	0-9	Canadian Hypertension Education Program	2012 CHEP recommendations for management of hypertension	2012-Feb
207	B	Canadian Paediatric Society	Banning children and youth under the age of 18 years from commercial tanning facilities	2012-Feb
208	C	Canadian Headache Society	Canadian Headache Society guideline for migraine prophylaxis	2012-Feb
209	C	Canadian Headache Society	Canadian Headache Society guideline for migraine prophylaxis : Introduction to the guideline, and general principles of migraine prophylaxis - Section I	2012-Feb
210	C	Canadian Headache Society	Canadian Headache Society guideline for migraine prophylaxis: Systematic review: Medications for migraine prophylaxis - Section II	2012-Feb
211	C	Canadian Headache Society	Canadian Headache Society guideline for migraine prophylaxis: Treatment strategies: pharmacological prophylaxis - Section III	2012-Feb
212	P	Canadian Paediatric Society	Preventing choking and suffocation in children	2012-Feb
213	L	Société canadienne de pédiatrie	Le syndrome d'alcoolisme foetal	2002-mars (Reviewed 2012-mars)
214	F	Canadian Paediatric Society	Fetal alcohol syndrome	2002-Mar (Reviewed 2012-Mar)
215	L	Association dentaire canadienne	L’utilisation des fluorures pour la prévention des caries	2003-mars (Reviewed 2012-mars)
216	U	Canadian Dental Association	Use of fluorides in caries prevention	2003-Mar (Reviewed 2012-Mar)
217	L	Société canadienne de pédiatrie	Les suppléments de vitamine D: recommandations pour les mères et leur nourrisson au Canada	2007-sept (Reviewed 2012-mars)
218	V	Canadian Paediatric Society	Vitamin D supplementation: recommendations for Canadian mothers and infants	2007-Sept (Reviewed 2012-Mar)
219	D	Société canadienne de pédiatrie	Des recommandations pour le traitement néonatal par surfactant exogène: Addenda 2012	2005-fév (Reviewed 2012-mars)
220	R	Canadian Paediatric Society	Recommendations for neonatal surfactant therapy: Addendum 2012	2005-Feb (Reviewed 2012-Mar)
221	L	Société canadienne de pédiatrie	La réduction du risque de diabète de type 2 chez les enfants autochtones du Canada	2005-janv (Reviewed 2012-mars)

222	R	Canadian Paediatric Society	Risk reduction for type 2 diabetes in Aboriginal children in Canada	2005-Jan (Reviewed 2012-Mar)
223	A	Alberta Health Services - Cancer Care	Adjuvant interferon for malignant melanoma	2007-May (Reviewed 2012-Mar)
224	O	Alberta Health Services - Cancer Care	Optimal excision margins for primary cutaneous melanoma	2011-Feb (Reviewed 2012-Mar)
225	R	Alberta Health Services - Cancer Care	Regional node dissection in primary cutaneous melanoma	2011-Feb (Reviewed 2012-Mar)
226	P	Alberta Health Services - Cancer Care	Preoperative and pretreatment investigations for malignant melanoma	2009-June (Reviewed 2012-Mar)
227	A	Alberta Health Services - Cancer Care	Adjuvant radiation for malignant melanoma	2008-May (Reviewed 2012-Mar)
228	B	Alberta Health Services - Cancer Care	Biopsy of a suspicious pigmented lesion	2008-May (Reviewed 2012-Mar)
229	D	Société canadienne de physiologie de l'exercice	Directives canadiennes en matière d'activité physique pour la petite enfance (enfants âgés de 0 à 4 ans)	2012-mars
230	D	Société canadienne de physiologie de l'exercice	Directives canadiennes en matière de comportement sédentaire pour la petite enfance (enfants âgés de 0 à 4 ans)	2012-mars
231	F	Société des obstétriciens et gynécologues du Canada	Facteurs génétiques à prendre en considération dans le cadre de l'examen gynécologique annuel	2012-mars
232	L	Société canadienne de pédiatrie	Les aspects psychosociaux de l'obésité chez les enfants et les adolescents	2012-mars
233	P	Société des obstétriciens et gynécologues du Canada	Prise en charge de l'infection à la varicelle pendant la grossesse	2012-mars
234	U	Société canadienne de pédiatrie	Une vie saine et active : des directives en matière d'activité physique chez les enfants et les adolescents	2012-mars
235	B	Saskatchewan Cancer Agency	Breast cancer treatment guidelines	2012-Mar
236	C	Canadian Cardiovascular Society	Canadian Cardiovascular Society focused position statement update on assessment of the cardiac patient for fitness to drive: Fitness following left ventricular assist device implantation	2012-Mar

237	C	Canadian Cardiovascular Society; Canadian Anesthesiologists' Society; Canadian Heart Rhythm Society	Canadian Cardiovascular Society/Canadian Anesthesiologists' Society/Canadian Heart Rhythm Society joint position statement on the perioperative management of patients with implanted pacemakers, defibrillators, and neurostimulating devices	2012-Mar
238	C	Canadian Consensus Group on the Management of Tic Disorder	Canadian guidelines for the evidence-based treatment of tic disorders: Behavioural therapy, deep brain stimulation, and transcranial magnetic stimulation	2012-Mar
239	C	Canadian Consensus Group on the Management of Tic Disorder	Canadian guidelines for the evidence-based treatment of tic disorders: Pharmacotherapy	2012-Mar
240	C	Canadian Society for Exercise Physiology	Canadian physical activity guidelines for the early years (aged 0–4 year)	2012-Mar
241	C	Canadian Society for Exercise Physiology	Canadian sedentary behaviour guidelines for the early years (aged 0–4 years)	2012-Mar
242	F	Canadian Cardiovascular Society	Focused 2012 update of the Canadian Cardiovascular Society atrial fibrillation guidelines: Recommendations for stroke prevention and rate / rhythm control	2012-Mar
243	H	Society of Obstetricians and Gynaecologists of Canada	Genetic considerations for a woman’s annual gynaecological examination	2012-Mar
244	M	Canadian Cardiovascular Society; Canadian Pain Society	Management of patients with refractory angina: Canadian Cardiovascular Society/Canadian Pain Society joint guidelines	2012-Mar
245	M	Society of Obstetricians and Gynaecologists of Canada	Management of varicella infection (Chickenpox) in pregnancy	2012-Mar
246	P	Canadian Paediatric Society	Psychosocial aspects of child and adolescent obesity	2012-Mar
247	S	Alberta Health Services - Cancer Care	Systemic therapy for unresectable stage III or metastatic cutaneous melanoma	2012-Mar
248	T	Cancer Care Ontario's Program in Evidence-based Care	The role of endolaryngeal surgery (with or without laser) versus radiotherapy in the management of early (T1) glottic cancer	2012-Mar

249	A	Alberta Health Services - Cancer Care	Adjuvant radiation therapy for invasive breast cancer	2008-June (Reviewed 2012-Apr)
250	C	Alberta Health Services - Cancer Care	Cancer of the uterine cervix	2009-Oct (Reviewed 2012-Apr)
251	E	Alberta Health Services - Cancer Care	Endometrial cancer	2009-Nov (Reviewed 2012-Apr)
252	O	Alberta Health Services - Cancer Care	Ovarian germ cell tumours	2010-June (Reviewed 2012-Apr)
253	E	Alberta Health Services - Cancer Care	Epithelial ovarian, fallopian tube and primary peritoneal cancer	2010-July (Reviewed 2012-Apr)
254	A	Société des obstétriciens et gynécologues du Canada	Antibioprophylaxie dans le cadre d’interventions gynécologiques	2012-avril
255	H	Société canadienne d'opthalmologie	Guide de pratique clinique factuelle de la Société canadienne d’ophtalmologie pour la gestion de la rétinopathie diabétique	2012-avril
256	A	Canadian Thoracic Society	Alpha-1 antitrypsin deficiency targeted testing and augmentation therapy: A Canadian Thoracic Society clinical practice guideline	2012-Apr
257	A	Society of Obstetricians and Gynaecologists of Canada	Antibiotic prophylaxis in gynaecologic procedures	2012-Apr
258	C	Canadian Ophthalmological Society	Canadian Ophthalmological Society evidence-based clinical practice guidelines for the management of diabetic retinopathy	2012-Apr
259	C	Canadian Thoracic Society	Canadian Thoracic Society 2012 guideline update: Diagnosis and management of asthma in preschoolers, children and adults	2012-Apr
260	M	Cardiometabolic Risk Working Group	Managing cardiometabolic risk in primary care - Summary of the 2011 consensus statement	2012-Apr
261	N	Alberta Health Services - Cancer Care	Non-small cell lung cancer - stage III	2012-Apr
262	R	Alberta Health Services - Cancer Care	Renal cell carcinoma	2005-Apr (Reviewed 2012-May)
263	L	Alberta Health Services - Cancer Care	Low-grade astrocytomas and oligodendrogliomas	2008-July (Reviewed 2012-May)
264	B	Perinatal Services BC	Breastfeeding healthy term infants	2012-May
265	C	Cancer Care Ontario's Program in Evidence-based Care	Cervical screening: A guideline for clinical practice in Ontario	2012-May
266	C	Society of Obstetricians and Gynaecologists of Canada	Counselling considerations for prenatal genetic screening	2012-May

267	E	Canadian Pain Society	Evidence-based guideline for neuropathic pain interventional treatments: Spinal cord stimulation, intravenous infusions, epidural injections and nerve blocks - Abstract only	2012-May
268	H	Canadian Paediatric Society	Gambling in children and adolescents	2012-May
269	L	Cancer Care Ontario's Program in Evidence-based Care	Lenalidomide in multiple myeloma	2012-May
270	M	Society of Obstetricians and Gynaecologists of Canada	Management of group B streptococcal bacteriuria in pregnancy	2012-May
271	M	Canadian Paediatric Society	Managing the paediatric patient with an acute asthma exacerbation	2012-May
272	R	Cancer Care Ontario's Program in Evidence-based Care	Referral of patients with suspected colorectal cancer by family physicians and other primary care providers	2012-May
273	R	Cancer Care Ontario's Program in Evidence-based Care	Risk reduction of prostate cancer with drugs or nutritional supplements	2012-May
274	T	Canadian Hypertension Education Program	The 2012 Canadian Hypertension Education Program recommendations for the management of hypertension: Blood pressure measurement, diagnosis, assessment of risk, and therapy	2012-May
275	F	Société des obstétriciens et gynécologues du Canada	Facteurs liés au counseling à prendre en considération dans le cadre du dépistage génétique prénatal	2012-mai
276	L	Société canadienne de pédiatrie	La prise en charge du patient pédiatrique présentant une exacerbation aiguë de l’asthme	2012-mai
277	L	Société canadienne de pédiatrie	Les jeux de hasard chez les enfants et les adolescents	2012-mai
278	P	Société des obstétriciens et gynécologues du Canada	Prise en charge de la bactériurie à streptocoques du groupe B pendant la grossesse	2012-mai
279	L	Société canadienne de pédiatrie	L'Initiative Amis des bébés : protéger promouvoir et soutenir l'allaitement	2012-juin
280	L	Société canadienne de pédiatrie	La prévention des blessures dans les terrains de jeux	2012-juin
281	L	Société des obstétriciens et gynécologues du Canada	Lignes directrices canadiennes en matière de planification de la grossesse en présence du VIH	2012-juin

282	A	Alberta Health Services - Cancer Care	Adjuvant radiation therapy for ductal carcinoma in situ	2008-June (Reviewed 2012-June)
283	C	Canadian Agency for Drugs and Technologies in Health	CADTH therapeutic review recommendations: new oral anticoagulants for the prevention of thromboembolic events in patients with atrial fibrillation	2012-June
284	C	Canadian Expert Panel on the Management of Age-related Macular Degeneration	Canadian expert consensus: optimal treatment of neovascular age-related macular degeneration	2012-June
285	C	Society of Obstetricians and Gynaecologists of Canada	Canadian HIV pregnancy planning guidelines	2012-June
286	M	Alberta Health Services - Cancer Care	Meningiomas	2012-June
287	P	Canadian Paediatric Society	Preventing playground injuries	2012-June
288	T	Canadian Paediatric Society	The Baby-friendly Initiative: Protecting, promoting and supporting breastfeeding	2012-June
289	T	Cancer Care Ontario's Program in Evidence-based Care	The role of liver resection in colorectal cancer metastases	2012-June
290	T	Canadian Cardiovascular Society	Transcatheter aortic valve implantation: A Canadian Cardiovascular Society position statement	2012-June
291	L	Société canadienne de pédiatrie	Les produits antimicrobiens à domicile : le problème de l'antibiorésistance	2006-mars (Reviewed 2012-juill)
292	S	Alberta Health Services - Cancer Care	Staging investigations for asymptomatic and newly diagnosed breast cancer	2011-Apr (Reviewed 2012-July)
293	A	Canadian Association for the Study of the Liver	An update on the management of chronic hepatitis C: Consensus guidelines from the Canadian Association for the Study of the Liver	2012-July
294	C	Parkinson Society Canada	Canadian guidelines on Parkinson's disease	2012-July
295	C	Cancer Care Ontario's Program in Evidence-based Care	Chemotherapy (i.e., Gemcitabine, Docetaxel Plus Gemcitabine, Doxorubicin, or Trabectedin) for inoperable, locally advanced, recurrent, or metastatic uterine leiomyosarcoma	2012-July
296	H	Toward Optimized Practice [Alberta]	Guideline for primary care management of headache in adults	2012-July

297	I	College of Dental Surgeons of British Columbia	Infection prevention and control guidelines	2012-July
298	L	Société Parkinson Canada	Lignes directrices canadiennes sur la maladie de Parkinson	2012-juill
299	C	Canadian Rheumatology Association	Canadian Rheumatology Association recommendations for the pharmacological management of rheumatoid arthritis with traditional and biologic disease-modifying antirheumatic drugs: Part II Safety	2012-Aug
300	F	Society of Obstetricians and Gynaecologists of Canada	Female sexual health consensus clinical guidelines	2012-Aug
301	P	Alberta Health Services - Cancer Care	Pituitary adenomas	2012-Aug
302	E	Society of Obstetricians and Gynaecologists of Canada	Emergency contraception	2003-Aug (Reviewed 2012-Sept)
303	R	Guidelines and Protocols Advisory Committee (BC)	Rheumatoid arthritis: Diagnosis, management and monitoring	2012-Sept-30
304	0-9	Canadian Association of Radiologists	2012 CAR diagnostic imaging referral guidelines - Section A: Central nervous system	2012-Sept
305	0-9	Canadian Association of Radiologists	2012 CAR diagnostic imaging referral guidelines - Section B: Head and neck	2012-Sept
306	0-9	Canadian Association of Radiologists	2012 CAR diagnostic imaging referral guidelines - Section C: Spine	2012-Sept
307	0-9	Canadian Association of Radiologists	2012 CAR diagnostic imaging referral guidelines - Section D: Musculoskeletal system	2012-Sept
308	0-9	Canadian Association of Radiologists	2012 CAR diagnostic imaging referral guidelines - Section E: Cardiovascular	2012-Sept
309	0-9	Canadian Association of Radiologists	2012 CAR diagnostic imaging referral guidelines - Section F: Thoracic	2012-Sept
310	0-9	Canadian Association of Radiologists	2012 CAR diagnostic imaging referral guidelines - Section G: Gastrointestinal system	2012-Sept
311	0-9	Canadian Association of Radiologists	2012 CAR diagnostic imaging referral guidelines - Section I: Obstetrics and gynaecology	2012-Sept
312	0-9	Canadian Association of Radiologists	2012 CAR diagnostic imaging referral guidelines - Section L: Pediatrics	2012-Sept

313	C	Heart and Stroke Foundation of Canada; Canadian Stroke Network	Canadian best practice recommendations for stroke care : chapter 2. stroke prevention	2012-Sept
314	L	Collège des médecins du Québec	L'interruption volontaire de grossesse	2012-sept
315	L	Les diététistes du Canada; Santé Canada; Breastfeeding Committee for Canada; Société canadienne de pédiatrie	La nutrition du nourrisson né à terme et en santé – Recommandations de la naissance à six mois: Énoncé conjoint de Santé Canada, de la Société canadienne de pédiatrie, des Diététistes du Canada et du Comité canadien pour l'allaitement	2012-sept
316	L	Société canadienne de pédiatrie	La prévention des blessures non intentionnelles chez les enfants et adolescents autochtones au Canada	2012-sept
317	L	Association canadienne des radiologistes	Lignes directrices pour les examens d'imagerie diagnostique de la CAR 2012 - Section A : Système nerveux central	2012-sept
318	L	Association canadienne des radiologistes	Lignes directrices pour les examens d'imagerie diagnostique de la CAR 2012 - Section B : Tête et cou	2012-sept
319	L	Association canadienne des radiologistes	Lignes directrices pour les examens d'imagerie diagnostique de la CAR 2012 - Section C : Rachis	2012-sept
320	L	Association canadienne des radiologistes	Lignes directrices pour les examens d'imagerie diagnostique de la CAR 2012 - Section D : Système musculosquelettique	2012-sept
321	L	Association canadienne des radiologistes	Lignes directrices pour les examens d'imagerie diagnostique de la CAR 2012 - Section E : Système cardiovasculaire	2012-sept
322	L	Association canadienne des radiologistes	Lignes directrices pour les examens d'imagerie diagnostique de la CAR 2012 - Section F : Thorax	2012-sept
323	L	Association canadienne des radiologistes	Lignes directrices pour les examens d'imagerie diagnostique de la CAR 2012 - Section G : Système gastro-intestinal	2012-sept

324	L	Association canadienne des radiologistes	Lignes directrices pour les examens d’imagerie diagnostique de la CAR 2012 - Section I : Obstétrique et gynécologie	2012-sept
325	L	Association canadienne des radiologistes	Lignes directrices pour les examens d’imagerie diagnostique de la CAR 2012 - Section L : Pédiatrie	2012-sept
326	N	Dietitians of Canada; Health Canada; Breastfeeding Committee for Canada; Canadian Paediatric Society	Nutrition for healthy term infants: Recommendations for birth to six months: A joint statement of Health Canada, Canadian Paediatric Society, Dietitians of Canada, and Breastfeeding Committee for Canada	2012-Sept
327	P	Canadian Paediatric Society	Preventing unintentional injuries in Indigenous children and youth in Canada	2012-Sept
328	C	Société des obstétriciens et gynécologues du Canada	Contraception d’urgence	2003-août (Reviewed 2012-sept)
329	O	Guidelines and Protocols Advisory Committee (BC)	Osteoporosis: Diagnosis, treatment and fracture prevention	2011-May (Reviewed 2012-Oct)
330	R	Canadian Task Force on Preventive Health Care	Recommendations on screening for type 2 diabetes in adults	2012-Oct-16
331	0-9	Canadian Urological Association	2012 update: Guidelines for adult urinary incontinence collaborative consensus document for the Canadian Urological Association	2012-Oct
332	C	Société des obstétriciens et gynécologues du Canada	Classification des césariennes au Canada : Les critères modifiés de Robson	2012-oct
333	C	Society of Obstetricians and Gynaecologists of Canada	Classification of Caesarean sections in Canada: The modified Robson criteria	2012-Oct
334	C	Canadian Paediatric Society	Counselling and management for anticipated extremely preterm birth	2012-Oct
335	H	Toward Optimized Practice [Alberta]	Guideline for vitamin D testing and supplementation in adults	2012-Oct
336	L	Société canadienne de pédiatrie	Les conseils et la prise en charge en prévision d’une très grande prématurité	2012-oct
337	M	Cancer Care Ontario's Program in Evidence-based Care	Models of care for cancer survivorship	2012-Oct

338	R	College of Family Physicians of Canada; Canadian Association of Perinatal, Women's Health Nurses; Canadian Association of Midwives; Society of Rural Physicians of Canada; Society of Obstetricians and Gynaecologists of Canada	Rural maternity care	2012-Oct
339	S	Collège des médecins de famille du Canada; Canadian Association of Perinatal, Women's Health Nurses; Association canadienne des sages-femmes; Société de la médecine rurale du Canada; Société des obstétriciens et gynécologues du Canada	Soins de maternité en région rurale	2012-oct
340	B	Canadian Paediatric Society	Bodychecking in youth ice hockey	2012-Nov
341	C	Public Health Agency of Canada	Canadian immunization guide : part 4. Active immunizing agents - Bacille Calmette-Guérin (BCG) Vaccine	2012-Nov
342	D	Canadian Medical Association	Determining medical fitness to operate motor vehicles : CMA driver's guide	2012-Nov
343	E	Canadian Association of Gastroenterology	Evaluation and management of skeletal health in celiac disease: position statement - abstract only	2012-Nov
344	H	Ontario Association of Medical Laboratories	Guidelines for the use of laboratory tests for assessment of iron overload	2012-Nov
345	N	Provincial Council for Maternal and Child Health (Ontario)	Neonatal abstinence syndrome clinical practice guidelines for Ontario	2012-Nov
346	S	Cancer Care Ontario's Program in Evidence-based Care	Surgical management of patients with lymph node metastases from cutaneous melanoma of the trunk or extremities	2012-Dec-5
347	L	Société canadienne de pédiatrie	L'administration postnatale de corticoïdes pour traiter ou prévenir les affections pulmonaires chroniques chez les prématurés	2012-déc

348	A	Canadian Association of Psychosocial Oncology; Canadian Partnership Against Cancer	A pan-Canadian practice guideline: prevention, screening, assessment and treatment of sleep disturbances in adults with cancer	2012-Dec
349	M	Canadian Association for the Study of the Liver	Management of chronic hepatitis B: Consensus guidelines	2012-Dec
350	P	Canadian Paediatric Society	Postnatal corticosteroids to treat or prevent chronic lung disease in preterm infants	2012-Dec
351	P	Canadian Association of Psychosocial Oncology; Canadian Partnership Against Cancer	Psychosocial health care needs assessment of adult cancer patients: a consensus-based guideline	2012-Dec
352	R	Canadian Consensus Conference on the Diagnosis and Treatment of Dementia	Recommendations of the 4th Canadian Consensus Conference on the Diagnosis and Treatment of Dementia (CCCDTD4)	2012-Dec
353	S	Cancer Care Ontario's Program in Evidence-based Care	Stem cell transplantation in lymphoma	2012-Dec
354	A	Alberta Health Services - Cancer Care	Alberta bone marrow and blood cell transplant program – Standard practice manual	2011
355	A	Canadian Centre on Substance Abuse	Alcohol and health in Canada: A summary of evidence and guidelines for low-risk drinking	2011
356	B	Centre for Addiction and Mental Health	Buprenorphine/Naloxone for opioid dependence: Clinical practice guideline	2011
357	C	Canadian Attention Deficit Hyperactivity Disorder Resource Alliance	Canadian ADHD practice guidelines, Third Edition	2011
358	C	Canadian Society of Nephrology	Clinical practice guidelines and recommendations on peritoneal dialysis adequacy 2011	2011
359	D	SCI Action Canada	Directives en matière d'activité physique chez les adultes ayant une lésion médullaire	2011
360	H	Toward Optimized Practice [Alberta]	Guideline for the evidence-informed primary care management of low back pain, 2nd Edition, 2011	2011
361	L	Canadian Attention Deficit Hyperactivity Disorder Resource Alliance	Lingnes directrices canadiennes sur le TDAH - Troisième édition	2011
362	N	Canadian Partnership Against Cancer	National colorectal cancer screening network classification of benign polyps	2011

363	P	Rick Hansen Institute; SCI Action Canada	Physical activity guidelines for adults with spinal cord injury	2011
364	P	Saskatchewan Cancer Agency	Provincial non small cell lung cancer treatment guidelines	2011
365	P	Saskatchewan Cancer Agency	Provincial small cell lung cancer treatment guidelines	2011
366	T	Association of Medical Microbiology and Infectious Disease Canada	The use of antiviral drugs for influenza: Guidance for practitioners, 2011-2012	2011
367	P	Registered Nurses' Association of Ontario	Promoting continence using prompted voiding	2002 (Reviewed 2011)
368	R	Registered Nurses' Association of Ontario	Risk assessment and prevention of pressure ulcers	2002 (Reviewed 2011)
369	P	Registered Nurses' Association of Ontario	Prevention of constipation in the older adult population	2005 (Reviewed 2011)
370	P	Registered Nurses' Association of Ontario	Prevention of falls and fall injuries in the older adult	2005 (Reviewed 2011)
371	S	Heart and Stroke Foundation of Ontario; Registered Nurses' Association of Ontario	Stroke assessment across the continuum of care	2005 (Reviewed 2011)
372	H	Perinatal Services BC	Guideline: Prenatal screening on Down Syndrome, trisomy 18, and open neural tube defects	2010-Jan (Reviewed 2011)
373	R	Registered Nurses' Association of Ontario	Reducing foot complications for people with diabetes	2004 (Reviewed 2011)
374	D	Société canadienne de pédiatrie	Des directives sur le dépistage génétique des enfants en santé	2003-janv (Reviewed 2011-janv)
375	H	Canadian Paediatric Society	Guidelines for genetic testing of healthy children	2003-Jan (Reviewed 2011-Jan)
376	B	Alberta Health Services - Cancer Care	Bladder cancer	2005-Apr (Reviewed 2011-Jan)
377	C	Canadian Paediatric Society	Children and natural health products : what a clinician should know	2005-Apr (Reviewed 2011-Jan)
378	L	Société canadienne de pédiatrie	Les enfants et les produits de santé naturels : ce que le clinicien devrait savoir	2005-avril (Reviewed 2011-janv)
379	C	Société des obstétriciens et gynécologues du Canada	Considérations génétiques pour ce qui est de l’évaluation préconceptionnelle chez la femme	2011-janv
380	D	Société canadienne de physiologie de l'exercice	Directives canadiennes en matière d’activité physique	2011-janv
381	L	Société canadienne de pédiatrie	Le traitement d’urgence de l’anaphylaxie chez les nourrissons et les enfants	2011-janv
382	U	Société canadienne de pédiatrie	Un calendrier de vaccination harmonisé au Canada : un appel à l’action	2011-janv

383	A	Canadian Paediatric Society	A harmonized immunization schedule for Canada: A call to action	2011-Jan
384	C	Canadian Society for Exercise Physiology	Canadian physical activity guidelines	2011-Jan
385	C	Canadian Thoracic Society	Canadian Thoracic Society 2011 guideline update: Diagnosis and treatment of sleep disordered breathing	2011-Jan
386	C	Guidelines and Protocols Advisory Committee (BC)	Chronic obstructive pulmonary disease (COPD)	2011-Jan
387	E	Canadian Paediatric Society	Emergency treatment of anaphylaxis in infants and children	2011-Jan
388	H	Society of Obstetricians and Gynaecologists of Canada	Genetic considerations for a woman’s pre-conception evaluation	2011-Jan
389	I	Expert Panel of Iron Overload in Myelodysplastic Syndromes	Iron overload in myelodysplastic syndromes: a Canadian consensus guideline	2011-Jan
390	T	Cancer Care Ontario's Program in Evidence-based Care	The role of IMRT in head & neck cancer	2011-Jan
391	T	British Columbia Centre for Excellence in HIV/AIDS	Therapeutic guidelines: antiretroviral treatment of adult HIV infection	2011-Jan
392	L	American Academy of Pediatrics; Société canadienne de pédiatrie	La prévention et la prise en charge de la douleur chez le nouveau-né : Une mise à jour	2007-janv (Reviewed 2011-fév)
393	U	Société canadienne de pédiatrie	Une discipline efficace auprès des enfants	2004-janv (Reviewed 2011-fév)
394	E	Canadian Paediatric Society	Effective discipline for children	2004-Jan (Reviewed 2011-Feb)
395	P	American Academy of Pediatrics; Canadian Paediatric Society	Prevention and management of pain in the neonate: An update	2007-Jan (Reviewed 2011-Feb)
396	L	Société canadienne de pédiatrie	La prévention et la prise en charge de la douleur chez le nouveau-né: une mise à jour	2007 (Reviewed 2011-fév)
397	P	American Academy of Pediatrics; Canadian Paediatric Society	Prevention and management of pain and stress in the neonate: an update	2007 (Reviewed 2011-Feb)
398	H	Canadian Paediatric Society	Guidelines for detection, management and prevention of hyperbilirubinemia in term and late preterm newborn infants (35 or more weeks' gestation)	2007-June (Reviewed 2011-Feb)

399	L	Société canadienne de pédiatrie	Lignes directrices pour la détection, la prise en charge et la prévention de l'hyperbilirubinémie chez les nouveau-nés à terme et peu prématurés (35 semaines d'âge gestationnel ou plus)	2007-juin (Reviewed 2011-fév)
400	L	Société canadienne de pédiatrie	La prise en charge du nourrisson plus vulnérable à la septicémie	2007-déc (Reviewed 2011-fév)
401	M	Canadian Paediatric Society	Management of the infant at increased risk for sepsis	2007-Dec (Reviewed 2011-Feb)
402	R	Alberta Health Services - Cancer Care	Referral and follow-up surveillance of cutaneous melanoma	2009-June (Reviewed 2011-Feb)
403	L	Société canadienne de pédiatrie	Lisez, parlez, chantez : la promotion de l'alphabétisation au cabinet du médecin	2006-nov (Reviewed 2011-fév)
404	R	Canadian Paediatric Society	Read, speak, sing : promoting literacy in the physician's office	2006-Nov (Reviewed 2011-Feb)
405	D	Société canadienne de physiologie de l'exercice	Directives canadiennes en matière de comportement sédentaire	2011-fév
406	H	Association Canadienne d'Oncologie Psychosociale; Partenariat canadien contre le cancer	Guide pancanadien de pratique clinique : dépistage, évaluation et prise en charge de la fatigue liée au cancer chez l'adulte	2011-fév
407	I	Société des obstétriciens et gynécologues du Canada	Interventions de treillis transvaginales visant le prolapsus des organes pelviens	2011-fév
408	L	Société canadienne de pédiatrie	La prémédication en vue de l'intubation trachéale du nouveau-né	2011-fév
409	L	Société canadienne de pédiatrie	La prise en charge d'urgence du patient pédiatrique en état de mal épileptique convulsif généralisé	2011-fév
410	L	Société canadienne de pédiatrie	L'accessibilité de la thérapie intraveineuse à domicile pour les enfants et adolescents canadiens	2011-fév
411	L	Société canadienne de pédiatrie	Le congé sécuritaire du nourrisson peu prématuré	2011-fév
412	C	Canadian Critical Care Trials Group; Canadian Critical Care Society	Clinical practice guidelines for the use of noninvasive positive-pressure ventilation and noninvasive continuous positive airway pressure in the acute care setting	2011-Feb-14

413	A	Canadian Association of Psychosocial Oncology; Canadian Partnership Against Cancer	A pan-Canadian practice guideline: screening, assessment and care of cancer-related fatigue in adults with cancer	2011-Feb
414	C	Canadian Cardiovascular Society	Canadian Cardiovascular Society atrial fibrillation guidelines 2010: Catheter ablation for atrial fibrillation/atrial flutter	2011-Feb
415	C	Canadian Cardiovascular Society	Canadian Cardiovascular Society atrial fibrillation guidelines 2010: Etiology and initial investigations	2011-Feb
416	C	Canadian Cardiovascular Society	Canadian Cardiovascular Society atrial fibrillation guidelines 2010: Management of recent-onset atrial fibrillation and flutter in the emergency department	2011-Feb
417	C	Canadian Cardiovascular Society	Canadian Cardiovascular Society atrial fibrillation guidelines 2010: Prevention and treatment of atrial fibrillation following cardiac surgery	2011-Feb
418	C	Canadian Cardiovascular Society	Canadian Cardiovascular Society atrial fibrillation guidelines 2010: Prevention of stroke and systemic thromboembolism in atrial fibrillation and flutter	2011-Feb
419	C	Canadian Cardiovascular Society	Canadian Cardiovascular Society atrial fibrillation guidelines 2010: Rate and rhythm management	2011-Feb
420	C	Canadian Cardiovascular Society	Canadian Cardiovascular Society atrial fibrillation guidelines 2010: Surgical therapy	2011-Feb
421	C	Canadian Working Group on Acute and Chronic Rhinosinusitis	Canadian clinical practice guidelines for acute and chronic rhinosinusitis	2011-Feb
422	C	Canadian Society for Exercise Physiology	Canadian sedentary behaviour guidelines	2011-Feb
423	E	Canadian Paediatric Society	Emergency management of the paediatric patient with generalized convulsive status epilepticus	2011-Feb
424	M	College of Physicians and Surgeons of Ontario	Methadone maintenance treatment program standards and clinical guidelines, 4th edition February 2011	2011-Feb

425	S	Canadian Paediatric Society	Safe discharge of the late preterm infant	2011-Feb
426	S	Perinatal Services BC	Safe sleep environment guideline for infants 0 - 12 months of age	2011-Feb
427	T	Society of Obstetricians and Gynaecologists of Canada	Transvaginal mesh procedures for pelvic organ prolapse	2011-Feb
428	M	Alberta Health Services - Cancer Care	Merkel cell carcinoma	2008-May (Reviewed 2011-Feb)
429	L	Agence de santé publique du Canada	La déclaration d'effets indésirables d'antiviraux lors d'une pandémie de grippe – Ligne directrices pour les professionnels de la santé et les consommateurs	2009-avril-30 (Reviewed 2011-mars-2)
430	R	Public Health Agency of Canada	Reporting Adverse Reactions to Antiviral Drugs During an Influenza Pandemic – Guidelines for Health Professionals and Consumers	2009-Apr-30 (Reviewed 2011-Mar-2)
431	L	Société canadienne de pédiatrie	Les directives sur l'équipement minimal en vue des soins préhospitaliers en pédiatrie	2011-mars
432	P	Société des gynécologues oncologues du Canada; Société canadienne des colposcopistes; Société des obstétriciens et gynécologues du Canada	Prévention du cancer du col utérin au sein de milieux ne disposant que de faibles ressources	2011-mars
433	C	Cardiometabolic Risk Working Group	Cardiometabolic risk in Canada: A detailed analysis and position paper by the cardiometabolic risk working group - abstract only	2011-Mar
434	C	Society of Gynecologic Oncologists of Canada; Society of Canadian Colposcopists; Society of Obstetricians and Gynaecologists of Canada	Cervical cancer prevention in low-resource settings	2011-Mar
435	M	Canadian Thoracic Society	Managing dyspnea in patients with advanced chronic obstructive pulmonary disease: A Canadian Thoracic Society clinical practice guideline	2011-Mar
436	M	Canadian Paediatric Society	Minimum equipment guidelines for paediatric prehospital care	2011-Mar
437	N	Perinatal Services BC	Newborn nursing care pathway	2011-Mar
438	P	Perinatal Services BC	Postpartum nursing care pathway	2011-Mar

439	P	Canadian Paediatric Society	Premedication for endotracheal intubation in the newborn infant	2011-Mar
440	R	Canadian Cardiovascular Society; Canadian Heart Rhythm Society	Recommendations for the use of genetic testing in the clinical evaluation of inherited cardiac arrhythmias associated with sudden cardiac death: Canadian Cardiovascular Society/Canadian Heart Rhythm Society joint position paper	2011-Mar
441	S	Canadian Cardiovascular Society	Smoking cessation and the cardiovascular specialist: Canadian Cardiovascular Society Position Paper	2011-Mar
442	S	Canadian Cardiovascular Society	Standardized approaches to the investigation of syncope: Canadian Cardiovascular Society position paper	2011-Mar
443	S	Canadian Cardiovascular Society; Canadian Association of Cardiac Rehabilitation	Systematizing inpatient referral to cardiac rehabilitation 2010: Canadian Association of Cardiac Rehabilitation and Canadian Cardiovascular Society joint position paper	2011-Mar
444	C	Société des obstétriciens et gynécologues du Canada	Consommation de substances psychoactives pendant la grossesse	2011-avril
445	D	Comité consultatif de la médicine tropicale et de la médecine des voyages (CCMTMV)	Déclaration sur la protection contre l’encéphalite japonaise	2011-avril
446	L	Société canadienne de pédiatrie	L'Ankyloglossie et l'allaitement	2011-avril
447	O	Guidelines and Protocols Advisory Committee (BC)	Overweight and obese adults - Diagnosis and management	2011-Apr-1
448	R	Alberta Health Services - Cancer Care	Risk reduction and surveillance strategies for individuals at high genetic risk for breast and ovarian cancer	2007-Dec (Reviewed 2011-Apr)
449	0-9	Canadian Consortium on Evidence-Based Care in MDS	5-Azacytidine in myelodysplastic syndromes: A clinical practice guideline	2011-Apr
450	A	Canadian Paediatric Society	Ankyloglossia and breastfeeding	2011-Apr
451	C	Canadian Association of Radiologists	CAR Standard for Magnetic Resonance Imaging	2011-Apr
452	C	Canadian Association of Radiologists	CAR standard for performing scrotal ultrasound examinations	2011-Apr
453	C	Canadian Association of Radiologists	CAR Standard for performing thyroid and parathyroid ultrasound examinations	2011-Apr

454	O	Ottawa Panel	Ottawa Panel evidence-based clinical practice guidelines for the management of osteoarthritis in adults who are obese or overweight	2011-Apr
455	S	Committee to Advise on Tropical Medicine and Travel (CATMAT)	Statement on protection against Japanese encephalitis	2011-Apr
456	S	Society of Obstetricians and Gynaecologists of Canada	Substance use in pregnancy	2011-Apr
457	T	Trauma Association of Canada	Trauma Association of Canada pediatric subcommittee national pediatric cervical spine evaluation pathway: Consensus guidelines	2011-Apr
458	A	Alberta Health Services - Cancer Care	Aromatase inhibitors as adjuvant therapy in postmenopausal women with early-stage hormone receptor positive breast cancer	2006-Aug (Reviewed 2011-May)
459	0-9	Canadian Agency for Drugs and Technologies in Health	1.5 tesla magnetic resonance imaging scanners compared with 3.0 tesla magnetic resonance imaging scanners: systematic review of clinical effectiveness	2011-May
460	0-9	Canadian Hypertension Education Program	2011 CHEP recommendations for the management of hypertension	2011-May
461	H	Cancer Care Ontario's Program in Evidence-based Care	Guideline on hormone receptor testing in breast cancer	2011-May
462	M	Society of Obstetricians and Gynaecologists of Canada	Magnesium sulphate for fetal neuroprotection	2011-May
463	M	Perinatal Services BC	Management guideline for pregnant women and neonates born to women with suspected or confirmed pandemic H1N1 influenza (swine origin A/H1N1 influenza)	2011-May
464	P	British Columbia Centre for Excellence in HIV/AIDS	Primary care guidelines for the management of HIV/AIDs	2011-May
465	P	Colloquium on Guidelines for the Primary Health Care of Adults with Developmental Disabilities	Primary care of adults with developmental disabilities : Canadian consensus guidelines	2011-May
466	P	Saskatchewan Cancer Agency	Provincial esophageal cancer and gastro-esophageal junction cancer treatment guidelines	2011-May

467	T	Canadian Cardiovascular Society	The 2011 Canadian Cardiovascular Society heart failure management guidelines update: Focus on sleep apnea, renal dysfunction, mechanical circulatory support, and palliative care	2011-May
468	T	Canadian Cardiovascular Society	The use of antiplatelet therapy in the outpatient setting: Canadian Cardiovascular Society guidelines	2011-May
469	U	Society of Obstetricians and Gynaecologists of Canada	Ultrasonographic cervical length assessment in predicting preterm birth in singleton pregnancies	2011-May
470	U	Canadian Paediatric Society	Universal newborn hearing screening	2011-May
471	L	Société canadienne de pédiatrie	Le dépistage universel des troubles de l'audition chez les nouveau-nés	2011-mai
472	R	Société des obstétriciens et gynécologues du Canada	Recours à l'évaluation échographique de la longueur cervicale pour prédire l'accouchement préterme dans le cadre de grossesses monofoetales	2011-mai
473	R	Société des obstétriciens et gynécologues du Canada	Recours au sulfate de magnésium à des fins de neuroprotection fœtale	2011-mai
474	S	Colloque concernant les lignes directrices sur les soins de santé primaires aux adultes ayant une déficience développementale	Soins primaires aux adultes ayant une deficience developpementale: Lignes directrices consensuelles canadiennes	2011-mai
475	E	Alberta Health Services - Cancer Care	Esophageal cancer	2010-Mar (Reviewed 2011-June)
476	H	Alberta Health Services - Cancer Care	Gastric cancer	2010-Mar (Reviewed 2011-June)
477	C	Canadian Association of Radiologists	CAR standards for irreversible compression in digital diagnostic imaging within radiology	2010-Apr (Reviewed 2011-June)
478	A	Alberta Health Services - Cancer Care	Adenocarcinoma of the pancreas	2008-Jan (Reviewed 2011-June)
479	A	Alberta Health Services - Cancer Care	Anal canal cancer	2008-Jan (Reviewed 2011-June)
480	M	Alberta Health Services - Cancer Care	Metastatic colorectal cancer	2008-Jan (Reviewed 2011-June)
481	E	Alberta Health Services - Cancer Care	Early stage colon cancer	2008-Feb (Reviewed 2011-June)
482	S	Alberta Health Services - Cancer Care	Stages II and III colorectal cancer surveillance	2008-Feb (Reviewed 2011-June)

483	A	Cancer Care Ontario's Program in Evidence-based Care	Adjuvant systemic chemotherapy for stage II and III colon cancer following complete resection: An updated practice guideline	2011-June
484	C	Canadian Association of Radiologists	Consensus guidelines for the prevention of contrast induced nephropathy	2011-June
485	E	American Academy of Pediatrics; Canadian Paediatric Society	Early childhood caries in Indigenous communities	2011-June
486	H	Mental Health Commission of Canada	Guidelines for comprehensive mental health services for older adults in Canada	2011-June
487	N	Alberta Health Services - Cancer Care	Non-small cell lung cancer stage I	2011-June
488	N	Alberta Health Services - Cancer Care	Non-small cell lung cancer stage II	2011-June
489	N	Alberta Health Services - Cancer Care	Non-small cell lung cancer stage IV	2011-June
490	P	Saskatchewan Cancer Agency	Provincial colorectal cancer treatment guidelines	2011-June
491	T	Guidelines and Protocols Advisory Committee (BC)	Testosterone testing - Protocol	2011-June
492	T	Canadian Paediatric Society	The sexual abuse of young people with a disability or chronic health condition	2011-June
493	T	Cancer Care Ontario's Program in Evidence-based Care	The use of Folfirinox as first-line treatment for metastatic pancreatic Adenocarcinoma	2011-June
494	U	Society of Obstetricians and Gynaecologists of Canada	Ultrasound in twin pregnancies	2011-June
495	E	Société des obstétriciens et gynécologues du Canada	Échographie et grossesse gémellaire	2011-juin
496	L	American Academy of Pediatrics; Société canadienne de pédiatrie	La carie de la petite enfance dans les communautés autochtones	2011-juin
497	L	Société canadienne de pédiatrie	Les agressions sexuelles des jeunes ayant une incapacité ou une maladie chronique	2011-juin
498	M	Cancer Care Ontario's Program in Evidence-based Care	Management of suspicious adnexal mass	2011-July-7
499	E	Canadian Collaboration for Immigrant and Refugee Health	Evidence-based clinical guidelines for immigrants and refugees - Canadian guidelines for immigrant health	2011-July

500	E	Canadian Collaboration for Immigrant and Refugee Health	Evidence-based clinical guidelines for immigrants and refugees - Canadian guidelines for immigrant health : Iron-deficiency anemia (page E891 - E894)	2011-July
501	P	Canadian College of Medical Geneticists; Society of Obstetricians and Gynaecologists of Canada	Prenatal screening for and diagnosis of aneuploidy in twin pregnancies	2011-July
502	P	Canadian College of Medical Geneticists; Society of Obstetricians and Gynaecologists of Canada	Prenatal screening for fetal aneuploidy in singleton pregnancies	2011-July
503	T	Canadian Hypertension Education Program	The 2011 Canadian Hypertension Education Program recommendations for the management of hypertension: blood pressure measurement, diagnosis, assessment of risk, and therapy	2011-July
504	D	Société des obstétriciens et gynécologues du Canada	Dépistage et diagnostic prénatals de l'aneuploïdie en ce qui concerne les grossesses gémellaires	2011-juill
505	D	Collège canadien de généticiens médicaux; Société des obstétriciens et gynécologues du Canada	Dépistage prénatal de l'aneuploïdie foetale en ce qui concerne les grossesses monofoetales	2011-juill
506	S	Alberta Health Services - Cancer Care	Squamous cell carcinoma of the vulva	2008-Nov (Reviewed 2011-Aug)
507	A	Guidelines and Protocols Advisory Committee (BC)	Abnormal liver chemistry - Evaluation and interpretation	2011-Aug-1
508	B	American Academy of Pediatrics; Canadian Paediatric Society	Boxing participation by children and adolescents	2011-Aug
509	C	College of Physicians and Surgeons of Ontario	Clinical practice parameters and facility standards - Nuclear medicine - Fourth Edition, August 2011	2011-Aug
510	C	Cancer Care Ontario's Program in Evidence-based Care	Clinical utility of positron emission tomography in the diagnosis, staging, and management of sarcoidosis: guideline recommendations	2011-Aug
511	M	Society of Obstetricians and Gynaecologists of Canada	Maternity leave in normal pregnancy	2011-Aug
512	P	Canadian Urological Association	Prostate cancer screening: Canadian guidelines 2011	2011-Aug

513	R	Cancer Care Ontario's Program in Evidence-based Care	Radionuclide therapy for neuroendocrine malignancies	2011-Aug
514	S	Society of Obstetricians and Gynaecologists of Canada	Sexual and reproductive health counselling by health care professionals	2011-Aug
515	U	Alberta Health Services - Cancer Care	Uterine sarcoma	2011-Aug
516	C	Société des obstétriciens et gynécologues du Canada	Congé de maternité dans le cadre d'une grossesse normale	2011-août
517	C	Société des obstétriciens et gynécologues du Canada	Counseling sur la santé sexuelle et génésique offert par les professionnels de la santé	2011-août
518	L	American Academy of Pediatrics; Société canadienne de pédiatrie	La participation des enfants et des adolescents à la boxe	2011-août
519	P	Collège des médecins du Québec	Procédures et interventions en milieu extrahospitalier	2011-août
520	T	Cancer Care Ontario's Program in Evidence-based Care	The role of gemcitabine in the management of metastatic breast cancer : a clinical practice guideline	2007-Jan (Reviewed 2011-Sept-15)
521	A	Cancer Care Ontario's Program in Evidence-based Care	Adjuvant chemotherapy following complete resection of soft tissue sarcoma in adults	2002-Oct (Reviewed 2011-Sept)
522	D	Cancer Care Ontario's Program in Evidence-based Care	Doxorubicin-based chemotherapy for the palliative treatment of adult patients with locally advanced or metastatic soft tissue sarcoma	2001-July (Reviewed 2011-Sept)
523	E	Cancer Care Ontario's Program in Evidence-based Care	Epidermal growth factor receptor (EGFR) targeted therapy in stage III and IV head and neck cancer	2009-May-15 (Reviewed 2011-Sept-30)
524	I	Cancer Care Ontario's Program in Evidence-based Care	Interleukin-2 in the treatment of patients with unresectable or metastatic renal cell cancer	2006-June-8 (Reviewed 2011-Sept-15)
525	P	Guidelines and Protocols Advisory Committee (BC)	Palliative care for the patient with incurable cancer or advanced disease - Part 2: Pain and symptom management	2011-Sept-30
526	P	Guidelines and Protocols Advisory Committee (BC)	Palliative care for the patient with incurable cancer or advanced disease - Part 3: Grief and bereavement	2011-Sept-30
527	C	Canadian Rheumatology Association	Canadian Rheumatology Association recommendations for pharmacological management of rheumatoid arthritis with traditional and biologic disease-modifying antirheumatic drugs	2011-Sept

528	D	Canadian Working Group on Statin Adverse Effects	Diagnosis, prevention, and management of statin adverse effects and intolerance: Proceedings of a Canadian working group consensus conference	2011-Sept
529	E	Registered Nurses' Association of Ontario	End-of-life care during the last days and hours	2011-Sept
530	H	Eye Health Council of Ontario	Guidelines for the collaborative management of persons with diabetes mellitus by eye care professionals (page 26-35)	2011-Sept
531	L	Société canadienne de pédiatrie	La prévention de la varicelle : les recommandations à l'égard de la vaccination antivaricelleuse systématique à deux doses chez les enfants	2011-sept
532	L	Eye Health Council of Ontario	Lignes directrices sur la prise en charge concertée des personnes qui ont le diabète sucré par des professionnels des soins oculovisuels (page 38-48)	2011-sept
533	P	Canadian Paediatric Society	Preventing varicella: Recommendations for routine two-dose varicella immunization in children	2011-Sept
534	P	Saskatchewan Cancer Agency	Provincial central nervous system cancer treatment guidelines	2011-Sept
535	R	Collège canadien de généticiens médicaux; Société des obstétriciens et gynécologues du Canada	Recours à une méthode ADN (QF-PCR) dans le diagnostic prénatal des aneuploïdies foetales	2011-sept
536	R	Canadian Association of Optometrists	Review of the Canadian Association of Optometrists frequency of eye examinations guideline - Summary: An evidence-based approach (page 15-16)	2011-Sept
537	R	L'Association canadienne des optométristes	Révision des lignes directrices de l'Association canadienne des optométristes sur la fréquence des examens de la vue – sommaire: Approche factuelle (page 18-19)	2011-sept
538	U	Canadian College of Medical Geneticists; Society of Obstetricians and Gynaecologists of Canada	Use of a DNA Method, QF-PCR, in the prenatal diagnosis of fetal aneuploidies	2011-Sept
539	R	Alberta Health Services - Cancer Care	Renal cell carcinoma and genetic testing	2007-Dec (Reviewed 2011-Oct)

540	A	Société des obstétriciens et gynécologues du Canada	Autopsies foetales et périnatales dans les cas d’anomalies foetales diagnostiquées avant la naissance en présence d’un caryotype normal	2011-oct
541	C	Canadian College of Medical Geneticists	Canadian College of Medical Geneticists guidelines for the indications, analysis, and reporting of cancer specimens	2011-Oct
542	C	Cancer Care Ontario's Program in Evidence-based Care	Cervical screening	2011-Oct
543	F	Society of Obstetricians and Gynaecologists of Canada	Fetal and perinatal autopsy in prenatally diagnosed fetal abnormalities with normal karyotype	2011-Oct
544	H	Canadian Urological Association	Guidelines for the diagnosis and management of recurrent urinary tract infection in women	2011-Oct
545	L	Société canadienne de pédiatrie	La prévention des infections par le virus respiratoire syncytial	2011-oct
546	L	Société canadienne de pédiatrie	Les vaccins contre le méningocoque au Canada : une mise à jour	2011-oct
547	L	Commission de la santé mentale du canada	Lignes directrices relatives à la planification et la prestation de services complets en santé mentale pour les aînés canadiens	2011-oct
548	L	Société canadienne de pédiatrie	L’utilisation du vaccin contre l’influenza chez les enfants allergiques aux œufs	2011-oct
549	M	Canadian Paediatric Society	Meningococcal vaccines in Canada: An update	2011-Oct
550	P	Canadian Paediatric Society	Preventing respiratory syncytial virus infections	2011-Oct
551	U	Canadian Paediatric Society	Use of influenza vaccines in children with an egg allergy	2011-Oct
552	H	Toward Optimized Practice [Alberta]	Guideline and screening for cervical cancer	2007-Feb (Reviewed 2011-Nov)
553	O	Cancer Care Ontario's Program in Evidence-based Care	Optimal chemotherapy for recurrent ovarian cancer	2011-Nov-21
554	A	Toward Optimized Practice [Alberta]	Active tuberculosis : Diagnosis and management guideline	2011-Nov
555	A	Society of Obstetricians and Gynaecologists of Canada	Advanced reproductive age and fertility	2011-Nov
556	A	Société des obstétriciens et gynécologues du Canada	Âge génésique avancé et fertilité	2011-nov

557	B	Cancer Care Ontario's Program in Evidence-based Care	Baseline staging tests in primary breast cancer	2011-Nov
558	D	Société canadienne de fertilité et d'andrologie; Société des obstétriciens et gynécologues du Canada	Diagnostic et prise en charge du syndrome d’hyperstimulation ovarienne	2011-nov
559	L	Société canadienne de pédiatrie	Les inhibiteurs spécifiques du recaptage de la sérotonine pendant la grossesse et l’issue du nourrisson	2011-nov
560	O	Canadian Agency for Drugs and Technologies in Health	Optimal use recommendations for atypical antipsychotics: combination and high-dose treatment strategies in adolescents and adults with schizophrenia	2011-Nov
561	O	Osteoporosis Canada	Osteoporosis Canada 2010 Guidelines for the assessment of fracture risk - abstract only	2011-Nov
562	R	Canadian Agency for Drugs and Technologies in Health	Recommendations for optimal warfarin management for prevention of thromboembolic events in patients with atrial fibrillation	2011-Nov
563	R	Canadian Task Force on Preventive Health Care	Recommendations on screening for breast cancer in average-risk women aged 40–74 years	2011-Nov
564	S	Canadian Paediatric Society	Selective serotonin reuptake inhibitors in pregnancy and infant outcomes	2011-Nov
565	T	Canadian Fertility and Andrology Society; Society of Obstetricians and Gynaecologists of Canada	The diagnosis and management of ovarian hyperstimulation syndrome	2011-Nov
566	S	Alberta Health Services - Cancer Care	Sentinel lymph node biopsy and axillary node dissection in early stage breast cancer	2007-Jan (Reviewed 2011-Dec)
567	B	Société canadienne de pédiatrie	Bien faire ce qu’il faut à 18 mois : en appui au bilan de santé amélioré	2011-déc
568	L	Société canadienne de pédiatrie	Les mesures, en appui au développement de la petite enfance	2011-déc
569	R	Société des obstétriciens et gynécologues du Canada	Recours à la technologie d’hybridation génomique matricielle dans le cadre du diagnostic prénatal au Canada	2011-déc
570	H	Canadian Paediatric Society	Getting it right at 18 months: In support of an enhanced well-baby visit	2011-Dec

571	M	Canadian Paediatric Society	Measuring in support of early childhood development	2011-Dec
572	U	Society of Obstetricians and Gynaecologists of Canada	Use of array genomic hybridization technology in prenatal diagnosis in Canada	2011-Dec
573	C	Canadian Consensus Group on Treatment of HIV-infected Adults	Canadian consensus guidelines for the optimal use of maraviroc in the treatment of HIV-infected adults	2010
574	C	Guidelines and Protocols Advisory Committee (BC)	Cardiovascular disease - primary prevention	2010
575	I	Public Health Agency of Canada	Infection prevention and control guideline for flexible gastrointestinal endoscopy and flexible bronchoscopy	2010
576	C	Canadian Cardiovascular Society; Canadian Academy of Child and Adolescent Psychiatry; Canadian Paediatric Society	Cardiac risk assessment before the use of stimulant medications in children and youth	2009-Nov (Reviewed 2010)
577	L	Agence de santé publique du Canada	Lignes directrices canadiennes sur les infections transmissibles sexuellement - Mise-à-jour janvier 2010	2006 (Reviewed 2010-janv)
578	S	Registered Nurses' Association of Ontario	Screening for delirium, dementia and depression in older adults	2003 (Reviewed 2010)
579	N	Registered Nurses' Association of Ontario	Nursing care of dyspnea: The 6th vital sign in individuals with chronic obstructive pulmonary disease (COPD)	2005 (Reviewed 2010)
580	C	Registered Nurses' Association of Ontario	Caregiving strategies for older adults with delirium, dementia and depression	2004 (Reviewed 2010)
581	L	Agence de santé publique du Canada	Lignes directrices canadiennes sur les infections transmissibles sexuellement : Syphilis - Révisé janvier 2010	2006 (Reviewed 2010-janv)
582	C	Public Health Agency of Canada	Canadian guidelines on sexually transmitted infections - updated January 2010	2006 (Reviewed 2010-Jan)
583	C	Public Health Agency of Canada	Canadian guidelines on sexually transmitted infections : Syphilis - revised January 2010	2006 (Reviewed 2010-Jan)
584	C	Canadian Paediatric Society	Current management of herpes simplex virus infection in pregnant women and their newborn infants	2006-Aug (Reviewed 2010-Jan)
585	L	Société canadienne de pédiatrie	Le traitement familial des enfants et des adolescents anorexiques : Des lignes directrices pour le médecin communautaire	2010-janv

586	A	Guidelines and Protocols Advisory Committee (BC)	Anxiety and depression in children and youth - diagnosis and treatment	2010-Jan-1
587	T	Guidelines and Protocols Advisory Committee (BC)	Thyroid function tests in the diagnosis and monitoring of adults	2010-Jan-1
588	C	Canadian Association of Radiologists	CAR CT colonography standards	2010-Jan
589	C	Canadian Association of Radiologists	CAR technical standards for bone mineral densitometry reporting	2010-Jan
590	F	Canadian Paediatric Society	Family-based treatment of children and adolescents with anorexia nervosa: Guidelines for the community physician	2010-Jan
591	M	Society of Interventional Radiology; Canadian Interventional Radiology Association; American College of Phlebology; Cardiovascular and Interventional Radiological Society of Europe	Multi-disciplinary quality improvement guidelines for the treatment of lower extremity superficial venous insufficiency with ambulatory phlebectomy from the Society of Interventional Radiology, Cardiovascular Interventional Radiological Society of Europe, American College of Phlebology and Canadian Interventential Radiology Association	2010-Jan
592	O	Guidelines and Protocols Advisory Committee (BC)	Otitis media: Acute otitis media (AOM) & otitis media with effusion (OME)	2010-Jan
593	S	Society of Obstetricians and Gynaecologists of Canada	Supracervical hysterectomy	2010-Jan
594	T	Cancer Care Ontario's Program in Evidence-based Care	Treatment of anemia with erythropoietic agents in patients with cancer - adaption/Adoption of ASH-ASCO guidelines: Guideline recommendations	2010-Jan
595	H	Guidelines and Protocols Advisory Committee (BC)	Gastroesophageal reflux disease - clinical approach in adults	2009-Jan-30 (Reviewed 2010-Jan-30)
596	H	Toward Optimized Practice [Alberta]	Guideline for adult insomnia : assessment to diagnosis	2006-Feb (Reviewed 2010-Feb)
597	H	Toward Optimized Practice [Alberta]	Guideline for adult primary insomnia: diagnosis to management	2006-Feb (Reviewed 2010-Feb)
598	S	Toward Optimized Practice [Alberta	Summary for the diagnosis and management of osteoporosis - Summary of the 2002 Canadian guidelines for the diagnosis and management of osteoporosis, revised 2010	2003 (Reviewed 2010-Feb)

599	R	Toward Optimized Practice [Alberta]	Recommendations for the diagnosis and management of hemochromatosis	2007-Feb (Reviewed 2010-Feb)
600	A	Alberta Health Services - Cancer Care	Anaplastic astrocytomas and oligodendrogliomas	2008-Apr (Reviewed 2010-Feb)
601	H	Alberta Health Services - Cancer Care	Glioblasoma	2008-Apr (Reviewed 2010-Feb)
602	H	Toward Optimized Practice [Alberta]	Guideline for the investigation and management of erectile dysfunction	2001 (Reviewed 2010-Feb)
603	M	Association des chemins de fer du Canada	Manuel du règlement médical des chemins de fer (postes classifiés comme essentiels pour la sécurité ferroviaire)	2010-fév
604	M	Association des chemins de fer du Canada	Manuel du règlement médical des chemins de fer (postes classifiés comme essentiels pour la sécurité ferroviaire) : Section 4 : lignes directrices médicales des compagnies ferroviaires : 4.10 Opioïdes à des fins thérapeutiques	2010-fév
605	M	Association des chemins de fer du Canada	Manuel du règlement médical des chemins de fer (postes classifiés comme essentiels pour la sécurité ferroviaire) : Section 4 : lignes directrices médicales des compagnies ferroviaires : 4.2 Audition	2010-fév
606	M	Association des chemins de fer du Canada	Manuel du règlement médical des chemins de fer (postes classifiés comme essentiels pour la sécurité ferroviaire) : Section 4 : lignes directrices médicales des compagnies ferroviaires : 4.3 Vision	2010-fév
607	M	Association des chemins de fer du Canada	Manuel du règlement médical des chemins de fer (postes classifiés comme essentiels pour la sécurité ferroviaire) : Section 4 : lignes directrices médicales des compagnies ferroviaires : 4.4 Troubles convulsifs	2010-fév
608	M	Association des chemins de fer du Canada	Manuel du règlement médical des chemins de fer (postes classifiés comme essentiels pour la sécurité ferroviaire) : Section 4 : lignes directrices médicales des compagnies ferroviaires : 4.5 Troubles mentaux	2010-fév

609	M	Association des chemins de fer du Canada	Manuel du règlement médical des chemins de fer (postes classifiés comme essentiels pour la sécurité ferroviaire) : Section 4 : lignes directrices médicales des compagnies ferroviaires : 4.6 Troubles cardiovasculaires	2010-fév
610	M	Association des chemins de fer du Canada	Manuel du règlement médical des chemins de fer (postes classifiés comme essentiels pour la sécurité ferroviaire) : Section 4 : lignes directrices médicales des compagnies ferroviaires : 4.7 Diabète	2010-fév
611	M	Association des chemins de fer du Canada	Manuel du règlement médical des chemins de fer (postes classifiés comme essentiels pour la sécurité ferroviaire) : Section 4 : lignes directrices médicales des compagnies ferroviaires : 4.8 Troubles liés à l'utilisation d'une substance	2010-fév
612	M	Association des chemins de fer du Canada	Manuel du règlement médical des chemins de fer (postes classifiés comme essentiels pour la sécurité ferroviaire) : Section 4 : lignes directrices médicales des compagnies ferroviaires 4.9 Apnée grave du sommeil	2010-fév
613	O	Société des obstétriciens et gynécologues du Canada	Obésité et grossesse	2010-fév
614	C	Railway Association of Canada	Canadian railway medical rules handbook (for positions critical to safe railway operations)	2010-Feb
615	C	Railway Association of Canada	Canadian railway medical rules handbook (for positions critical to safe railway operations) : Section 4 - Railway medical guidelines : 4.10 Therapeutic opioids	2010-Feb
616	C	Railway Association of Canada	Canadian railway medical rules handbook (for positions critical to safe railway operations) : Section 4 - Railway medical guidelines : 4.2 Hearing	2010-Feb
617	C	Railway Association of Canada	Canadian railway medical rules handbook (for positions critical to safe railway operations) : Section 4 - Railway medical guidelines : 4.3 Vision	2010-Feb

618	C	Railway Association of Canada	Canadian railway medical rules handbook (for positions critical to safe railway operations) : Section 4 - Railway medical guidelines : 4.4 Epileptic seizures	2010-Feb
619	C	Railway Association of Canada	Canadian railway medical rules handbook (for positions critical to safe railway operations) : Section 4 - Railway medical guidelines : 4.5 Mental disorders	2010-Feb
620	C	Railway Association of Canada	Canadian railway medical rules handbook (for positions critical to safe railway operations) : Section 4 - Railway medical guidelines : 4.6 Cardiovascular disorders	2010-Feb
621	C	Railway Association of Canada	Canadian railway medical rules handbook (for positions critical to safe railway operations) : Section 4 - Railway medical guidelines : 4.7 Diabetes	2010-Feb
622	C	Railway Association of Canada	Canadian railway medical rules handbook (for positions critical to safe railway operations) : Section 4 - Railway medical guidelines : 4.8 Substance use disorders	2010-Feb
623	C	Railway Association of Canada	Canadian railway medical rules handbook (for positions critical to safe railway operations) : Section 4 - Railway medical guidelines : 4.9 Severe sleep apnea	2010-Feb
624	C	Canadian Thoracic Society	Canadian Thoracic Society Asthma Management Continuum – 2010 consensus summary for children six years of age and over, and adults	2010-Feb
625	F	Cancer Care Ontario's Program in Evidence-based Care	First-line systemic chemotherapy in the treatment of advanced non-small cell lung cancer	2010-Feb
626	H	Canadian Coalition for Seniors' Mental Health	Guideline on the assessment and treatment of delirium in older adults at the end of life	2010-Feb
627	M	Perinatal Services BC	Maternity care pathway	2010-Feb
628	O	Society of Obstetricians and Gynaecologists of Canada	Obesity in pregnancy	2010-Feb
629	O	WorkSafe BC	Occupational dental erosion	2010-Feb
630	R	Toward Optimized Practice [Alberta]	Recommendations for MRSA a community problem	2010-Feb

631	S	Toward Optimized Practice [Alberta]	Summary for the diagnosis and management of COPD	2010-Feb
632	S	Toward Optimized Practice [Alberta]	Summary for the diagnosis of gluten-sensitive enteropathy (celiac disease) : A summary of the NASPGHAN guidelines	2010-Feb
633	C	Société des obstétriciens et gynécologues du Canada	Contraceptifs oraux et risque de thromboembolie veineuse : Mise à jour	2010-déc
634	L	Société canadienne de pédiatrie	Les visites en soins de santé préventifs pour les enfants et les adolescents de six à 17 ans : Le relevé médical Greig - Résumé	2010-mars
635	C	Canadian Cardiovascular Society	Canadian Cardiovascular Society 2009 consensus conference on the management of adults with congenital heart disease: Complex congenital cardiac lesions	2010-Mar
636	C	Canadian Cardiovascular Society	Canadian Cardiovascular Society 2009 Consensus Conference on the management of adults with congenital heart disease: Executive summary	2010-Mar
637	C	Canadian Cardiovascular Society	Canadian Cardiovascular Society 2009 Consensus Conference on the management of adults with congenital heart disease: Introduction	2010-Mar
638	C	Canadian Cardiovascular Society	Canadian Cardiovascular Society 2009 Consensus Conference on the management of adults with congenital heart disease: Outflow tract obstruction, coarctation of the aorta, tetralogy of Fallot, Ebstein anomaly and Marfan's syndrome	2010-Mar
639	C	Canadian Cardiovascular Society	Canadian Cardiovascular Society 2009 Consensus Conference on the management of adults with congenital heart disease: Shunt lesions	2010-Mar
640	C	Association of Medical Microbiology and Infectious Disease Canada	Canadian practice guidelines for surgical intra-abdominal infections	2010-Mar
641	H	Toward Optimized Practice [Alberta]	Guideline for the diagnosis and management of urinary tract infections in long term care	2010-Mar

642	P	Canadian Paediatric Society	Preventive health care visits for children and adolescents aged six to 17 years : The Greig Health Record - Executive summary	2010-Mar
643	I	Société des obstétriciens et gynécologues du Canada	Infection à cytomégalovirus pendant la grossesse	2010-avril
644	L	Société canadienne de pédiatrie	L'endocardite infectieuse : Mise à jour des lignes directrice	2010-avril
645	L	Société canadienne de fertilité et d'andrologie; Société des obstétriciens et gynécologues du Canada	Le Transfert sélectif d'un seul embryon à la suite de la fécondation in vitro	2010-avril
646	L	Académie canadienne de la médecine du sport et de l'exercice; Société canadienne de pédiatrie	Les recommandations en matière d'activité physique pour les enfants ayant une maladie chronique précise : l'arthrite juvénile idiopathique, l'hémophilie, l'asthme ou la fibrose kystique	2010-avril
647	C	College of Physicians and Surgeons of Ontario	Clinical practice parameters and facility standards: Computed tomography - 2nd edition 2009 (revised April 2010)	2009 (Reviewed 2010-Apr)
648	C	College of Physicians and Surgeons of Ontario	Clinical Practice Parameters and Facility Standards: Magnetic resonance imaging - 2nd edition 2009 (Revised April 2010)	2009 (Reviewed 2010-Apr)
649	C	Association of Medical Microbiology and Infectious Disease Canada	Canadian clinical practice guidelines for invasive candidiasis in adults	2010-Apr
650	C	Canadian Germ Cell Cancer Consensus Conference	Canadian consensus guidelines for the management of testicular germ cell cancer	2010-Apr
651	C	National Opioid Use Guideline Group	Canadian guideline for safe and effective use of opioids for chronic non-cancer pain - Part A: Executive summary and background	2010-Apr
652	C	National Opioid Use Guideline Group	Canadian guideline for safe and effective use of opioids for chronic non-cancer pain — Part B: Recommendations for practice	2010-Apr
653	C	Society of Obstetricians and Gynaecologists of Canada	Cytomegalovirus infection in pregnancy	2010-Apr

654	E	Canadian Fertility and Andrology Society; Society of Obstetricians and Gynaecologists of Canada	Elective single embryo transfer following in vitro fertilization	2010-Apr
655	I	Canadian Paediatric Society	Infective endocarditis: Updated guidelines	2010-Apr
656	P	Canadian Academy of Sport and Exercise Medicine; Canadian Paediatric Society	Physical activity recommendations for children with specific chronic health conditions: Juvenile idiopathic arthritis, hemophilia, asthma and cystic fibrosis	2010-Apr
657	T	Canadian Cardiovascular Society	The 2010 Canadian Cardiovascular Society guidelines for the diagnosis and management of heart failure update: Heart failure in ethnic minority populations, heart failure and pregnancy, disease management, and quality improvement/assurance programs	2010-Apr
658	I	Cancer Care Ontario's Program in Evidence-based Care	Imatinib mesylate in the adjuvant treatment of gastrointestinal stromal tumours (GIST)	2010-May-31
659	P	Cancer Care Ontario's Program in Evidence-based Care	Primary excision margins and sentinel lymph node biopsy in clinically node-negative cutaneous melanoma of the trunk or extremities: Guideline recommendations	2010-May-17
660	M	Alberta Health Services - Cancer Care	Management of in-transit disease of the limbs	2010-May
661	O	National Opioid Use Guideline Group	Opioids for chronic noncancer pain: a new Canadian practice guideline	2010-May
662	O	Society of Obstetricians and Gynaecologists of Canada	Ovulation induction in polycystic ovary syndrome	2010-May
663	D	Société des obstétriciens et gynécologues du Canada	Déclenchement de l’ovulation en présence du syndrome des ovaires polykystiques	2010-mai
664	T	Cancer Care Ontario's Program in Evidence-based Care	The role of trastuzumab in adjuvant and neoadjuvant therapy in women with HER2/neu-overexpressing breast cancer: a clinical practice guideline	2006-Jan (Reviewed 2010-June)
665	U	Canadian Paediatric Society	Use and misuse of tobacco among aboriginal peoples - update 2006	2006-Dec (Reviewed 2010-June)

666	L	Société canadienne de pédiatrie	L'usage et le mésusage du tabac chez les autochtones - mise à jour 2006	2006-déc (Reviewed 2010-juin)
667	T	Cancer Care Ontario's Program in Evidence-based Care	The role of trastuzumab (Herceptin) in the treatment of women with HER2/neu-Overexpressing metastatic breast cancer	2005-Nov (Reviewed 2010-June)
668	A	Guidelines and Protocols Advisory Committee (BC)	Asthma - diagnosis and management	2005-May (Reviewed 2010-June)
669	I	Guidelines and Protocols Advisory Committee (BC)	Iron deficiency - Investigation and management	2010-June-15
670	P	Guidelines and Protocols Advisory Committee (BC)	Palliative care for the patient with incurable cancer or advanced disease - Part 1: approach to care	2010-June-15
671	0-9	Canadian Prostate Health Council	2010 Update: Guidelines for the management of benign prostatic hyperplasia	2010-June
672	A	Society of Obstetricians and Gynaecologists of Canada	Adhesion prevention in gynaecological surgery	2010-June
673	A	Cancer Care Ontario's Program in Evidence-based Care	Adjuvant ovarian ablation in the treatment of premenopausal women with early stage invasive breast cancer	2010-June
674	C	Canadian Urological Association	Canadian guidelines for treatment of non-muscle invasive bladder cancer: a focus on intravesical therapy	2010-June
675	C	Canadian Urological Association	CUA guidelines: The work-up of azoospermic males	2010-June
676	C	Ontario Agency for Health Protection and Promotion	Cyclosporiasis: A clinical practice guideline	2010-June
677	E	Canadian Psychiatric Association	Electroconvulsive therapy	2010-June
678	E	Canadian Urological Association	Evaluation and medical management of the kidney stone patient	2010-June
679	H	Ontario Association of Medical Laboratories	Guideline for the appropriate ordering of serum tests for 25-hydroxy vitamin D and 1,25-dihydroxy vitamin D (CLP026)	2010-June
680	H	Canadian Urological Association	Guidelines for the management of castrate resistant prostate cancer (CRPC)	2010-June
681	M	Canadian Expert Panel on the Management of Anaphylaxia	Management of anaphylaxis in primary care: Canadian expert consensus recommendations	2010-June

682	P	Dietitians of Canada; College of Family Physicians of Canada; Community Health Nurses of Canada; Canadian Paediatric Society	Promoting optimal monitoring of child growth in Canada: Using the new World Health Organization growth charts – Executive Summary	2010-June
683	R	Public Health Agency of Canada	Recommendations on interferon gamma release assays for the diagnosis of latent tuberculosis infection - 2010 update	2010-June
684	T	Alberta Health and Wellness	Tuberculosis prevention and control guidelines for Alberta	2010-June
685	V	Canadian Urological Association	Vasectomy update 2010	2010-June
686	V	Canadian Paediatric Society	Vegetarian diets in children and adolescents	2010-June
687	L	Société canadienne de pédiatrie	Les régimes végétariens chez les enfants et les adolescents	2010-juin
688	P	Société des obstétriciens et gynécologues du Canada	Prévention des adhérences en chirurgie gynécologique	2010-juin
689	P	Les diététistes du Canada; Collège des médecins de famille du Canada; Infirmières et Infirmièrs en santé communautaire du canada; Société canadienne de pédiatrie	Promouvoir la surveillance optimale de la croissance des enfants au Canada : L'utilisation des nouvelles courbes de croissance de l'Organisation mondiale de la santé – Résumé	2010-juin
690	R	Agence de santé publique du Canada	Recommandations sur les tests de libération d'interféron-gamma pour la détection de l'infection tuberculeuse latente – Mise à jour de 2010	2010-juin
691	T	Cancer Care Ontario's Program in Evidence-based Care	The treatment of locally advanced pancreatic cancer	2004-Feb (Reviewed 2010-July)
692	A	Cancer Care Ontario's Program in Evidence-based Care	Adjuvant radiotherapy following radical prostatectomy for pathologic T3 or margin-positive prostate cancer : Updated guideline recommendations 2010	2010-July-22
693	C	Canadian Collaboration for Immigrant and Refugee Health	Canadian guidelines for immigrant health - Common mental health problems in immigrants and refugees: general approach in primary care	2010-July
694	C	College of Physicians and Surgeons of Ontario	Clinical practice parameters and facility standards : Chronic kidney disease and dialysis	2010-July

695	I	Canadian Paediatric Society	Inhalant abuse	2010-July
696	L	Société canadienne de pédiatrie	L'abuse de substances volatiles	2010-juill
697	A	Society of Obstetricians and Gynaecologists of Canada	Alcohol use and pregnancy consensus clinical guidelines	2010-Aug
698	S	Cancer Care Ontario's Program in Evidence-based Care	Single-agent interleukin-2 in the treatment of metastatic melanoma : a clinical practice guideline	2006-Mar-20 (Reviewed 2010-Aug-4)
699	C	Canadian Agency for Drugs and Technologies in Health	CADTH therapeutic review panel recommendations: Third-line therapy for patients with type 2 diabetes inadequately controlled with metformin and a sulfonylurea	2010-Aug
700	E	Canadian Paediatric Society	Emergency contraception	2010-Aug
701	E	Society of Obstetricians and Gynaecologists of Canada	Endometriosis: Diagnosis and management	2010-Aug
702	E	Society of Obstetricians and Gynaecologists of Canada	Endometriosis: Diagnosis and management : Chapter 1: Introduction	2010-Aug
703	E	Society of Obstetricians and Gynaecologists of Canada	Endometriosis: Diagnosis and management : Chapter 2: Pain management	2010-Aug
704	E	Society of Obstetricians and Gynaecologists of Canada	Endometriosis: Diagnosis and management : Chapter 4: Surgical management of endometriosis	2010-Aug
705	E	Society of Obstetricians and Gynaecologists of Canada	Endometriosis: Diagnosis and management : Chapter 5: Surgical management of infertility associated with endometriosis	2010-Aug
706	E	Society of Obstetricians and Gynaecologists of Canada	Endometriosis: Diagnosis and management : Chapter 6: Medical treatment of infertility related to endometriosis	2010-Aug
707	E	Society of Obstetricians and Gynaecologists of Canada	Endometriosis: Diagnosis and management : Chapter 7: Endometriosis in adolescents	2010-Aug
708	E	Society of Obstetricians and Gynaecologists of Canada	Endometriosis: Diagnosis and management : Chapter 8: Endometriosis and cancer	2010-Aug
709	E	Society of Obstetricians and Gynaecologists of Canada	Endometriosis: Diagnosis and management: Chapter 3: Medical Management of pain associated with endometriosis	2010-Aug
710	H	Ontario Association of Medical Laboratories	Guideline for lipid testing in adults	2010-Aug

711	M	Alberta Health Services - Cancer Care	Medulloblastoma	2010-Aug
712	O	Canadian Agency for Drugs and Technologies in Health	Optimal therapy recommendations for the prescribing and use of second-line therapy for patients with diabetes inadequately controlled on metformin	2010-Aug
713	O	Canadian Thoracic Society	Optimizing pulmonary rehabilitation in chronic obstructive pulmonary disease - practical issues: A Canadian Thoracic Society clinical practice guideline	2010-Aug
714	D	Société des obstétriciens et gynécologues du Canada	Directive clinique de consensus sur la consommation d'alcool et la grossesse	2010-août
715	E	Société des obstétriciens et gynécologues du Canada	Endométriose : Diagnostic et prise en charge	2010-août
716	E	Société des obstétriciens et gynécologues du Canada	Endométriose : Diagnostic et prise en charge : Chapitre 2 : Soulagement de la douleur	2010-août
717	E	Société des obstétriciens et gynécologues du Canada	Endométriose : Diagnostic et prise en charge : Chapitre 3 : Prise en charge médicale de la douleur associée à l'endométriose	2010-août
718	E	Société des obstétriciens et gynécologues du Canada	Endométriose : Diagnostic et prise en charge : Chapitre 4 : Prise en charge chirurgicale de l'endométriose	2010-août
719	E	Société des obstétriciens et gynécologues du Canada	Endométriose : Diagnostic et prise en charge : Chapitre 5 : Prise en charge chirurgicale de l'infertilité associée à l'endométriose	2010-août
720	E	Société des obstétriciens et gynécologues du Canada	Endométriose : Diagnostic et prise en charge : Chapitre 6: Prise en charge médicale de l'infertilité associée à l'endométriose	2010-août
721	E	Société des obstétriciens et gynécologues du Canada	Endométriose : Diagnostic et prise en charge : Chapitre 7: Endométriose chez les adolescentes	2010-août
722	E	Société des obstétriciens et gynécologues du Canada	Endométriose : Diagnostic et prise en charge : Chapitre 8: Endométriose et cancer	2010-août
723	E	Société des obstétriciens et gynécologues du Canada	Endométriose: Diagnostic et prise en charge : Chapitre 1 : introduction	2010-août

724	L	Société canadienne de pédiatrie	La contraception d'urgence	2010-août
725	C	Cancer Care Ontario's Program in Evidence-based Care	Combined modality radiotherapy and chemotherapy in the non-surgical management of localized carcinoma of the esophagus	2005 (Reviewed 2010-Sept)
726	F	Guidelines and Protocols Advisory Committee (BC)	Febrile seizures	2010-Sept-1
727	O	Guidelines and Protocols Advisory Committee (BC)	Oral rehydration therapy (ORT) in children	2010-Sept-1
728	A	Société des obstétriciens et gynécologues du Canada	Antibioprophylaxie dans le cadre d'interventions obstétricales	2010-sept
729	A	Society of Obstetricians and Gynaecologists of Canada	Antibiotic prophylaxis in obstetric procedures	2010-Sept
730	C	Canadian Sleep Society; Canadian Thoracic Society	Canadian Sleep Society/Canadian Thoracic Society position paper on the use of portable monitoring for the diagnosis of obstructive sleep apnea/hypopnea in adults	2010-Sept
731	D	Guidelines and Protocols Advisory Committee (BC)	Diabetes care	2010-Sept
732	D	Société des obstétriciens et gynécologues du Canada	Directive clinique sur l'évaluation et la prise en charge de l'incontinence urinaire récurrente à la suite d'une chirurgie visant le plancher pelvien	2010-sept
733	E	Association des professeurs d'obstétrique et gynécologie du Canada; Société des obstétriciens et gynécologues du Canada	Examens pelviens menés par des étudiants en médecine	2010-sept
734	H	Ontario Association of Medical Laboratories	Guidelines for ordering diagnostic testing for viral hepatitis	2010-Sept
735	H	Society of Obstetricians and Gynaecologists of Canada	Guidelines for the evaluation and treatment of recurrent urinary incontinence following pelvic floor surgery	2010-Sept
736	P	Association of Professors of Obstetrics and Gynaecology of Canada; Society of Obstetricians and Gynaecologists of Canada	Pelvic examinations by medical students	2010-Sept

737	R	Comité consultatif national de l'immunisation	Recommandations relatives à l’administration de deux doses du vaccin contre la varicelle	2010-sept
738	U	Canadian Association of Radiologists	Ultrasound - Performing diagnostic obstetric ultrasound examinations	2010-Sept
739	L	Société canadienne de pédiatrie	La prise en charge de la gale	2001-déc (Reviewed 2010-oct)
740	S	Canadian Paediatric Society	Scabies management	2001-Dec (Reviewed 2010-Oct)
741	I	Cancer Care Ontario's Program in Evidence-based Care	Invasive Mediastinal Staging of Non-small Cell Lung Cancer	2010-Oct-18
742	V	Guidelines and Protocols Advisory Committee (BC)	Vitamin D testing protocol	2010-Oct-1
743	W	Guidelines and Protocols Advisory Committee (BC)	Warfarin therapy - management during invasive procedures and surgery	2010-Oct-1
744	W	Guidelines and Protocols Advisory Committee (BC)	Warfarin therapy management	2010-Oct-1
745	A	Society of Obstetricians and Gynaecologists of Canada	Asymptomatic endometrial thickening	2010-Oct
746	D	Société canadienne de pédiatrie	Des recommandations quant à l’usage des vaccins antirotavirus chez les nourrissons	2010-oct
747	E	Société des obstétriciens et gynécologues du Canada	Épaississement endométrial asymptomatique	2010-oct
748	H	Perinatal Services BC	Gestational diabetes mellitus screening and diagnosis	2010-Oct
749	R	Canadian Paediatric Society	Recommendations for the use of rotavirus vaccines in infants	2010-Oct
750	T	Cancer Care Ontario's Program in Evidence-based Care	The role of IMRT in breast cancer	2010-Oct
751	T	Cancer Care Ontario's Program in Evidence-based Care	The role of IMRT in central nervous system cancer	2010-Oct
752	T	Cancer Care Ontario's Program in Evidence-based Care	The role of IMRT in gastrointestinal cancers	2010-Oct
753	T	Cancer Care Ontario's Program in Evidence-based Care	The role of IMRT in gynecologic cancers	2010-Oct
754	T	Cancer Care Ontario's Program in Evidence-based Care	The role of IMRT in prostate cancer	2010-Oct
755	T	Cancer Care Ontario's Program in Evidence-based Care	The role of IMRT in skin cancers	2010-Oct

756	T	Cancer Care Ontario's Program in Evidence-based Care	The role of IMRT in soft-tissue sarcomas	2010-Oct
757	T	Cancer Care Ontario's Program in Evidence-based Care	The role of IMRT in thyroid cancers	2010-Oct
758	B	Cancer Care Ontario's Program in Evidence-based Care	Breast irradiation in women with early stage invasive breast cancer following breast conserving surgery	2002-Jan (Reviewed 2010-Nov)
759	0-9	Osteoporosis Canada	2010 clinical practice guidelines for the diagnosis and management of osteoporosis in Canada: Summary	2010-Nov-23
760	P	Cancer Care Ontario's Program in Evidence-based Care	Positron emission tomography in radiation treatment planning for lung cancer	2010-Nov-17
761	I	Société des obstétriciens et gynécologues du Canada	Infection récurrente des voies urinaires	2010-nov
762	L	Société canadienne de pédiatrie	Les banques de lait humain	2010-nov
763	R	Society of Obstetricians and Gynaecologists of Canada	Recurrent urinary tract infection	2010-Nov
764	S	Cancer Care Ontario's Program in Evidence-based Care	Surgical management of early-stage invasive breast cancer	2003-Jan (Reviewed 2010-Nov)
765	T	Cancer Care Ontario's Program in Evidence-based Care	The role of IMRT in lung cancer	2010-Nov
766	E	Registered Nurses' Association of Ontario	Enhancing healthy adolescent development	2002 (Reviewed 2010-Dec)
767	B	Cancer Care Ontario's Program in Evidence-based Care	Bevacizumab for the treatment of patients with glioblastoma multiforme that has relapsed or progressed following prior therapy	2010-Dec
768	C	Canadian Association of Gastroenterology	Canadian Association of Gastroenterology position statement on screening individuals at average risk for developing colorectal cancer: 2010	2010-Dec
769	C	Canadian Expert Panel on Management of Hand Eczema	Canadian hand dermatitis management guidelines	2010-Dec
770	D	Canadian Thoracic Society	Diagnostic evaluation and management of chronic thromboembolic pulmonary hypertension: A clinical practice guideline	2010-Dec
771	N	Perinatal Services BC	Newborn screening	2010-Dec

772	O	Society of Obstetricians and Gynaecologists of Canada	Oral contraceptives and the risk of venous thromboembolism: An update	2010-Dec
773	R	Help ELiminate Pain in KIDS	Reducing the pain of childhood vaccination: an evidence-based clinical practice guideline	2010-Dec
774	S	Toward Optimized Practice [Alberta]	Screening and early diagnosis of prostate cancer	2010-Dec
775	S	Cancer Care Ontario's Program in Evidence-based Care	Systemic therapy for advanced gastric cancer	2010-Dec
776	C	no listed author on CMA Infobase	Canadian consensus guidelines for the optimal use of etravirine in the treatment of HIV-infected adults	2009
777	C	Canadian Psoriasis Guidelines Committee	Canadian guideline for the management of plaque psoriasis	2009
778	H	Toward Optimized Practice [Alberta]	Guideline for red blood and plasma transfusion: A summary	2009
779	L	Canadian Psoriasis Guidelines Committee	Lignes directrices canadiennes pour la prise en charge du psoriasis en plaques	2009
780	O	Association of Medical Microbiology and Infectious Disease Canada	Optimal use of raltegravir (Isentress®) in the treatment of HIV-infected adults - Canadian consensus guidelines	2009
781	H	Toward Optimized Practice [Alberta]	Guideline for the management of acute asthma in adults and children	2006-Oct (Reviewed 2009)
782	T	Public Health Agency of Canada	The Canadian pandemic influenza plan for the health sector	2006-Dec (Reviewed 2009)
783	H	Toward Optimized Practice [Alberta]	Guideline for management of modifiable risk factors in adults at high risk for cardiovascular events	2005-Feb (Reviewed 2009)
784	H	Toward Optimized Practice [Alberta]	Guideline for cognitive impairment : dementia - diagnosis to management	2002-Jan (Reviewed 2009)
785	A	L'Association des infirmières et infirmiers autorisès de l'Ontario	Administration de l’insuline par voie sous-cutaneé chez les adultes qui ont le diabète de type 2	2004 (Reviewed 2009)
786	B	Registered Nurses' Association of Ontario	Best practice guideline for the subcutaneous administration of insulin in adults with type 2 diabetes	2004 (Reviewed 2009)
787	H	Toward Optimized Practice [Alberta]	Guideline for cognitive impairment : is this dementia? Symptoms to diagnosis	2007-Jan (Reviewed 2009)

788	H	Toward Optimized Practice [Alberta]	Guideline for diagnosis and treatment of chronic undiagnosed dyspepsia in adults	2007-Jan (Reviewed 2009)
789	H	Toward Optimized Practice [Alberta]	Guideline for surgical & non-surgical management of cataract in the otherwise healthy adult eye	2007 (Reviewed 2009)
790	H	Toward Optimized Practice [Alberta]	Guideline for treatment of gastroesophageal reflux disease (GERD) in adults	2007 (Reviewed 2009)
791	H	Toward Optimized Practice [Alberta]	Guideline for treatment of Helicobacter pylori infection in adults	2007 (Reviewed 2009)
792	I	Société des obstétriciens et gynécologues du Canada	Immunisation pendant la grossesse	2008-déc (Reviewed 2009)
793	I	Society of Obstetricians and Gynaecologists of Canada	Immunization in pregnancy	2008-Dec (Reviewed 2009)
794	R	Agence de santé publique du Canada	Recommandations mises à jour sur les tests de libération d'interféron-gamma pour la détection de l'infection tuberculeuse latente	2009-janv
795	S	Cancer Care Ontario's Program in Evidence-based Care	Stem cell transplantation in adults	2009-Jan-11
796	A	Guidelines and Protocols Advisory Committee (BC)	Ankle injury - X-ray for acute injury of the ankle or mid-foot	2009-Jan
797	A	Registered Nurses' Association of Ontario	Assessment and care of adults at rist for suicidal ideation and behaviour	2009-Jan
798	C	Canadian Cardiovascular Society	Canadian Cardiovascular Society Working Group: Providing a perspective on the 2007 focused update of the American College of Cardiology and American Heart Association 2004 guidelines for the management of ST elevation myocardial infarction	2009-Jan
799	C	Canadian Association of Radiologists	CAR guidelines and standards for cardiac computed tomography	2009-Jan
800	I	Provincial Blood Coordinating Office (BC)	Intravenous immune globulin (IVIG) utilization management program guidelines	2009-Jan
801	M	Society of Obstetricians and Gynaecologists of Canada	Menopause and osteoporosis update 2009	2009-Jan
802	M	Society of Obstetricians and Gynaecologists of Canada	Menopause and osteoporosis update 2009: Chapter 1: Towards a healthier lifestyle	2009-Jan

803	M	Society of Obstetricians and Gynaecologists of Canada	Menopause and osteoporosis update 2009: Chapter 2: Vasomotor symptoms	2009-Jan
804	M	Society of Obstetricians and Gynaecologists of Canada	Menopause and osteoporosis update 2009: Chapter 3: Cardiovascular disease (CVD)	2009-Jan
805	M	Society of Obstetricians and Gynaecologists of Canada	Menopause and osteoporosis update 2009: Chapter 4: Hormone therapy and breast cancer	2009-Jan
806	M	Society of Obstetricians and Gynaecologists of Canada	Menopause and osteoporosis update 2009: Chapter 5: Urogenital health	2009-Jan
807	M	Society of Obstetricians and Gynaecologists of Canada	Menopause and osteoporosis update 2009: Chapter 6: Mood, memory, and cognition	2009-Jan
808	M	Society of Obstetricians and Gynaecologists of Canada	Menopause and osteoporosis update 2009: Chapter 7: Bone health	2009-Jan
809	P	Cancer Care Ontario's Program in Evidence-based Care	PET imaging in brain cancer: recommendations	2009-Jan
810	P	Cancer Care Ontario's Program in Evidence-based Care	PET imaging in cervical cancer	2009-Jan
811	P	Cancer Care Ontario's Program in Evidence-based Care	PET imaging in colorectal cancer: recommendations	2009-Jan
812	P	Cancer Care Ontario's Program in Evidence-based Care	PET imaging in esophageal cancer	2009-Jan
813	P	Cancer Care Ontario's Program in Evidence-based Care	PET imaging in melanoma	2009-Jan
814	P	Cancer Care Ontario's Program in Evidence-based Care	PET imaging in ovarian cancer	2009-Jan
815	P	Cancer Care Ontario's Program in Evidence-based Care	PET imaging in pancreatic cancer	2009-Jan
816	P	Cancer Care Ontario's Program in Evidence-based Care	PET imaging in small cell lung cancer	2009-Jan
817	T	Thrombosis Interest Group of Canada	Thrombolytic therapy in children	2005-July (Reviewed 2009-Jan)
818	A	Société canadienne de pédiatrie	Approche déontologique de l'examen génital de l'enfant	1999-janv (Reviewed 2009-fév)
819	E	Canadian Paediatric Society	Ethical approach to genital examination in children	1999-Jan (Reviewed 2009-Feb)
820	C	British Columbia Cancer Agency	Cancer management guidelines	1995 (Reviewed 2009-Feb)
821	P	Cancer Care Ontario's Program in Evidence-based Care	PET imaging in testicular cancer	2009-Jan

822	L	Société canadienne de pédiatrie	Le dépistage des troubles de la vue chez les nourrissons, les enfants et les adolescents	2009-fév
823	0-9	Canadian Cardiovascular Society	2009 Canadian Cardiovascular Society Consensus Conference guidelines on heart failure, update 2009: Diagnosis and management of right-sided heart failure, myocarditis, device therapy and recent important clinical trials	2009-Feb
824	C	Canadian Association of Radiologists	Canadian Association of Radiologists: Consensus guidelines and standards for cardiac CT	2009-Feb
825	D	Thrombosis Interest Group of Canada	Duration of anticoagulant therapy for venous thromboembolism	2009-Feb
826	O	Thrombosis Interest Group of Canada	Overview of perioperative management of warfarin therapy	2009-Feb
827	T	British Columbia Centre for Excellence in HIV/AIDS	Therapeutic guidelines for HIV infected and HIV exposed children	2009-Feb
828	T	British Columbia Centre for Excellence in HIV/AIDS	Therapeutic guidelines: accidental exposure guidelines	2009-Feb
829	V	Canadian Paediatric Society	Vision screening in infants, children and youth	2009-Feb
830	T	Société des obstétriciens et gynécologues du Canada	Tenue systématique d'un examen échographique obstétrical au cours du deuxième trimestre: Contenu d'un examen et d'un rapport exhaustifs	2009-mars
831	M	Cancer Care Ontario's Program in Evidence-based Care	Management of squamous cell cancer of the anal canal	2009-Mar-31
832	H	Cancer Care Ontario's Program in Evidence-based Care	Gastroscopy following a positive fecal occult blood test and negative colonoscopy	2009-Mar-30
833	T	Thrombosis Interest Group of Canada	Traveller's thrombosis	2009-Mar-16
834	T	Thrombosis Interest Group of Canada	Thrombolytic therapy in peripheral arterial disease	2005-July (Reviewed 2009-Mar)
835	C	Canadian Association of Gastroenterology	Canadian Association of Gastroenterology clinical practice guidelines: the use of tumour necrosis factor-alpha antagonist therapy in Crohn's disease	2009-Mar

836	C	College of Physicians and Surgeons of Ontario	Clinical practice parameters and facility standards - Laser treatment of benign vascular lesions - 3rd edition, March 2009	2009-Mar
837	C	Society of Obstetricians and Gynaecologists of Canada	Content of a complete routine second trimester obstetrical ultrasound examination and report	2009-Mar
838	I	Guidelines and Protocols Advisory Committee (BC)	Infectious diarrhea - guideline for ordering stool specimens	2009-Mar
839	T	Canadian Cardiovascular Society	The ‘what, when, where, who and how?’ of cardiac computed tomography in 2009: Guidelines for the clinician	2009-Mar
840	V	Thrombosis Interest Group of Canada	VTE prophylaxis in major orthopedic surgery	2009-Mar
841	T	Thrombosis Interest Group of Canada	The antiphospholipid syndrome	2004-June (Reviewed 2009-Mar)
842	C	Canadian Council of Motor Transport Administration	CCMTA medical standards for drivers	2004-July (Reviewed 2009-Mar)
843	C	Canadian Council of Motor Transport Administration	CCMTA medical standards for drivers : cardiovascular diseases	2004-July (Reviewed 2009-Mar)
844	C	Canadian Council of Motor Transport Administration	CCMTA medical standards for drivers : cerebrovascular diseases	2004-July (Reviewed 2009-Mar)
845	C	Canadian Council of Motor Transport Administration	CCMTA medical standards for drivers : diseases of the nervous system	2004-July (Reviewed 2009-Mar)
846	C	Canadian Council of Motor Transport Administration	CCMTA medical standards for drivers : hearing	2004-July (Reviewed 2009-Mar)
847	C	Canadian Council of Motor Transport Administration	CCMTA medical standards for drivers : metabolic diseases	2004-July (Reviewed 2009-Mar)
848	C	Canadian Council of Motor Transport Administration	CCMTA medical standards for drivers : musculoskeletal disabilities	2004-July (Reviewed 2009-Mar)
849	C	Canadian Council of Motor Transport Administration	CCMTA medical standards for drivers : peripheral vascular diseases	2004-July (Reviewed 2009-Mar)
850	I	Thrombosis Interest Group of Canada	Inferior vena cava filters	2004-Nov (Reviewed 2009-Mar)
851	C	Canadian Council of Motor Transport Administration	CCMTA medical standards for drivers : psychiatric disorders	2004-July (Reviewed 2009-Mar)
852	C	Canadian Council of Motor Transport Administration	CCMTA medical standards for drivers : renal disease	2004-July (Reviewed 2009-Mar)
853	C	Canadian Council of Motor Transport Administration	CCMTA medical standards for drivers : respiratory diseases	2004-July (Reviewed 2009-Mar)

854	C	Canadian Council of Motor Transport Administration	CCMTA medical standards for drivers : restraint system exemptions	2004-July (Reviewed 2009-Mar)
855	C	Canadian Council of Motor Transport Administration	CCMTA medical standards for drivers : the aging driver	2004-July (Reviewed 2009-Mar)
856	C	Canadian Council of Motor Transport Administration	CCMTA medical standards for drivers : the effects of alcoho	2004-July (Reviewed 2009-Mar)
857	C	Canadian Council of Motor Transport Administration	CCMTA medical standards for drivers : the effects of drugs	2004-July (Reviewed 2009-Mar)
858	C	Canadian Council of Motor Transport Administration	CCMTA medical standards for drivers : vision	2004-July (Reviewed 2009-Mar)
859	P	Guidelines and Protocols Advisory Committee (BC)	Protocol for macroscopic and microscopic urinalysis and investigation of urinary tract infection	1998-Mar (Reviewed 2009-Apr)
860	M	Guidelines and Protocols Advisory Committee (BC)	Microscopic hematuria (persistant)	2004-June-1 (Reviewed 2009-Apr)
861	T	Cancer Care Ontario's Program in Evidence-based Care	The use of inhibitors of angiogenesis in patients with inoperable locally advanced or metastatic renal cell cancer	2009-Apr-30
862	S	Guidelines and Protocols Advisory Committee (BC)	Stroke and transient ischemic attack – Management and prevention	2009-Apr-29
863	T	Cancer Care Ontario's Program in Evidence-based Care	The use of bevacizumab in metastatic breast cancer	2009-Apr-17
864	F	Cancer Care Ontario's Program in Evidence-based Care	Follow-up for women after treatment for cervical cancer	2009-Apr-13
865	C	Canadian Cardiovascular Society	Canadian Cardiovascular Society consensus conference update on cardiac transplantation 2008: executive summary	2009-Apr
866	H	Thrombosis Interest Group of Canada	Guideline for using point of care INR monitoring of Warfarin therapy	2009-Apr
867	M	Society of Obstetricians and Gynaecologists of Canada	Management guidelines for obstetric patients and neonates born to mothers with suspected or Probable Severe Acute Respiratory Syndrome (SARS)	2009-Apr

868	L	Société des obstétriciens et gynécologues du Canada	Lignes directrices quant à la prise en charge des patientes en obstétrique chez lesquelles la présence du syndrome respiratoire aigu sévère (SRAS) est soupçonnée ou probable, et des nouveau-nés issus de ces patientes	2009-avril
869	P	Société des obstétriciens et gynécologues du Canada	Prise en charge du méconium à la naissance	2009-avril
870	M	Society of Obstetricians and Gynaecologists of Canada	Management of meconium at birth	2009-Apr
871	I	Cancer Care Ontario's Program in Evidence-based Care	Intraspinal techniques for pain management in cancer patients	2009-May-5
872	T	Cancer Care Ontario's Program in Evidence-based Care	The management of head and neck cancer in Ontario: organizational and clinical practice guideline recommendations	2009-May-29
873	I	Cancer Care Ontario's Program in Evidence-based Care	Interferon-alfa in the treatment of patients with inoperable locally advanced or metastatic renal cell cancer	2009-May-12
874	A	Canadian Association of Psychosocial Oncology; Canadian Partnership Against Cancer	A Pan-Canadian clinical practice guideline: assessment of psychosocial health care needs of the adult cancer patient	2009-May
875	C	International Conference on Concussion in Sport	Consensus statement on concussion in sport: the 3rd International Conference on Concussion in Sport held in Zurich, November 2008	2009-May
876	O	Canadian Agency for Drugs and Technologies in Health	Optimal therapy recommendations for the prescribing and use of insulin analogues	2009-May
877	U	Public Health Agency of Canada	Use of antivirals to treat H1N1 flu virus (human swine flu)	2009-May
878	D	Collège des médecins du Québec	Douleur chronique et opioïdes : l'essentiel	2009-mai
879	H	Association Canadienne d'Oncologie Psychosociale; Partenariat canadien contre le cancer	Guide pancanadien de pratique clinique : évaluation des besoins en soins psychosociaux du patient adulte atteint de cancer	2009-mai
880	L	Agence de santé publique du Canada	L'utilization d'antiviraux pour combattre le virus H1N1 (grippe porcine chez l'être humain)	2009-mai

881	S	Cancer Care Ontario's Program in Evidence-based Care	Sunitinib malate for gastrointestinal stromal tumour (GIST) in imatinib mesylate resistant patients	2009-June-9
882	L	Ordre des pharmaciens du Québec; Collège des médecins du Québec	La burprénorphine dans le traitement de la dépendance aux opioïdes	2008-août (Reviewed 2009-juin)
883	C	College of Physicians and Surgeons of Ontario	Clinical practice parameters and facility standands - Hemodialysis – 3rd edition, November 2008	2008-Nov (Reviewed 2009-June)
884	C	Canadian Ophthalmological Society	Canadian Ophthalmological Society evidence-based clinical practice guidelines for the management of glaucoma in the adult eye	2009-June
885	C	Canadian Urological Association	Canadian Urological Association guidelines on the management of renal cyst disease	2009-June
886	H	Canadian Urological Association	Guidelines on prostate biopsy methodology	2009-June
887	M	Canadian Urological Association	Multicystic dysplastic kidney (MCDK) in the neonate: the role of the urologist	2009-June
888	V	Society of Obstetricians and Gynaecologists of Canada	Vaginal delivery of breech presentation	2009-June
889	S	Cancer Care Ontario's Program in Evidence-based Care	Systemic adjuvant therapy for patients at high risk for recurrent melanoma	2004-June-30 (Reviewed 2009-June-22)
890	W	Thrombosis Interest Group of Canada; Canadian Pediatric Thrombosis and Hemostasis Network	Warfarin in children	2009-June
891	A	Société des obstétriciens et gynécologues du Canada	Accouchement du siège par voie vaginale	2009-juin
892	D	Association des urologues du Canada	Dysplasie rénale multikystique (DRM) chez le nouveau-né : le rôle de l’urologue	2009-juin
893	E	Association des urologues du Canada	Exploration de l'azoospermie	2009-juin
894	H	Association des urologues du Canada	Guide de pratique canadien pour le traitement du cancer de la vessie sans envahissement musculaire	2009-juin
895	H	Société canadienne d'opthalmologie	Guide de pratique pour la prise en charge des kystes rénaux	2009-juin
896	H	Société canadienne d'opthalmologie	Guide de pratique sur la méthodologie de la biopsie prostatique	2009-juin

897	H	Société canadienne d'opthalmologie	Guide factuel de pratique clinique de la Société canadienne d'ophtalmologie pour la gestion du glaucome chez l'adulte	2009-juin
898	V	Agence de santé publique du Canada	Virus de la grippe H1N1 Lignes directrices provisoires Le 15 juin 2009	2009-juin
899	L	Agence de santé publique du Canada	Lignes directrices provisoires : Mesures de prévention et de contrôle des infections à l'intention des travailleurs de la santé dans les établissements de soins actifs	2009-avril (Reviewed 2009-juill-28)
900	N	Registered Nurses' Association of Ontario	Nursing management of hypertension	2005-Oct (Reviewed 2009-July)
901	I	Public Health Agency of Canada	Interim Guidance: Infection prevention and control measures for health care workers in acute care facilities - human cases of swine influenza A (H1N1)	2009-Apr (Reviewed 2009-July-28)
902	S	Cancer Care Ontario's Program in Evidence-based Care	Sentinel lymph node biopsy in early-stage breast cancer	2009-July-14
903	B	Canadian Association of Pathologists	Best practice recommendations for standardization of immunohistochemistry tests (page 14-25)	2009-July
904	D	Registered Nurses' Association of Ontario	Decisional support for adults living with chronic kidney disease	2009-July
905	I	Society of Gynecologic Oncologists of Canada; Society of Canadian Colposcopists; Society of Obstetricians and Gynaecologists of Canada	Initial evaluation and referral guidelines for management of pelvic/ovarian masses	2009-July
906	O	Canadian Agency for Drugs and Technologies in Health	Optimal therapy recommendations for the prescribing and use of blood glucose test strips	2009-July
907	S	Registered Nurses' Association of Ontario	Supporting clients on methadone maintenance treatment	2009-July
908	L	L'Association des infirmières et infirmiers autorisès de l'Ontario	Le soutien de la clientèle recevant des traitements d'entretien à la méthadone	2009-juill

909	P	Société des gynécologues oncologues du Canada; Société canadienne des colposcopistes; Société des obstétriciens et gynécologues du Canada	Prise en charge des masses pelviennes / ovariennes : évaluation initiale et lignes directrices quant à l'orientation des patientes	2009-juill
910	T	Cancer Care Ontario's Program in Evidence-based Care	The continued use of Trastuzumab beyond disease progression in patients with metastatic breast cancer	2009-Aug-17
911	H	Canadian Paediatric Society	Guidelines for paediatric emergency equipment and supplies for a physician's office	2009-Aug
912	H	Society of Obstetricians and Gynaecologists of Canada	Guidelines for the management of vasa previa	2009-Aug
913	M	Consensus Panel on Management of Dysfunctional Tear Syndrome	Management of dysfunctional tear syndrome: a Canadian consensus	2009-Aug
914	N	Canadian Paediatric Society	Nutrition in neurologically impaired children	2009-Aug
915	O	Registered Nurses' Association of Ontario	Ostomy care and management	2009-Aug
916	P	Society of Obstetricians and Gynaecologists of Canada	Preimplantation genetic testing	2009-Aug
917	D	Société des obstétriciens et gynécologues du Canada	Dépistage génétique préimplantatoire	2009-août
918	D	Société des obstétriciens et gynécologues du Canada	Directive clinique sur la prise en charge du vasa praevia	2009-août
919	L	Société canadienne de pédiatrie	L'alimentation des enfants ayant une déficience neurologique	2009-août
920	L	Société canadienne de pédiatrie	Les lignes directrices relatives au matériel et aux fournitures pédiatriques d'urgence au cabinet du médecin	2009-août
921	U	Canadian College of Medical Geneticists	Use of array genomic hybridization technology in constitutional genetic diagnosis in Canada	2009-Sept-22
922	A	Société des obstétriciens et gynécologues du Canada	Antibiothérapie et rupture prématurée des membranes préterme	2009-sept
923	A	Society of Obstetricians and Gynaecologists of Canada	Antibiotic therapy in preterm premature rupture of the membranes	2009-Sept

924	E	Société des obstétriciens et gynécologues du Canada	Évaluation des anomalies congénitales structurelles diagnostiquées pendant la période prénatale	2009-sept
925	E	Society of Obstetricians and Gynaecologists of Canada	Evaluation of prenatally diagnosed structural congenital anomalies	2009-Sept
926	H	Ontario Association of Medical Laboratories	Guideline for folate testing	2009-Sept
927	H	Ontario Association of Medical Laboratories	Guideline for reporting laboratory test results	2009-Sept
928	L	Société canadienne de pédiatrie	La prise en charge de l'otite moyenne aiguë	2009-sept
929	M	Canadian Paediatric Society	Management of acute otitis media	2009-Sept
930	T	Thrombosis Interest Group of Canada	TIGC guideline on the post-thrombotic syndrome	2008-Jan (Reviewed 2009-Oct)
931	0-9	Canadian Cardiovascular Society	2009 Canadian Cardiovascular Society/Canadian guidelines for the diagnosis and treatment of dyslipidemia and prevention of cardiovascular disease in the adult – 2009 recommendations	2009-Oct
932	A	Society of Obstetricians and Gynaecologists of Canada	Active management of the third stage of labour: Prevention and treatment of postpartum hemorrhage	2009-Oct
933	C	Canadian Network for Mood and Anxiety Treatments	Canadian Network for Mood and Anxiety Treatments (CANMAT) clinical guidelines for the management of major depressive disorder in adults	2009-Oct
934	C	Canadian Network for Mood and Anxiety Treatments	Canadian Network for Mood and Anxiety Treatments (CANMAT) clinical guidelines for the management of major depressive disorder in adults. I. Classification, burden and principles of management	2009-Oct
935	C	Canadian Network for Mood and Anxiety Treatments	Canadian Network for Mood and Anxiety Treatments (CANMAT) clinical guidelines for the management of major depressive disorder in adults. II. Psychotherapy alone or in combination with antidepressant medication	2009-Oct
936	C	Canadian Network for Mood and Anxiety Treatments	Canadian Network for Mood and Anxiety Treatments (CANMAT) clinical guidelines for the management of major depressive disorder in adults. III. Pharmacotherapy	2009-Oct

937	C	Canadian Network for Mood and Anxiety Treatments	Canadian Network for Mood and Anxiety Treatments (CANMAT) clinical guidelines for the management of major depressive disorder in adults. IV. Neurostimulation therapies	2009-Oct
938	C	Canadian Network for Mood and Anxiety Treatments	Canadian Network for Mood and Anxiety Treatments (CANMAT) clinical guidelines for the management of major depressive disorder in adults. V. Complementary and alternative medicine treatments	2009-Oct
939	E	Thrombosis Interest Group of Canada	Establishing therapeutic range for heparin	2009-Oct
940	L	Société canadienne de cardiologie	Lignes directrices 2009 de la Société canadienne de cardiologie pour diagnostiquer et traiter la dyslipidémie et prévenir la maladie cardiovasculaire chez l'adulte - Recommandations de 2009	2009-oct
941	U	Collège des médecins du Québec	Utilisation de la sédation-analgésie	2009-oct
942	T	Cancer Care Ontario's Program in Evidence-based Care	The delivery of brachytherapy for cervical cancer: Organizational and technical advice to facilitate high-quality care in Ontario	2009-Nov-11
943	S	Alberta Health Services - Cancer Care	Sentinel lymph node biopsy and breast conservation surgery	2007-Jan (Reviewed 2009-Nov)
944	E	Alberta Health Services - Cancer Care	Ependymomas	2009-Nov
945	E	Canadian Paediatric Society	Extended-release medications for children and adolescents with attention-deficit hyperactivity disorder	2009-Nov
946	L	Société canadienne de pédiatrie	Les médicaments à libération prolongée pour les enfants et les adolescents ayant un trouble de déficit de l'attention avec hyperactivité	2009-nov
947	L	Société canadienne de cardiologie; Académie canadienne de psychiatrie de l'enfant et l'adolescent; Société canadienne de pédiatrie	L'évaluation du risque cardiaque avant l'utilisation de stimulants chez les enfants et les adolescents	2009-nov
948	O	Guidelines and Protocols Advisory Committee (BC)	Osteoarthritis in peripheral joints - diagnosis and treatment	2008-Sept-15 (Reviewed 2009-Dec-1)

949	D	Guidelines and Protocols Advisory Committee (BC)	Dyspepsia with or without helicobacter pylori infection - Clinical approach in adults	2009-Dec-1
950	S	Société canadienne du rétinoblastome	Stratégie thérapeutique du rétinoblastome guide clinique canadien	2009-déc
951	D	Canadian Cardiovascular Society	Dual antiplatelet therapy in patients requiring urgent coronary artery bypass grafting surgery: A position statement of the Canadian Cardiovascular Society	2009-Dec
952	N	Canadian Retinoblastoma Society	National retinoblastoma strategy Canadian guidelines for care	2009-Dec
953	O	Alberta Health Services - Cancer Care	Optimal use of taxanes in metastatic breast cancer	2007-Dec (Reviewed 2009-Dec)
954	C	Canadian Urological Association	Canadian guidelines for the management of asymptomatic microscopic hematuria in adults	2008
955	C	Champlain Cardiovascular Disease (CVD) Prevention Network	Champlain primary care cardiovascular disease prevention and management guideline	2008
956	C	Canadian Dental Hygienists Association	Current issues in infection control practices in dental hygiene - Part 2	2008
957	E	Canadian Academy of Sport and Exercise Medicine	Exercise and pregnancy	2008
958	H	Canadian Society of Nephrology	Guidelines for the management of chronic kidney disease	2008
959	I	Canadian Dental Hygienists Association	Infection control practice guidelines in dental hygiene - Part 1	2008
960	L	Association des urologues du Canada	Lignes directrices canadiennes pour la prise en charge de l'hématurie microscopique asymptomatique chez les adultes	2008
961	P	Registered Nurses' Association of Ontario	Promoting asthma control in children - Supplement 2008	2008
962	R	Association pulmonaire du Canada; Société canadienne de thoracologie	Recommandations pour le diagnostic et le traitement de l'apnée du sommeil : mise à jour de 2008	2008
963	R	Canadian Lung Association; Canadian Thoracic Society	Recommendations for the diagnosis and treatment of sleep apnea - 2008 update	2008
964	S	Alberta Cancer Board; Toward Optimized Practice [Alberta]	Screening for colorectal cancer	2008

965	A	Registered Nurses' Association of Ontario	Assessment and device selection for vascular access	2004-May (Reviewed 2008)
966	A	Registered Nurses' Association of Ontario	Assessment and device selection for vascular access - Supplement 2008	2004-May (Reviewed 2008)
967	P	Registered Nurses' Association of Ontario	Promoting asthma control in children	2004-May (Reviewed 2008)
968	C	Registered Nurses' Association of Ontario	Care and maintenance to reduce vascular access complications	2005-Apr (Reviewed 2008)
969	H	Toward Optimized Practice [Alberta]	Guideline for the diagnosis and management of community acquired pneumonia : pediatric	2002-Feb (Reviewed 2008-Jan)
970	H	Toward Optimized Practice [Alberta]	Guideline for the diagnosis and management of community acquired pneumonia: Adult	2002-Feb (Reviewed 2008-Jan)
971	H	Toward Optimized Practice [Alberta]	Guideline for the diagnosis and management of acute bacterial sinusitis	2006-Jan (Reviewed 2008-Jan)
972	H	Toward Optimized Practice [Alberta]	Guideline for the use of prenatal ultrasound : first trimester	2005-Jan (Reviewed 2008-Jan)
973	H	Toward Optimized Practice [Alberta]	Guideline for ultrasound as part of routine prenatal care	2005-Jan (Reviewed 2008-Jan)
974	H	Toward Optimized Practice [Alberta]	Guideline for the diagnosis and management of acute otitis media in children	2007 (Reviewed 2008-Jan)
975	H	Toward Optimized Practice [Alberta]	Guideline for the diagnosis and management of croup	2007 (Reviewed 2008-Jan)
976	H	Toward Optimized Practice [Alberta]	Guideline for the diagnosis and management of nursing home acquired pneumonia (NHAP)	2007-Mar (Reviewed 2008-Jan)
977	L	Agence de santé publique du Canada	Lignes directrices canadiennes sur les infections transmissibles sexuellement : abus sexuel à l'égard d'enfants impubères et prépubères	2006 (Reviewed 2008-janv)
978	L	Agence de santé publique du Canada	Lignes directrices canadiennes sur les infections transmissibles sexuellement : Agression sexuelle chez les adolescents postpubères et chez les adultes	2006 (Reviewed 2008-janv)
979	L	Agence de santé publique du Canada	Lignes directrices canadiennes sur les infections transmissibles sexuellement : Atteintes inflammatoires pelviennes (AIP)	2006 (Reviewed 2008-janv)
980	L	Agence de santé publique du Canada	Lignes directrices canadiennes sur les infections transmissibles sexuellement : Chancre mou	2006 (Reviewed 2008-janv)

981	L	Agence de santé publique du Canada	Lignes directrices canadiennes sur les infections transmissibles sexuellement : Consommation de substances psychoactives	2006 (Reviewed 2008-janv)
982	L	Agence de santé publique du Canada	Lignes directrices canadiennes sur les infections transmissibles sexuellement : Détenus et délinquants	2006 (Reviewed 2008-janv)
983	L	Agence de santé publique du Canada	Lignes directrices canadiennes sur les infections transmissibles sexuellement : diagnostic en laboratoire des infections transmissibles sexuellement	2006 (Reviewed 2008-janv)
984	L	Agence de santé publique du Canada	Lignes directrices canadiennes sur les infections transmissibles sexuellement : Épididymite	2006 (Reviewed 2008-janv)
985	L	Agence de santé publique du Canada	Lignes directrices canadiennes sur les infections transmissibles sexuellement : Femmes enceintes	2006 (Reviewed 2008-janv)
986	L	Agence de santé publique du Canada	Lignes directrices canadiennes sur les infections transmissibles sexuellement : Hommes ayant des relations sexuelles avec d'autres hommes et femmes ayant des relations sexuelles avec d'autres femmes	2006 (Reviewed 2008-janv)
987	L	Agence de santé publique du Canada	Lignes directrices canadiennes sur les infections transmissibles sexuellement : Immigrants et réfugiés	2006 (Reviewed 2008-janv)
988	L	Agence de santé publique du Canada	Lignes directrices canadiennes sur les infections transmissibles sexuellement : Infections à Chlamydia	2006 (Reviewed 2008-janv)
989	L	Agence de santé publique du Canada	Lignes directrices canadiennes sur les infections transmissibles sexuellement : Infections au virus de l'hépatite B	2006 (Reviewed 2008-janv)
990	L	Agence de santé publique du Canada	Lignes directrices canadiennes sur les infections transmissibles sexuellement : Infections au virus de l'immunodéficience humaine	2006 (Reviewed 2008-janv)
991	L	Agence de santé publique du Canada	Lignes directrices canadiennes sur les infections transmissibles sexuellement : Infections génitales au virus du papillome humain (VPH)	2006 (Reviewed 2008-janv)

992	L	Agence de santé publique du Canada	Lignes directrices canadiennes sur les infections transmissibles sexuellement : Infections génitales au virus herpes simplex (VHS)	2006 (Reviewed 2008-janv)
993	L	Agence de santé publique du Canada	Lignes directrices canadiennes sur les infections transmissibles sexuellement : Infections intestinales et entériques transmissibles sexuellement	2006 (Reviewed 2008-janv)
994	L	Agence de santé publique du Canada	Lignes directrices canadiennes sur les infections transmissibles sexuellement : Infestations ectoparasitaires (pédiculose pubienne, gale)	2006 (Reviewed 2008-janv)
995	L	Agence de santé publique du Canada	Lignes directrices canadiennes sur les infections transmissibles sexuellement : Les travailleurs de l'industrie du sexe	2006 (Reviewed 2008-janv)
996	L	Agence de santé publique du Canada	Lignes directrices canadiennes sur les infections transmissibles sexuellement : Lymphogranulomatose vénérienne (LGV)	2006 (Reviewed 2008-janv)
997	L	Agence de santé publique du Canada	Lignes directrices canadiennes sur les infections transmissibles sexuellement : Pertes vaginales (vaginose bactérienne, candidose vulvo-vaginale, trichomonase)	2006 (Reviewed 2008-janv)
998	L	Agence de santé publique du Canada	Lignes directrices canadiennes sur les infections transmissibles sexuellement : Prise en charge des ITS en fonction du syndrome présenté	2006 (Reviewed 2008-janv)
999	L	Agence de santé publique du Canada	Lignes directrices canadiennes sur les infections transmissibles sexuellement : Prostatite	2006 (Reviewed 2008-janv)
1000	L	Agence de santé publique du Canada	Lignes directrices canadiennes sur les infections transmissibles sexuellement : Soins primaires et infections transmissibles sexuellement	2006 (Reviewed 2008-janv)
1001	L	Agence de santé publique du Canada	Lignes directrices canadiennes sur les infections transmissibles sexuellement : Ulcérations génitales (UG)	2006 (Reviewed 2008-janv)
1002	L	Agence de santé publique du Canada	Lignes directrices canadiennes sur les infections transmissibles sexuellement : Urétrite	2006 (Reviewed 2008-janv)

1003	L	Agence de santé publique du Canada	Lignes directrices canadiennes sur les infections transmissibles sexuellement : Voyageurs	2006 (Reviewed 2008-janv)
1004	C	Public Health Agency of Canada	Canadian guidelines on sexually transmitted infections : Chancroid	2006 (Reviewed 2008-Jan)
1005	C	Public Health Agency of Canada	Canadian guidelines on sexually transmitted infections : Chlamydial infections	2006 (Reviewed 2008-Jan)
1006	C	Public Health Agency of Canada	Canadian guidelines on sexually transmitted infections : Ectoparasitic infestations (pubic lice, scabies)	2006 (Reviewed 2008-Jan)
1007	C	Public Health Agency of Canada	Canadian guidelines on sexually transmitted infections : Epididymitis	2006 (Reviewed 2008-Jan)
1008	C	Public Health Agency of Canada	Canadian guidelines on sexually transmitted infections : Genital herpes simplex virus (HSV) infections	2006 (Reviewed 2008-Jan)
1009	C	Public Health Agency of Canada	Canadian guidelines on sexually transmitted infections : Genital human papillomavirus (HPV) infections	2006 (Reviewed 2008-Jan)
1010	C	Public Health Agency of Canada	Canadian guidelines on sexually transmitted infections : Genital ulcer disease (GUD)	2006 (Reviewed 2008-Jan)
1011	C	Public Health Agency of Canada	Canadian guidelines on sexually transmitted infections : Hepatitis B virus infections	2006 (Reviewed 2008-Jan)
1012	C	Public Health Agency of Canada	Canadian guidelines on sexually transmitted infections : Human immunodeficiency virus (HIV) infections	2006 (Reviewed 2008-Jan)
1013	C	Public Health Agency of Canada	Canadian guidelines on sexually transmitted infections : Immigrants and refugees	2006 (Reviewed 2008-Jan)
1014	C	Public Health Agency of Canada	Canadian guidelines on sexually transmitted infections : Inmates and offenders	2006 (Reviewed 2008-Jan)
1015	C	Public Health Agency of Canada	Canadian guidelines on sexually transmitted infections : Laboratory diagnosis of sexually transmitted infections	2006 (Reviewed 2008-Jan)
1016	C	Public Health Agency of Canada	Canadian guidelines on sexually transmitted infections : Lymphogranuloma venereum (LGV)	2006 (Reviewed 2008-Jan)
1017	C	Public Health Agency of Canada	Canadian guidelines on sexually transmitted infections : Men who have sex with men (MSM)/ women who have sex with women (WSW)	2006 (Reviewed 2008-Jan)

1018	C	Public Health Agency of Canada	Canadian guidelines on sexually transmitted infections : Pelvic inflammatory disease (PID)	2006 (Reviewed 2008-Jan)
1019	C	Public Health Agency of Canada	Canadian guidelines on sexually transmitted infections : Pregnancy	2006 (Reviewed 2008-Jan)
1020	C	Public Health Agency of Canada	Canadian guidelines on sexually transmitted infections : Primary care and sexually transmitted infections	2006 (Reviewed 2008-Jan)
1021	C	Public Health Agency of Canada	Canadian guidelines on sexually transmitted infections : Prostatitis	2006 (Reviewed 2008-Jan)
1022	C	Public Health Agency of Canada	Canadian guidelines on sexually transmitted infections : Sex workers	2006 (Reviewed 2008-Jan)
1023	C	Public Health Agency of Canada	Canadian guidelines on sexually transmitted infections : sexual abuse in peripubertal and prepubertal children	2006 (Reviewed 2008-Jan)
1024	C	Public Health Agency of Canada	Canadian guidelines on sexually transmitted infections : Sexual assault in postpubertal adolescents and adults	2006 (Reviewed 2008-Jan)
1025	C	Public Health Agency of Canada	Canadian guidelines on sexually transmitted infections : Sexually transmitted intestinal and enteric infections	2006 (Reviewed 2008-Jan)
1026	C	Public Health Agency of Canada	Canadian guidelines on sexually transmitted infections : Substance use	2006 (Reviewed 2008-Jan)
1027	C	Public Health Agency of Canada	Canadian guidelines on sexually transmitted infections : Syndromic management of sexually transmitted infections	2006 (Reviewed 2008-Jan)
1028	C	Public Health Agency of Canada	Canadian guidelines on sexually transmitted infections : Travellers	2006 (Reviewed 2008-Jan)
1029	C	Public Health Agency of Canada	Canadian guidelines on sexually transmitted infections : Urethritis	2006 (Reviewed 2008-Jan)
1030	C	Public Health Agency of Canada	Canadian guidelines on sexually transmitted infections : Vaginal discharge (bacterial vaginosis, vulvovaginal candidiasis, trichomoniasis)	2006 (Reviewed 2008-Jan)
1031	H	Toward Optimized Practice [Alberta]	Guideline for the diagnosis and treatment of acute pharyngitis	2006 (Reviewed 2008-Jan)
1032	H	Toward Optimized Practice [Alberta]	Guideline for the management of acute bronchitis	2006 (Reviewed 2008-Jan)
1033	L	Toward Optimized Practice [Alberta]	Laboratory endocrine testing guidelines : Acromegaly	2006 (Reviewed 2008-Jan)

1034	L	Toward Optimized Practice [Alberta]	Laboratory endocrine testing guidelines : Adrenal Insufficiency (Addison's Disease)	2006 (Reviewed 2008-Jan)
1035	L	Toward Optimized Practice [Alberta]	Laboratory endocrine testing guidelines : Cushing's syndrome	2006 (Reviewed 2008-Jan)
1036	L	Toward Optimized Practice [Alberta]	Laboratory endocrine testing guidelines : Galactorrhea	2006 (Reviewed 2008-Jan)
1037	L	Toward Optimized Practice [Alberta]	Laboratory endocrine testing guidelines : Hypercalcemia	2006 (Reviewed 2008-Jan)
1038	L	Toward Optimized Practice [Alberta]	Laboratory endocrine testing guidelines : Hypoglycemia	2006 (Reviewed 2008-Jan)
1039	L	Toward Optimized Practice [Alberta]	Laboratory endocrine testing guidelines : Pheochromocytoma	2006 (Reviewed 2008-Jan)
1040	L	Toward Optimized Practice [Alberta]	Laboratory endocrine testing guidelines: Gonadal disorders : hirsutism	2006 (Reviewed 2008-Jan)
1041	U	Société des obstétriciens et gynécologues du Canada	Utilisation de progestérone pour la prévention de l'accouchement prématuré	2008-janv
1042	M	Cancer Care Ontario's Program in Evidence-based Care	Management of stage I seminoma : Guideline recommendations	2008-Jan-30
1043	D	Canadian Consensus Conference on the Diagnosis and Treatment of Dementia	Diagnosis and treatment of dementia : introduction : introducing a series based on the Third Canadian Consensus Conference on the Diagnosis and Treatment of Dementia	2008-Jan-29
1044	T	Cancer Care Ontario's Program in Evidence-based Care	The management of malignant thrombocytosis in Philadelphia chromosome-negative myeloproliferative disease : Guideline recommendations	2008-Jan-15
1045	C	Canadian Cardiovascular Society	Canadian Cardiovascular Society consensus conference guidelines on heart failure - 2008 update: Best practices for the transition of care of heart failure patients, and the recognition, investigation and treatment of cardiomyopathies	2008-Jan
1046	C	Association of Medical Microbiology and Infectious Disease Canada; Canadian Thoracic Society	Clinical practice guidelines for hospital-acquired pneumonia and ventilator-associated pneumonia in adults	2008-Jan
1047	T	Society of Obstetricians and Gynaecologists of Canada	The use of progesterone for prevention of preterm birth	2008-Jan

1048	L	Société canadienne de pédiatrie	Lignes directrices multidisciplinaires sur la determination, l'enquête et la prise en charge des cas presumes de traumatisme crânien non accidental	2008-fév
1049	T	Toward Optimized Practice [Alberta]	The medical induction of labour	2006-Jan (Reviewed 2008-Feb)
1050	R	Société canadienne de thoracologie	Recommandations de la Société canadienne de thoracologie au sujet de la prise en charge de la maladie pulmonaire obstructive chronique - mise à jour de 2008 - points saillants pour les soins primaires	2008-fév
1051	R	Société des obstétriciens et gynécologues du Canada	Rubéole au cours de la grossesse	2008-fév
1052	D	Canadian Consensus Conference on the Diagnosis and Treatment of Dementia	Diagnosis and treatment of dementia : 1. Risk assessment and primary prevention of Alzheimer disease	2008-Feb-26
1053	M	Cancer Care Ontario's Program in Evidence-based Care	Management of stage I nonseminomatous testicular cancer	2008-Feb-14
1054	C	Canadian Thoracic Society	Canadian Thoracic Society recommendations for the management of chronic obstructive pulmonary disease - 2008 update - highlights for primary care	2008-Feb
1055	C	Saskatchewan Health	Clinical practice guidelines for the prevention and management of diabetes foot complications	2008-Feb
1056	M	Canadian Paediatric Society	Multidisciplinary guidelines on the identification, investigation and management of suspected abusive head trauma	2008-Feb
1057	T	Cancer Care Ontario's Program in Evidence-based Care	The role of aromatase inhibitors in adjuvant therapy for postmenopausal women with hormone receptor-positive breast cancer: a clinical practice guideline	2005-Oct-25 (Reviewed 2008-Feb-26)
1058	R	Society of Obstetricians and Gynaecologists of Canada	Rubella in pregnancy	2008-Feb
1059	L	Toward Optimized Practice [Alberta]	Laboratory endocrine testing guidelines: Amenorrhea (without hirsutism) and menopause	2006 (Reviewed 2008-Mar)

1060	R	Association canadienne des médecins d'urgence	Révision des lignes directrices de l'échelle canadienne de triage et de gravité (ÉTG) pour les adultes	2008-mars
1061	D	Canadian Consensus Conference on the Diagnosis and Treatment of Dementia	Diagnosis and treatment of dementia : 2. Diagnosis	2008-Mar-25
1062	P	Cancer Care Ontario's Program in Evidence-based Care	Provider-patient communication : a report of evidence-based recommendations to guide practice in cancer	2008-Mar-17
1063	I	Toward Optimized Practice [Alberta]	Investigation and management of primary thyroid dysfunction	2007 (Reviewed 2008-Mar)
1064	D	Society of Obstetricians and Gynaecologists of Canada	Diagnosis, evaluation and management of the hypertensive disorders of pregnancy	2008-Mar
1065	D	Society of Obstetricians and Gynaecologists of Canada	Diagnosis, evaluation and management of the hypertensive disorders of pregnancy - Chapter 1: Diagnosis and classification	2008-Mar
1066	D	Society of Obstetricians and Gynaecologists of Canad	Diagnosis, evaluation and management of the hypertensive disorders of pregnancy - Chapter 2: Prediction, prevention, and prognosis of preeclampsia	2008-Mar
1067	D	Society of Obstetricians and Gynaecologists of Canada	Diagnosis, evaluation and management of the hypertensive disorders of pregnancy - Chapter 3: Treatment of the hypertensive disorders of pregnancy	2008-Mar
1068	H	BC Oral Cancer Prevention Program; College of Dental Surgeons of British Columbia	Guideline for the early detection of oral cancer in British Columbia	2008-Mar
1069	R	Canadian Association of Emergency Physicians	Revisions to the Canadian emergency department triage and acuity scale (CTAS) adult guidelines	2008-Mar
1070	L	Société canadienne de pédiatrie	Le transport des nourrissons et des enfants dans les véhicules automobiles	2008-avril
1071	M	Society of Obstetricians and Gynaecologists of Canada	Midurethral minimally invasive sling procedures for stress urinary incontinence	2008-Aug
1072	S	Society of Obstetricians and Gynaecologists of Canada	Screening and management of bacterial vaginosis in pregnancy	2008-Aug

1073	S	Society of Obstetricians and Gynaecologists of Canada	SOGC committee opinion on urodynamics testing	2008-Aug
1074	A	Cancer Care Ontario's Program in Evidence-based Care	Adjuvant systemic chemotherapy for stage II and III colon cancer following complete resection	2008-Apr-17
1075	O	Cancer Care Ontario's Program in Evidence-based Care	Optimization of surgical and pathological quality performance in radical surgery for colon and rectal cancer: margins and lymph nodes guideline recommendations	2008-Apr-17
1076	A	Genito-Urinary Radiation Oncologists of Canada	Adjuvant radiotherapy following radical prostatectomy: Genito-Urinary Radiation Oncologists of Canada Consensus Statement	2008-Apr
1077	B	Consensus Panel on Management of Patients with Blepharitis	Blepharitis : current strategies for diagnosis and management	2008-Apr
1078	C	Registered Nurses' Association of Ontario	Care and mintenance to reduce vascular access complications - Supplement 2008	2008-Apr
1079	C	College of Physicians and Surgeons of Ontario	Clinical Practice Parameters and Facility Standars - Pulmonary function studies - 3rd edition, April 2008	2008-Apr
1080	C	College of Physicians and Surgeons of Ontario	Clinical Pratice Parameters and Facility Standards - Diagnostic spirometry & flow volume loop studies - 3rd edition, April 2008	2008-Apr
1081	H	Society of Obstetricians and Gynaecologists of Canada	Genital herpes: gynaecological aspects	2008-Apr
1082	T	Canadian Paediatric Society	Transportation of infants and children in motor vehicles	2008-Apr
1083	D	Canadian Consensus Conference on the Diagnosis and Treatment of Dementia	Diagnosis and treatment of dementia : 3. Mild cognitive impairment and cognitive impairment without dementia	2008-May-6
1084	R	Canadian Association of Emergency Physicians	Revisions to the Canadian triage and acuity scale paediatric guidelines (PaedCTAS)	2008-May
1085	R	Association canadienne des médecins d'urgence	Révision des lignes directrices de l'échelle canadienne de triage et de gravité (ÉTG) applicable aux enfants	2008-mai
1086	P	Cancer Care Ontario's Program in Evidence-based Care	Preoperative or postoperative therapy for resectable esophageal cancer : guideline recommendations	2005-Apr-13 (Reviewed 2008-May-21)

1087	T	Cancer Care Ontario's Program in Evidence-based Care	The role of Bevacizumab (Avastin®) combined with chemotherapy in the treatment of patients with advanced colorectal cancer : a clinical practice guideline	2005-Dec-12 (Reviewed 2008-May-28)
1088	D	Association des urologues du Canada	Dépistage et prise en charge d'une hydronéphrose anténatale	2008-juin
1089	D	Société des obstétriciens et gynécologues du Canada	Directive clinique sur la prise en charge du virus de l'herpès simplex pendant la grossesse	2008-juin
1090	L	Association des urologues du Canada	Lignes directrices pour le suivi après une néphrectomie radicale ou partielle en raison d'un hypernéphrome localisé de stade avancé	2008-juin
1091	F	Canadian Urological Association	Follow-up guidelines after radical or partial nephrectomy for localized and locally advanced renal cell carcinoma	2008-June
1092	H	Ontario Association of Medical Laboratories	Guideline for ordering serum levels of gonadotropins (FSH, LH) and prolactin	2008-June
1093	H	Provincial Blood Coordinating Office (BC)	Guidelines for blood component substitution for adult patients during an inventory shortage - adult patients	2008-June
1094	H	Society of Obstetricians and Gynaecologists of Canada	Guidelines for the management of herpes simplex virus in pregnancy	2008-June
1095	I	Canadian Interventional Radiology Association	Interdisciplinary Canadian guidelines on the use of metal stents in the gastrointestinal tract for oncological indications	2008-June
1096	I	Canadian Urological Association	Investigation and management of antenatally detected hydronephrosis	2008-June
1097	M	Canadian Kidney Cancer Forum	Management of kidney cancer: Canadian Kidney Cancer Forum Consensus Statement	2008-June
1098	P	Alberta Health and Wellness	Prenatal HIV: public health guidelines for the management and follow-up of HIV positive pregnant women and their infants	2008-June
1099	P	Cancer Care Ontario's Program in Evidence-based Care	Preoperative or postoperative therapy for the management of patients with stage II or III rectal cancer	2008-July-15

1100	C	Canadian Association of Oral & Maxillofacial Surgeons	Canadian consensus practice guidelines for bisphosphonate associated osteonecrosis of the jaw	2008-July
1101	H	Society of Obstetricians and Gynaecologists of Canada	Guideline for the management of postoperative nausea and vomiting	2008-July
1102	N	Society of Obstetricians and Gynaecologists of Canada	New molecular techniques for the prenatal detection of chromosomal aneuploidy	2008-July
1103	S	Public Health Agency of Canada	Statement on influenza vaccination for the 2008-2009 season	2008-July
1104	D	Agence de santé publique du Canada	Déclaration sur la vaccination antigrippale pour la saison 2008-2009	2008-juill
1105	D	Société des obstétriciens et gynécologues du Canada	Directive clinique sur la prise en charge des nausées et des vomissements postopératoires	2008-juill
1106	N	Société des obstétriciens et gynécologues du Canada	Nouvelles techniques moléculaires de dépistage prénatal de l'aneuploïdie chromosomique	2008-juill
1107	D	Société des obstétriciens et gynécologues du Canada	Dépistage et prise en charge de la vaginose bactérienne pendant la grossesse	2008-août
1108	I	Société des obstétriciens et gynécologues du Canada	Interventions de fronde mi-urétrales à effraction minimale visant à contrer l'incontinence urinaire à l’effort	2008-août
1109	O	Société des obstétriciens et gynécologues du Canada	Opinion de comité de la SOGC sur l?épreuve urodynamique	2008-août
1110	L	Ontario Agency for Health Protection and Promotion	Listeria monocytogenes : a clinical practice guideline	2008-Sept-3
1111	F	Cancer Care Ontario's Program in Evidence-based Care	Fulvestrant for systemic therapy of locally advanced or metastatic breast cancer in postmenopausal women	2008-Sept-25
1112	C	Guidelines and Protocols Advisory Committee (BC)	Chronic kidney disease - identification, evaluation and management of patients	2008-Sept-15
1113	C	Canadian Association of Emergency Physicians	Canadian Association of Emergency Physicians Sepsis Guidelines: the optimal management of severe sepsis in Canadian emergency departments	2008-Sept
1114	D	Collège canadien de généticiens médicaux; Société des obstétriciens et gynécologues du Canada	Dépistage du X fragile en obstétrique-gynécologie au Canada	2008-sept

1115	D	Société des obstétriciens et gynécologues du Canada	Directive clinique sur la prise en charge de la grossesse entre la 41e+0 et la 42e+0 semaine de gestation	2008-sept
1116	F	Canadian College of Medical Geneticists; Society of Obstetricians and Gynaecologists of Canada	Fragile X testing in obstetrics and gynaecology in Canada	2008-Sept
1117	H	Ontario Association of Medical Laboratories	Guideline for the laboratory monitoring of oral anticoagulation (Warfarin)	2008-Sept
1118	H	Society of Obstetricians and Gynaecologists of Canada	Guidelines for the management of pregnancy at 41+0 to 42+0 weeks	2008-Sept
1119	R	Agence de santé publique du Canada	Recommendations aux professionnels de la santé : listériose : lignes directrices canadiennes sur le diagnostic et la prise en charge des cas de listériose	2008-sept
1120	R	Public Health Agency of Canada	Recommendations to health care professionals : listeriosis : consensus Canadian guidelines for diagnosis and management of listeriosis	2008-Sept
1121	T	Cancer Care Ontario's Program in Evidence-based Care	The management of thymoma: Guideline recommendations	2008-Sept
1122	D	Canadian Consensus Conference on the Diagnosis and Treatment of Dementia	Diagnosis and treatment of dementia : 4. Approach to management of mild to moderate dementia	2008-Oct-7
1123	C	Canadian Ophthalmological Society	Canadian Ophthalmological Society evidence-based clinical practice guidelines for cataract surgery in the adult eye	2008-Oct
1124	C	Canadian College of Medical Geneticists; Society of Obstetricians and Gynaecologists of Canada	Carrier screening for Thalassemia and hemoglobinopathies in Canada	2008-Oct
1125	C	Société des obstétriciens et gynécologues du Canada	Complications obstétricales associées aux analytes anormaux des marqueurs sériques maternels	2008-oct
1126	D	Collège canadien de généticiens médicaux; Société des obstétriciens et gynécologues du Canada	Dépistage des porteurs de thalassémie et d'hémoglobinopathies au Canada	2008-oct
1127	F	Guidelines and Protocols Advisory Committee (BC)	Frailty in older adults – early identification and management	2008-Oct

1128	H	Société canadienne d'opthalmologie	Guide de pratique clinique factuelle de la Société canadienne d'ophtalmologie pour la chirurgie de la cataracte de l'œil adulte	2008-oct
1129	O	Society of Obstetricians and Gynaecologists of Canada	Obstetrical complications associated with abnormal maternal serum markers analytes	2008-Oct
1130	S	Saskatchewan Cancer Agency	SCA clinical practice guideline for prostate cancer	2008-Oct
1131	U	Public Health Agency of Canada	Updated recommendations on interferon gamma release assays for latent tuberculosis infection	2008-Oct
1132	D	Canadian Consensus Conference on the Diagnosis and Treatment of Dementia	Diagnosis and treatment of dementia : 5. Nonpharmacologic and pharmacologic therapy for mild to moderate dementia	2008-Nov-4
1133	A	Guidelines and Protocols Advisory Committee (BC)	Acute chest pain - evaluation and triage	2008-Nov
1134	A	Canadian Paediatric Society	Advance care planning for paediatric patients	2008-Nov
1135	C	Regina Qu'Appelle Health Region	Clinical practice guidelines for the prevention and management of diabetes foot complications	2008-Nov
1136	L	Société canadienne de pédiatrie	La planification préalable des soins pour les patients en pédiatrie	2008-nov
1137	L	Association canadienne des médecins d'urgence	Lignes directrices de l'Association canadienne des médecins d'urgence sur le sepsis : la prise en charge optimale du sepsis grave dans les départements d'urgence canadiens	2008-nov
1138	M	Thrombosis Interest Group of Canada	Managing warfarin associated coagulopathy	2008-Nov
1139	M	Society of Obstetricians and Gynaecologists of Canada	Missed hormonal contraceptives: new recommendations	2008-Nov
1140	O	Société des obstétriciens et gynécologues du Canada	Oubli de doses de contraceptif hormonal: nouvelles recommandations	2008-nov
1141	P	Thrombosis Interest Group of Canada	Prevention of deep vein thrombosis in medical patients	2008-Nov
1142	D	Canadian Consensus Conference on the Diagnosis and Treatment of Dementia	Diagnosis and treatment of dementia: 6. Management of severe Alzheimer disease	2008-Dec-2

1143	T	Cancer Care Ontario's Program in Evidence-based Care	Treatment of acute myeloid leukemia in older patients: guideline recommendations	2008-Dec-18
1144	A	Thrombosis Interest Group of Canada	Antiplatelet therapy	2004-Nov (Reviewed 2008-Dec)
1145	P	Thrombosis Interest Group of Canada	Post myocardial infarction	2005-Aug (Reviewed 2008-Dec)
1146	D	Collège des médecins de famille du Canada; L'Association des infirmières en santé des femmes, en obstétrique et en néonatalogie au Canada; Association canadienne des sages-femmes; Société de la médecine rurale du Canada; Société des obstétriciens et gynécologues du Canada	Déclaration de principe commune sur l'accouchement normal	2008-déc
1147	C	Canadian Association of Radiologists	Canadian Association of Radiologists National Advisory on gadolinium administration and nephrogenic systemic fibrosis	2008-Dec
1148	J	College of Family Physicians of Canada; The Association of Women's Health, Obstetric and Neonatal Nurses Canada; Canadian Association of Midwives; Society of Rural Physicians of Canada; Society of Obstetricians and Gynaecologists of Canada	Joint policy statement on normal childbirth	2008-Dec
1149	O	Registered Nurses' Association of Ontario	Oral health: Nursing assessment and interventions	2008-Dec
1150	S	Thrombosis Interest Group of Canada	Stroke prevention in atrial fibrillation	2008-Dec
