# Supplementary material for: Reporting of financial conflicts of interest in clinical practice guidelines: a case study analysis of guidelines from the Canadian Medical Association Infobase
Source: BMC Health Serv Res. 2016 Aug 15;16:383. doi: 10.1186/s12913-016-1646-5 (PMC4986411; doi:10.1186/s12913-016-1646-5)
Supplement: Additional file 3: — Guideline-level data on recommended drugs and guideline-affiliated organizations. This table provides the recommended drugs in each guideline included in our study, the manufacturers of the brand name drugs recommended in these guidelines, the organizations that were indicated to be affiliated with each guideline, and the pharmaceutical companies with which these organizations identified relationships. (PDF 264 kb) [file 12913_2016_1646_MOESM3_ESM.pdf]

Appendix 3: Guideline-level data on recommended drugs and guideline-affiliated organizations

Clinical practice guideline ID #	Most recent review or publish date	CPS year	Clinical practice guideline title	Drugs recommended for first-line treatment	Brand companies identified in CPS or whether generic versions are available	Name of recommended drug identified in CPS or brand name to which generic name redirects	Guideline-affiliated organizations and [source] of identified relationship with pharmaceutical companies	Identified pharmaceutical companies with relationship with organizations[1]
5	2013	2013	The 2012-2013 Canadian Hypertension Education Program (CHEP) Guidelines for Pharmacists: An Update	spironolactone	generic versions		EPICORE Centre/ COMPRIS [1]	- Apotex Inc.<br>- AstraZeneca Canada<br>- Bayer Healthcare Pharmaceuticals<br>- Bristol-Myers Squibb/Sanofi-Aventis<br>- Merck Frosst Canada
				eplerenone	Pfizer	Inspira		
7	2013	2013	Canadian Network for Mood and Anxiety Treatments (CANMAT) and International Society for Bipolar Disorders (ISBD) Collaborative Update of CANMAT Guidelines for the Management of Patients with Bipolar Disorder: 2009	lithium	generic versions		Canadian Network for Mood and Anxiety Treatments (CANMAT) [2]<br><br>International Society for Bipolar Disorders (ISBD) [3]	CANMAT<br>- None identified<br><br>ISBD<br>- Janssen Pharmaceutical Companies<br>- Forest Pharmaceuticals<br>- Teva Pharmaceutical Industries<br>- Sunovion Pharmaceuticals
				divalproex	generic versions			
				olanzapine	generic versions			
				risperidone	generic versions			
				quetiapine	generic versions			
				quetiapine XR	generic versions			
				aripiprazole	Bristol-Myers Squibb	Abilify		
				ziprasidone	Pfizer	Zeldox		
				lamotrigine	generic versions			
				bupropion	generic versions			
18	2013	2013	Management of Small Cell Carcinoma of the Bladder: Consensus Guidelines from the Canadian Association of Genitourinary Medical Oncologists (CAGMO)	cisplatin	generic versions		Canadian Association of Medical Oncologists (CAMO) [4]	- None identified
				carboplatin	generic versions			
				etoposide	generic versions			
				irinotecan	generic versions			
27	2013	2013	Canadian Network for Mood and Anxiety Treatments (CANMAT) and International Society for Bipolar Disorders (ISBD) Collaborative	lithium	generic versions		Canadian Network for Mood and Anxiety Treatments (CANMAT) [2]	- None identified
				divalproex	generic versions			
				divalproex ER	generic versions			
				olanzapine	generic versions			
				risperidone	generic versions			

[1] Excludes corporate sponsorship of conferences.

			Update of CANMAT Guidelines for the Management of Patients with Bipolar Disorder: Update 2013	quetiapine	generic versions			
				quetiapine XR	generic versions			
				aripiprazole	Bristol-Myers Squibb	Abilify		
				ziprasidone	Pfizer	Zeldox		
				asenapine	Merck	Saphris		
				paliperidone ER	Janssen	Invega		
				lamotrigine	generic versions			
				bupropion	generic versions			
29	2013	2013	The 2012 Canadian Cardiovascular Society Heart Failure Management Guidelines Update: Focus on Acute and Chronic Heart Failure	furosemide	generic versions		Canadian Cardiovascular Society (CCS) [5–7]	- AstraZeneca Canada Inc.<br>- PendoPharm<br>- Servier Canada Inc.<br>- Pfizer<br>- Bristol Myers Squibb<br>- Bayer<br>- Boehringer Ingelheim<br>- Merck<br>- Amgen
				nitroglycerin	generic versions			
				nesiritide	Janssen	Natrecor		
				nitroprusside	Hospira	Nipride		
				tolvaptan	Otsuka	Samsca		
				eplerenone	Pfizer	Inspra		
				spironolactone	generic versions			
				metolazone	Sanofi-Aventis	Zaroxolyn		
				digoxin	generic versions			
				isosorbide dinitrate	generic versions			
				hydralazine	generic versions			
				Aspirin	generic versions			
35	2013	2013	Bortezomib in Multiple Myeloma and Lymphoma	bortezomib	Janssen	Velcade	Cancer Care Ontario (CCO) [8,9]	- None identified
				melphalan	Triton Pharma	Alkeran		
				prednisone	generic versions			
				dexamethasone	generic versions			
				pegylated liposomal doxorubicin	Roche, Merck	Peginterferon, Pegasys, Pegasys RBV (Pegasys and Copegus), Pegetron, Unitron PEG		
40	2013	2013	Recommendations for Antithrombotic Agents for the Prevention of Stroke and Systemic Embolism in Patients with Atrial Fibrillation	acetylsalicylic acid	generic versions		Canadian Agency for Drugs and Technologies in Health (CADTH) [10]	- None identified
				clopidogrel	generic versions			
				warfarin	generic versions			
				dabigatran	Boehringer-Ingelheim	Pradax		
				rivaroxaban	Bayer	Xarelto		
				apixaban	Bristol-Myers Squibb	Eliquis		
44	2013	2013	CUA-CUOG Guidelines for the	abiraterone acetate	Janssen	Zytiga	Canadian Canadian	- Astellas<br>- Actavis
				prednisone	generic versions			

			Management of Castration-Resistant Prostate Cancer (CRPC): 2013 Update	docetaxel	Hospira	Taxotere	Urological Association (CUA) [11]	- GlaxoSmithKline<br>- Pfizer
				cabazitaxel	Sanofi-Aventis	Jevtana		
				enzalutamide	Astellas	Xtandi		
				denosumab	Amgen	Prolia, Xgeva		
				zolendronic acid	Novartis Pharmaceuticals	Aclasta, Zometa concentrate		
				calcium	generic versions			
				vitamin D	generic versions			
46	2013	2013	Management of Advanced Kidney Cancer: Canadian Kidney Cancer Forum 2013 Consensus Update	high-dose interleukin-2 (HD IL-2)	Novartis Pharmaceuticals	Proleukin, aldesleukin	Kidney Cancer Research Network of Canada,[12] Kidney Cancer Canada [13]	Kidney Cancer Research Network of Canada:<br>- Password protected<br><br>Kidney Cancer Canada<br>- Astellas Pharma Canada<br>- AstraZeneca<br>- Bayer Inc.<br>- Boehringer Ingelheim Canada<br>- Rx&D<br>- Celgene Canada<br>- Eli Lilly & Co.<br>- GlaxoSmithKline<br>- Janssen<br>- Novartis<br>- Pfizer<br>- Roche
				pazopanib	GlaxoSmithKline	Votrient		
				tivozanib	not found in CPS or DPD			
				temsirolimus	Pfizer	Torisel		
				bevacizumab	Roche	Avastin		
				everolimus	Novartis Pharmaceuticals	Afinitor		
				sorafenib	Bayer	Nexavar		
				sunitinib	Pfizer	Sutent		
				insulin NPH	Eli Lilly, NovoNordisk	Humulin-N, Novolinge NPH		
93	2013	2013	Canadian Telestroke Action Collaborate Telestroke Implementation Toolkit October 2013	alteplase (tPA)	Roche	Activase rtPA, Cathflo	Heart and Stroke Foundation [14]	- Bayer Inc.<br>- Boehringer Ingelheim<br>- Bristol-Myers Squibb<br>- Pfizer<br>- Janssen<br>- Allergan<br>- Merz
94	2013	2013	Canadian Headache Society Guideline Acute Drug Therapy for Migraine Headache	acetaminophen	generic versions		Headache Network Canada [15]	- Pfizer
				almotriptan	McNeil Consumer Healthcare	Axert		
				acetylsalicylic acid	generic versions			
				diclofenac potassium	generic versions			
				dihydroergotamine	generic versions			
				eletriptan	Pfizer	Relpax		
				ergotamine	Novartis Pharmaceuticals	Cafergot		
				frovatriptan	generic versions			
				ibuprofen	generic versions			
				metoclopramide	generic versions			

				naproxen sodium	generic versions			
				naratriptan	generic versions			
				rizatriptan	generic versions			
				sumatriptan	generic versions			
				zolmitriptan	generic versions			
				domperidone	generic versions			
				codeine	generic versions			
				tramadol	generic versions			
103	2013	2013	Canadian Cardiovascular Society Guidelines on the Use of Cardiac Resynchronization Therapy: Implementation	warfarin	generic versions		Canadian Cardiovascular Society (CCS) [5–7]	- AstraZeneca Canada Inc.<br>- PendoPharm<br>- Servier Canada Inc.<br>- Pfizer<br>- Bristol Myers Squibb<br>- Bayer<br>- Boehringer Ingelheim<br>- Merck<br>- Amgen
112	2012	2012	The Use of Antiviral Drugs for Influenza: Guidance for Practitioners 2012/2013	oseltamivir (Tamiflu "by Hoffman-La Roche Ltd, Canada)"	Roche		Association of Medical Microbiology and Infectious Disease Canada [16]	- None identified
				zanamivir	GlaxoSmithKline	Relenza		
242	2012	2012	Focused 2012 Update of the Canadian Cardiovascular Society Atrial Fibrillation Guidelines: Recommendations for Stroke Prevention and Rate/Rhythm Control	acetylsalicylic acid	generic versions		Canadian Cardiovascular Society (CCS) [5–7]	- AstraZeneca Canada Inc.<br>- PendoPharm<br>- Servier Canada Inc.<br>- Pfizer<br>- Bristol Myers Squibb<br>- Bayer<br>- Boehringer Ingelheim<br>- Merck<br>- Amgen
				dabigatran	Boehringer-Ingelheim	Pradax		
				rivaroxaban	Bayer	Xarelto		
				apixaban	Bristol-Myers Squibb	Eliquis		
				clopidogrel	Sanofi-Aventis	Plavix		
				diltiazem	generic versions			
				verapamil	generic versions			
				digoxin	generic versions			
				dronedarone	Sanofi-Aventis	Multaq		
				flecainide	Graceway	Tambocor		
				propafenone	Sanis Health, Abbott	Propafenone (Sanis Health); Rythmol (Abbott)		
				sotalol	generic versions			
				amiodarone	generic versions			
244	2012	2013	Management of Patients with Refractory Angina: Canadian	allopurinol	generic versions		Canadian Cardiovascular Society (CCS) [5–7]	- AstraZeneca Canada Inc.<br>- PendoPharm<br>- Servier Canada Inc.<br>- Pfizer
				ranolazine	not found in CPS or DPD			
				trimetazidine	not found in CPS or DPD			

			Cardiovascular Society/Canadian Pain Society Joint Guidelines	nicorandil	not found in CPS or DPD			- Bristol Myers Squibb<br>- Bayer<br>- Boehringer Ingelheim<br>- Merck<br>- Amgen
				ivabradine	not found in CPS or DPD			
258	2012	2012	Canadian Ophthalmological Society Evidence-Based Clinical Practice Guidelines for the Management of Diabetic Retinopathy	ranibizumab	Novartis Pharmaceuticals	Lucentis	Canadian Ophthalmological Society [17,18]	- None identified
260	2012	2012	Managing Cardiometabolic Risk in Primary Care	metformin	generic versions		None clearly identified	- n/a
				bupropion	generic versions			
				varenicline	Pfizer	Champix		
267	2012	2012	Evidence-Based Guideline for Neuropathic Pain Interventional Treatments: Spinal Cord Stimulation, Intravenous Infusions, Epidural Injections, and Nerve Blocks	lidocaine	AstraZeneca, Hospira, Odan	EMLA Cream (AstraZeneca); EMLA Patch (AstraZeneca); Lidocaine Parenteral - Antiarrhythmic Agent (Hospira); Lidodan Endotracheal (Odan); Lidodan Viscous (Odan); Xylocaine Jelly 2% (AstraZeneca); Xylocaine Parenteral Without Epinepherine (AstraZeneca); Xylocaine Topical 4% (AstraZeneca); Xylocaine Viscous 2% (AstraZeneca); Xylocard (AstraZeneca)	Canadian Pain Society (CPS) [19]	- None identified
269	2012	2012	Lenalidomide in	lenalidomide	Celgene	Revlimid	Cancer Care	- None identified

			Multiple Myeloma	dexamethasone	Alcon, Sanofi-Aventis	dexamethasone/ciprofloxacin HCl = Ciprodex (Alcon); dexamethasone/framycetin sulfate/gramicidin = Sofracort (Sanofi-Aventis); dexamethasone/neomycin sulfate/polumixin B sulfate = Maxitrol (Alcon); dexamethasone/tobramycin = Tobradex (Alcon)	Ontario (CCO) [8,9]	
				acetylsalicylic acid	generic versions			
				enoxaparin	Sanofi-Aventis	Lovenox; Lovenox HP		
				granulocyte-colony stimulating factor (GCSF)	Amgen	Neupogen (filgrastim)		
273	2012	2012	Risk Reduction of Prostate Cancer with Drugs or Nutritional Supplements	finasteride	generic versions		Cancer Care Ontario (CCO) [8,9]	- None identified
				dutasteride	GlaxoSmithKline	Avodart		
274	2012	2012	The 2012 Canadian Hypertension Education Program Recommendations for the Management of Hypertension: Blood Pressure Measurement, Diagnosis, Assessment of Risk, and Therapy	acetylsalicylic acid	generic versions		Hypertension Canada,[20] Canadian Pharmacy Association,[21] Canadian Council of Cardiovascular Nurses,[22] Heart and Stroke Foundation of Canada[14]	Hypertension Canada<br>- Servier<br>- Pfizer<br>- Abbott<br>- Merck<br><br>Canadian Pharmacy Association<br>- None identified<br><br>Canadian Council of Cardiovascular Nurses<br>- AstraZeneca<br><br>Heart and Stroke Foundation<br>- Bayer Inc.<br>- Boehringer Ingelheim<br>- Bristol-Myers Squibb<br>- Pfizer<br>- Janssen<br>- Allergan<br>- Merz
283	2012	2012	New Oral	dabigatran	Boehringer-Ingelheim	Pradax	Canadian Agency	- None identified

			Anticoagulants for the Prevention of Thromboembolic Events in Patients with Atrial Fibrillation	rivaroxaban	Bayer	Xarelto	for Drugs and Technologies in Health (CADTH) [10]	
289	2012	2012	The Role of Liver Resection in Colorectal Cancer Metastases	oxaliplatin	Sanofi-Aventis	Eloxatin	Cancer Care Ontario (CCO) [8,9]	- None identified
				irinotecan	Pfizer	Camptosar		
295	2012	2012	Chemotherapy (i.e., Gemcitabine, Docetaxel Plus Gemcitabine, Doxorubicin, or Trabectedin) for Inoperable, Locally Advanced, Recurrent, or Metastatic Uterine Leiomyosarcoma	doxorubicin	Janssen	Caelyx	Cancer Care Ontario (CCO) [8,9]	- None identified
				Gemcitabine	generic versions			
				Docetaxel	Hospira, Sanofi-Aventis	docetaxel, Taxotere		
				granulocyte-colony stimulating factor (GCSF)	Amgen	Neupogen (filgrastim)		
299	2012	2012	Canadian Rheumatology Association Recommendations for the Pharmacological Management of Rheumatoid Arthritis with Traditional and Biologic Disease-modifying Antirheumatic Drugs: Part II Safety	methotrexate	Hospira, Pfizer, Medexus	Methotrexate, Methotrexate injection, Methotrexate tablets, Metoject	Canadian Rheumatology Association [23]	- Abbvie Corporation<br>- Pfizer Canada<br>- Amgen Canada Inc.<br>- Celgene Inc.<br>- Janssen Inc.<br>- Roche Canada<br>- Bristol-Myers Squibb Canada Co.<br>- GlaxoSmithKline Inc.<br>- Hospira<br>- UCB Canada Inc.<br>- AstraZeneca Canada Inc.<br>- Eli Lilly Canada Inc.
				abatacept	Bristol-Myers Squibb	Orencia		
				tocilizumab	Roche	Actemra		
				influenza vaccine	Abbott, Sanofi-Pasteur, Novartis, AstraZeneca	FluMist, Intanza, Agriflu, Influvac, Fluad		
				pneumococcal vaccine	Sanofi-Pasteur, Merck, Pfizer, GlaxoSmithKline	Pneumo 23, Prevnar 13, Synflorix, Pneumovax 23		
				hepatitis B vaccine	GlaxoSmithKline, Merck	Energix-B, Recombivax HB		
				herpes zoster vaccine	not found - unspecific			
				hydroxychloroquine	Sanofi-Aventis	Plaquenil		
				sulfasalazine	generic versions			
				rituximab	Roche	Rituxan		
345	2012	2012	Neonatal Abstinence Syndrome Clinical Practice Guidelines for Ontario	methadone	Paladin	Metadol	None clearly identified	- n/a
				buprenorphine	Purdue Pharma, RB Pharmaceuticals	Suboxone, BuTrans5, BuTrans10, BuTrans20		
				morphine	generic versions			

349	2012	2012	Management of Chronic Hepatitis B: Canadian Association for the Study of the Liver Consensus Guidelines	hepatitis B vaccine	GlaxoSmithKline, Merck	Energix-B, Recombivax HB	Canadian Liver Foundation [24]	- Abbvie<br>- Astellas<br>- Bayer HealthCare Pharmaceuticals<br>- Bristol-Myers Squibb<br>- Gilead Sciences<br>- Janssen<br>- BTG<br>- Merck<br>- Vertex Pharmaceuticals
				PEG IFN	Roche, Merck	Peginterferon, Pegasys, Pegasys RBV (Pegasys and Copegus), Pegetron, Unitron PEG		
				tenofovir	Gilead Sciences	Viread, Atripla, Truvada		
				entecavir	Bristol-Myers Squibb	Baraclude		
				lamivudine	ViiV Healthcare Shire Canada	3TC, Heptovir, Kivexa, Trizivir, Combivir		
				emtricitabine	Gilead Sciences	Atripla, Truvada		
				hepatitis immune globulin	Talecris	HyperHEP B SD		
352	2012	2012	Recommendations of the 4th Canadian Consensus Conference on the Diagnosis and Treatment of Dementia	risperidone	generic versions		Canadian Institutes of Health Research, Fonds de la recherche en santé du Québec, Alzheimer Society of Canada, Consortium of Canadian Centres for Clinical Cognitive Research, Canadian Neurological Society, Canadian Academy of Geriatric Psychiatry, Quebec Network for Research on Aging, and Canadian Geriatrics Society [25–33]	Canadian Institutes of Health Research<br>- None identified<br><br>Fonds de la recherche en santé du Québec<br>- AllerGen NCE Inc.<br>- Merck Sharp & Dohme<br>- Pfizer Canada Inc.<br><br>Alzheimer Society of Canada<br>- Pfizer<br>- Rx&D<br><br>Consortium of Canadian Centres for Clinical Cognitive Research<br>- None identified<br><br>Canadian Neurological Society<br>- Biogen Idec<br>- MS<br>- Novartis Pharmaceuticals<br>- Biocodex<br>- Allergan<br>- Eisai<br>- Grifols<br>- UCB<br>- Teva<br>- EMD Serono<br>- Sunovion<br>- Genzyme<br><br>Canadian Academy of Geriatric
				olanzapine	generic versions			
				aripiprazole	Bristol-Myers Squibb	Abilify		

								Psychiatry<br>- None identified<br>Quebec Network for Research on Aging<br>- None identified<br><br>Canadian Geriatrics Society<br>- None identified

References

1. EPICORE Centre Epidemiology Coordinating and Research Centre. Sponsors and Collaborators [Internet]. 2007 [cited 2015 Apr 10]. Available from: http://www.epicore.ualberta.ca/compris/COMPRISSponsors.html

2. Canadian Network for Mood and Anxiety Treatments (CANMAT). Welcome to CANMAT [Internet]. 2015 [cited 2015 May 15]. Available from: http://www.canmat.org

3. International Society for Bipolar Disorders (ISBD). Fiscal 2012 Annual Report [Internet]. 2013 [cited 2015 May 15]. Available from: http://www.isbd.org/images/PDF/Annual_Report_2012.pdf

4. Canadian Association of Medical Oncologists (CAMO). Home Page [Internet]. 2013 [cited 2015 May 7]. Available from: http://cos.ca/camo/

5. Canadian Cardiovascular Society (CCS). CCSA Donations: October 1, 2013-September 30, 2014 [Internet]. 2014 [cited 2015 May 7]. Available from: http://www.ccs.ca/images/Academy/Donor_Slides_Final_for_web.pdf

6. Canadian Cardiovascular Society (CCS). Recognizing our Donors [Internet]. 2015 [cited 2015 May 7]. Available from: http://www.ccs.ca/index.php/en/ccs-academy/recognizing-our-donors

7. Canadian Cardiovascular Society (CCS). Guideline Resources [Internet]. 2014 [cited 2015 May 15]. Available from: http://ccs.ca/index.php/en/guideline-resources

8. Cancer Care Ontario (CCO). Financial Statements [Internet]. 2013. Available from: https://www.cancercare.on.ca/common/pages/UserFile.aspx?fileId=297935

9. Cancer Care Ontario (CCO). Cancer Care Ontario Financial Statements [Internet]. 2014. Available from: https://www.cancercare.on.ca/common/pages/UserFile.aspx?fileId=319494

10. Canadian Association for Drugs and Technologies in Health (CADTH). Sponsors and Exhibitors [Internet]. 2015 [cited 2015 May 7]. Available from: https://www.cadth.ca/sponsors-and-exhibitors

11. Canadian Urological Association (CUA). Homepage [Internet]. 2015 [cited 2015 May 7]. Available from: https://www.cua.org/en

12. Kidney Cancer Research Network of Canada (KCRNC). Our Supporters [Internet]. 2015 [cited 2015 May 7]. Available from: https://www.kcrnc.ca/main.php?action=login&go=/main.php?p=89

13. Kidney Cancer Canada (KCC). Our Partners and Sponsors [Internet]. 2013 [cited 2015 May 7]. Available from: http://www.kidneycancercanada.ca/about-us/our-partners-and-sponsors/

14. Heart and Stroke Foundation. Our National Corporate Sponsors [Internet]. 2015 [cited 2015 May 7]. Available from: http://www.heartandstroke.com/site/c.ikIQLcMWJtE/b.4389517/k.3AF2/Our_National_Corporate_Partners.htm

15. Headache Network Canada (HNC). Home [Internet]. 2012 [cited 2015 May 7]. Available from: http://headachenetwork.ca/

16. Association of Medical Microbiology and Infectious Disease Canada. Home [Internet]. 2011 [cited 2015 May 7]. Available from: http://www.ammi.ca/

17. Canadian Ophthalmological Society (COS). COS Affiliates [Internet]. 2015 [cited 2015 May 7]. Available from: http://www.cos-sco.ca/about-cos/cos-affiliates/

18. Canadian Ophthalmological Society (COS). Partners & Supporters [Internet]. 2015 [cited 2015 May 7]. Available from: http://www.cos-sco.ca/about-cos/partners-supporters/

19. Canadian Pain Society (CPS). Home [Internet]. 2015 [cited 2015 May 7]. Available from: http://rheum.ca/en/events/upcoming_events/sponsors_and_exhibitors1

20. Hypertension Canada. Supporters and Corporate Partners [Internet]. 2014 [cited 2015 May 7]. Available from: https://www.hypertension.ca/en/supporters-and-corporate-partners

21. Canadian Pharmacists Association (CPA). Partnerships [Internet]. 2015 [cited 2015 May 7]. Available from: http://www.pharmacists.ca/index.cfm/about-cpha/partnerships/

22. Canadian Council for Cardiovascular Nurses (CCCN). Sponsors [Internet]. 2015 [cited 2015 May 1]. Available from: https://www.cccn.ca/content.php?doc=14

23. Canadian Rheumatology Association (CRA). Sponsors and Exhibitors [Internet]. 2015 [cited 2015 May 7]. Available from: http://rheum.ca/en/events/upcoming_events/sponsors_and_exhibitors1

24. Canadian Liver Foundation. National Partners & Sponsors [Internet]. 2015 [cited 2015 May 7]. Available from: http://www.liver.ca/who-we-are/National-Partners-Sponsors.aspx

25. Canadian Consensus Conference on the Diagnosis and Treatment of Dementia (CCCDTD) - 2012. 2012 Canadian Consensus Conference on Dementia [Internet]. 2012 [cited 2015 May 7]. Available from: http://www.cccdtd.ca/

26. Canadian Institute for Health Information (CIHI). About CIHI [Internet]. 2014 [cited 2015 May 7]. Available from: http://www.cihi.ca/CIHI-ext-portal/internet/EN/Theme/about+cihi/cihi010702

27. Fonds de la recherche en santé du Québec. Nos Partenaires [Internet]. Wall Street Journal. 2015 [cited 2015 May 2]. Available from: http://www.frqs.gouv.qc.ca/partenariat/nos-partenaires

28. Alzheimer Society of Canada. Our Partners [Internet]. 2015 [cited 2015 May 7]. Available from: http://www.alzheimer.ca/en/About-us/Our-partners

29. Consortium of Canadian Centres for Clinical Cognitive Research (C5R). About [Internet]. 2015 [cited 2015 May 7]. Available from: http://c5r.ca/aboutus/

30. Canadian Neurological Sciences Federation. Our Generous Sponsors [Internet]. 2015 [cited 2015 May 7]. Available from: http://www.cnsfederation.org/sponsors/

31. Canadian Academy of Geriatric Psychiatry (CAGP). About CAGP [Internet]. 2015 [cited 2015 May 7]. Available from: http://www.cagp.ca/

32. Quebec Network for Research on Aging. Partners [Internet]. 2015 [cited 2015 May 7]. Available from: http://www.rqrv.com/en/partenariats.php

33. Canadian Geriatrics Society. Donor Recognition Wall [Internet]. 2015 [cited 2015 May 7]. Available from: http://www.canadiangeriatrics.ca/default/assets/File/CGS_Donations_September_2014_wall.pdf
